# Differential requirements of tubulin genes in mammalian forebrain development

**DOI:** 10.1371/journal.pgen.1008243

**Published:** 2019-08-06

**Authors:** Elizabeth Bittermann, Zakia Abdelhamed, Ryan P. Liegel, Chelsea Menke, Andrew Timms, David R. Beier, Rolf W. Stottmann

**Affiliations:** 1 Division of Human Genetics, Cincinnati Children’s Hospital Medical Center, Cincinnati, Ohio, United States of America; 2 Department of Anatomy and Embryology, Faculty of Medicine (Girl’s Section), Al-Azhar University, Cairo, Egypt; 3 Center for Developmental Biology and Regenerative Medicine, Seattle Children’s Research Institute, Seattle, Washington, United States of America; 4 Department of Pediatrics, University of Washington Medical School, Seattle, Washington, United States of America; 5 Division of Developmental Biology, Cincinnati Children’s Hospital Medical Center, Cincinnati, Ohio, United States of America; 6 Department of Pediatrics, University of Cincinnati College of Medicine, Cincinnati, Ohio, United States of America; University of Colorado School of Medicine, UNITED STATES

## Abstract

Tubulin genes encode a series of homologous proteins used to construct microtubules which are essential for multiple cellular processes. Neural development is particularly reliant on functional microtubule structures. Tubulin genes comprise a large family of genes with very high sequence similarity between multiple family members. Human genetics has demonstrated that a large spectrum of cortical malformations are associated with *de novo* heterozygous mutations in tubulin genes. However, the absolute requirement for many of these genes in development and disease has not been previously tested in genetic loss of function models. Here we directly test the requirement for *Tuba1a*, *Tubb2a* and *Tubb2b* in the mouse by deleting each gene individually using CRISPR-Cas9 genome editing. We show that loss of *Tubb2a* or *Tubb2b* does not impair survival but does lead to relatively mild cortical malformation phenotypes. In contrast, loss of *Tuba1a* is perinatal lethal and leads to significant forebrain dysmorphology. We also present a novel mouse ENU allele of *Tuba1a* with phenotypes similar to the null allele. This demonstrates the requirements for each of the tubulin genes and levels of functional redundancy are quite different throughout the gene family. The ability of the mouse to survive in the absence of some tubulin genes known to cause disease in humans suggests future intervention strategies for these devastating tubulinopathy diseases.

## Author summary

Tubulin proteins are assembled into microtubules to provide essential cellular cytoskeletal elements. Microtubules are especially crucial for neuronal development. Multiple studies demonstrate that human malformations of cortical development are caused by genetic variants in several tubulin genes. Interestingly, most of these developmental phenotypes are the result of *de novo*, heterozygous variants likely to act as dominant negatives interfering with normal tubulin function(s). The roles of individual tubulin genes and the mechanism(s) leading to these malformations remain unclear. Genome sequencing efforts revealed high sequence similarity between many tubulin genes, raising the possibility of functional compensation. The requirements for many of these tubulin genes have not been previously addressed in loss of function experiments. We have generated novel deletions of several tubulin genes known to cause human disease to assess if they are required for brain development, or if the human variants act to alter function of the tubulin proteins leading to the pathogenesis. Surprisingly, our results show that some individual tubulin genes are absolutely required for survival while others are not and have much more benign cortical malformations.

## Introduction

Tubulin proteins are fundamental building blocks of the cell and assemble into dynamic microtubules. Microtubules are especially crucial for cortical development where they are used in multiple cellular contexts such as the mitotic spindle, axons, dendrites, and cilia formation [1]. Mutations in tubulin genes are now known to cause multiple human cortical malformations including lissencephaly, polymicrogyria, microcephaly, dysmorphic basal ganglia, and congenital fibrosis of extraocular muscles [2]. These are collectively discussed as “tubulinopathies.” Many variants leading to malformations of cortical development have now been identified in *TUBULIN*, *ALPHA-1A (TUBA1A)* [2–18]*; TUBULIN*, *BETA-2A (TUBB2A)* [19–22]; *TUBB2B* [4, 5, 15, 23–29]; *TUBB3* [2, 15, 30, 31]; *TUBB4A* [32]; and *TUBB/TUBB5* [33]. In fact, for *TUBA1A* and *TUBB2B* alone, there are now 71 identified variants in cortical malformation patients [7–29, 34]. Mutations of *TUBA1A* and *TUBB2B* account for ~5% and ~1.2% of lissencephaly and related malformations of cortical development, respectively [34]. Both, but especially *TUBA1A*, are associated with a wide spectrum of phenotypic severity [34]. With the exception of one *TUBA1A* variant inherited from a mosaic parent [6] and one inherited *TUBB2B* variant [24], all of these variants are heterozygous, *de novo* changes in the identified proband. TUBB3 variants have been found to segregate as dominant traits in large pedigrees [31].

Previous work has shown that both α- and β- tubulins can be separated into different classes (isotypes) and some general characteristics about their spatiotemporal expression domains have been established [35, 36]. However, the completed genome sequences of many species, including mouse and human, have revealed the presence of more tubulin proteins than those indicated by the initial six classes. The requirements and roles in cortical development for some of the individual tubulin genes are still unknown. One possible mechanism of disease in the tubulinopathies is the production of altered tubulin monomers from the variant alleles which alter normal tubulin function(s) in any number of cellular contexts. Experiments overexpressing these pathogenic variants and/or loss of function models in mouse and cells have indeed shown defects in microtubule polymerization and heterodimer assembly, neurite extension, growth cone dynamics, neuronal migration, vesicular axon transport, and peripheral nerve regeneration, among other processes [7, 11, 15, 24, 26, 30, 31, 37–40].

Most tubulin genes exhibit high sequence homology and many are clustered in the genome. Complete genome assemblies now highlight that *TUBB2A* and *TUBB2B* are immediately adjacent to each other in both the human and mouse genome and are virtually identical (443 identical amino acids of 445 total). Similarly, *TUBA1A*, *TUBA1B*, and *TUBA1C* genes are also adjacent in the genome and contain high sequence homology in both human and mouse. These characteristics suggest these genes may be the product of genome duplications and the functions of each individual gene may be shared across the cluster(s).

Despite the central importance of these genes in the cytoskeleton and their relevance for human cortical malformation, relatively few genetic models of tubulin variants exist. No null alleles are published for *Tuba1a*, *Tuba1b*, *Tuba1c*, *Tubb2a*, or *Tubb2b*. ENU mutagenesis efforts have identified alleles in *Tuba1a* [7, 40, 41] and *Tubb2b* [42], and a mouse model of CFEOM for *Tubb3* has been made [31]. More recently a null allele of *Tubb3* has been produced and shown to have effects on growth cone function and axonal regeneration after induced sciatic nerve injury [37]. Here we test the requirements for *Tuba1a*, *Tubb2a*, and *Tubb2b* with specific genetic deletions.

## Results

### Null alleles of *Tuba1a*, *Tubb2a*, and *Tubb2b*

**Fig 1:**
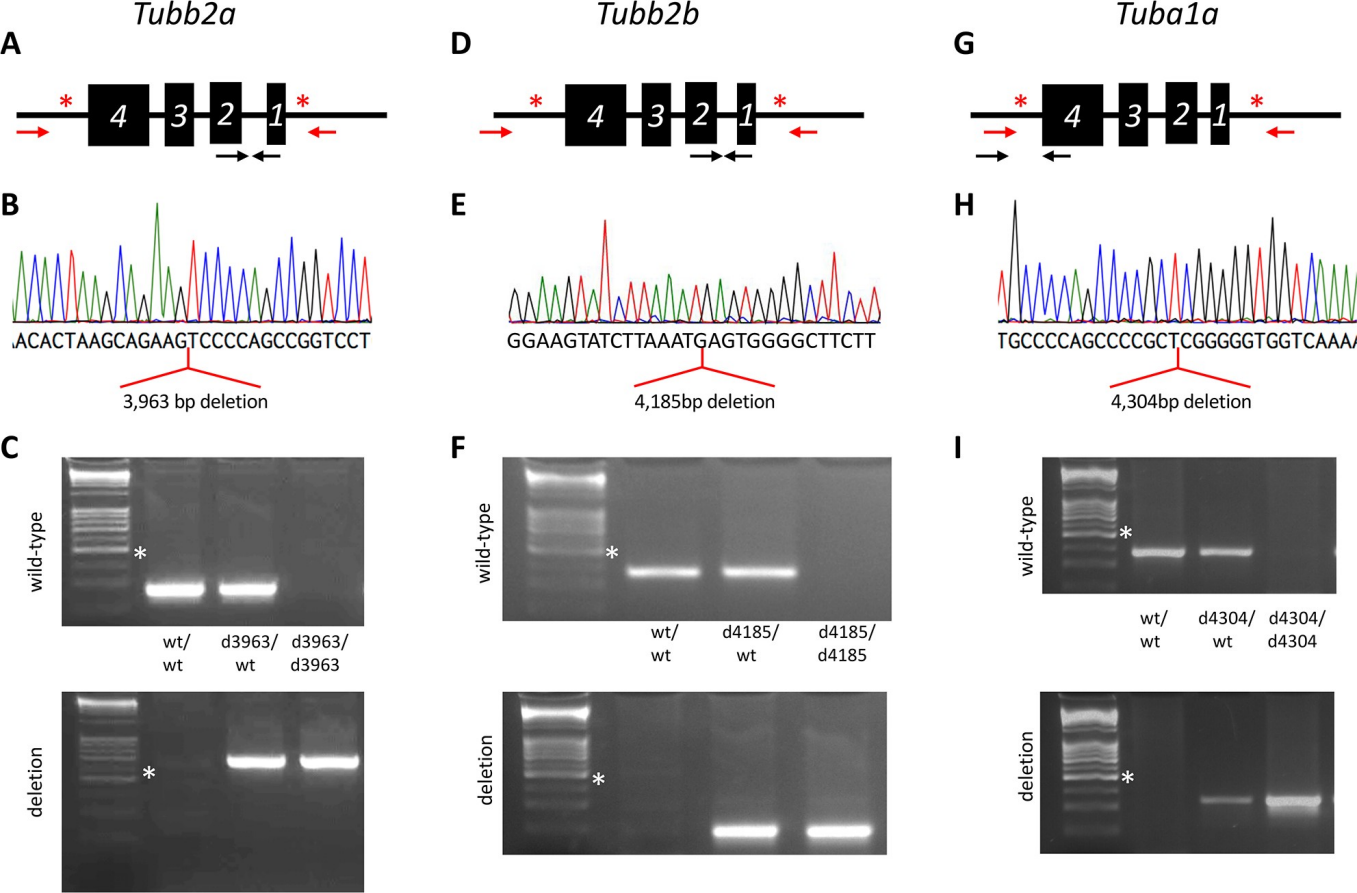
Novel CRISPR/CAS9 alleles of three tubulin genes. Deletion of *Tubb2a* (A-C), *Tubb2b* (D-F), and *Tuba1a* (G-I). (A,D,G) Schematics for deletions showing sites of CRISPR guide sequences (red asterisk), arrows indicate relative locations of PCR primers (red are meant to only amplify when gene deletion occurs under PCR standard conditions). (B,E,H) Sequencing chromatograms showing the results of mutant specific PCR products. Alignments to the reference genome indicate the deletions indicated for each gene. (C,F,I) PCR genotyping to specifically identify each allele. Note *Tubb2a*, *Tubb2b*, and *Tuba1a* are on the reverse strand of their respective chromosomes. White asterisk indicates 500 bp marker in DNA ladder.

We used CRISPR-Cas9 genome editing to create individual deletions of *Tuba1a*, *Tubb2a*, and *Tubb2b*. Guide RNAs were generated to target the endonuclease to two intergenic regions flanking each tubulin gene (Fig 1A, 1D and 1G; S1 Table). *Tubb2a* and *Tubb2b* are only 49kb apart (0.02cM) in the mouse genome, suggesting that meiotic recombination between the loci will be an extremely rare event. With this consideration in mind, we first attempted to multiplex CRISPR-Cas9 editing for *Tubb2a* and *Tubb2b* with the goal of creating a single deletion of each gene as well as a simultaneous deletion of both. A mix of all eight *Tubb2a* and *Tubb2b* guides were constructed and used for blastocyst microinjection. The resulting founders were analyzed by PCR and Sanger sequencing for alterations at the *Tubb2a* and/or *Tubb2b* loci. We recovered two alleles of *Tubb2a* and one of *Tubb2b*. *Tubb2a*^*em1Rstot*^ (hereafter referred to as *Tubb2a*^*d3963*^) is a 3,963bp deletion (chr13: 34,074,222–34,078,184; GRCm38/mm10) and *Tubb2a*^*em2Rstot*^ *(Tubb2a*^*d4222*^*)* is a 4,222bp deletion (chr13: 34,074,225–34,078,446). Both independent deletions excise the entire *Tubb2a* open reading frame (Fig 1A–1C, S1 and S2 Figs). We also obtained one complete deletion allele of *Tubb2b*. *Tubb2b*^*em1Rstot*^ (*Tubb2b*^*d4185*^) is a 4,185bp deletion (chr13: 34,126,374–34,130,558; Fig 1D–1F, S2 Fig).

In a parallel manner, four CRISPR guides were generated to delete *Tuba1a* (Fig 1G) and we recovered two independent deletions (Fig 1G–1I; S1 and S2 Figs). *Tuba1a*^*em1Rstot*^ (*Tuba1a*^*d4304*^) is a 4,304 bp deletion (chr15:98,949,728–98,954,031) and *Tuba1a*^*em2Rstot*^ (*Tuba1a*^*d4262*^) is a 4,262 bp deletion (chr15: 98,949,770–98,954,031). For all guides used in this study, we predicted the top ten off-target sites (S2 Table). Only one is even on the same chromosome as the desired target sequence. This potential target is 24 cM away from *Tuba1a*. At this genetic distance, there is virtually no chance this genomic sequence is segregating with the *Tuba1a* deletion allele we generated.

### Loss of *Tubb2a* or *Tubb2b* leads to postnatal forebrain phenotypes

**Fig 2:**
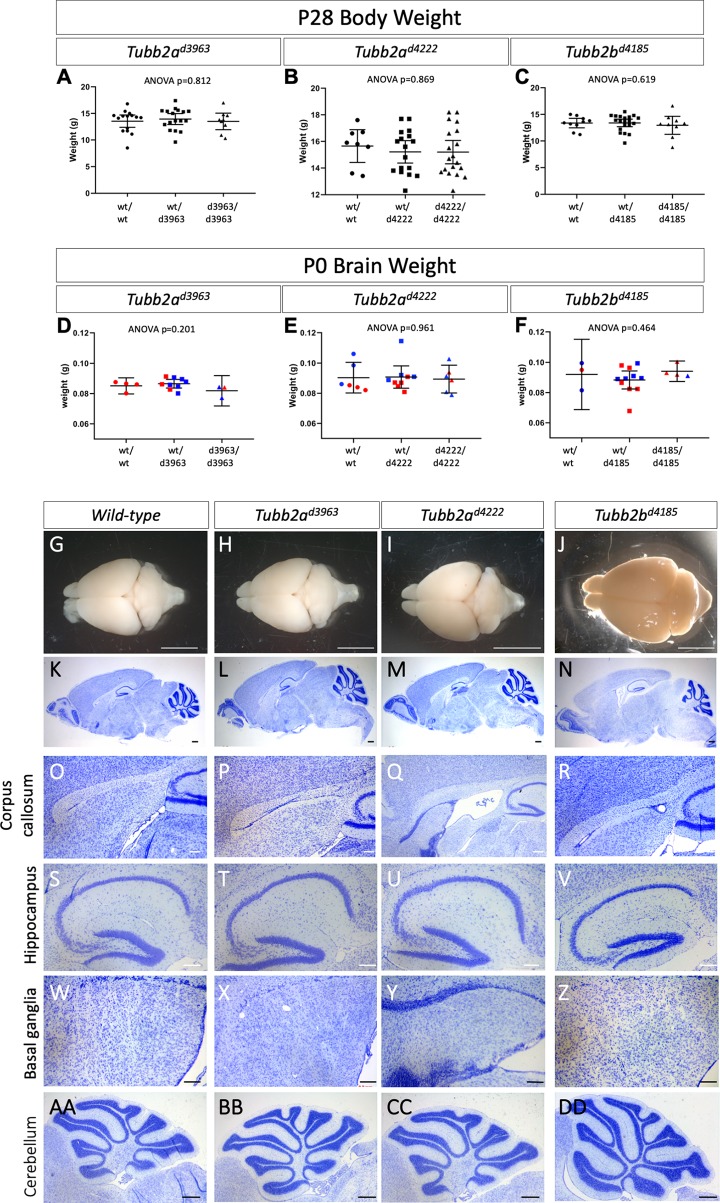
General morphology and histological analysis of *Tubb2a* and *Tubb2b* deletions. (A-C) Postnatal weights of the *Tubb2a* and *Tubb2b* mice at P28 show no sign of failure to thrive as compared to wild-type. (D-F) Brain weight at P0 is also unaffected by *Tubb2a* or *Tubb2b* deletion. Whole mount (G-J) and Nissl staining (K-DD) histological analysis of wild-type (G,K,O,S,W,AA), *Tubb2a*^*d3963/d3963*^ (H,L,P,T,X,BB), *Tubb2a*^*d4222/d4222*^ (I,M,Q,U,Y,CC) and *Tubb2b*^*d4185/d4185*^ (J,N,R,V,Z,DD) homozygous mice at P28-31. More detailed analysis of the corpus callosum (O-R), hippocampus (S-V), basal ganglia (W-Z), and cerebellum (AA-DD) is shown and all appear unaffected in overall size or organization. Scale bars indicate 1mm (G-J), 500 μm (K-N,AA-FF) and 200 μm (O-Z).

Mice heterozygous for all the deletion alleles were viable and fertile. As we hypothesized the deletion alleles would yield recessive phenotypes, we intercrossed heterozygous carriers for each. We found no reduction from Mendelian expectations in the number of live animals at weaning for any allele of *Tubb2a* or *Tubb2b*, and animals homozygous for these deletions did not appear grossly different than littermates (Table 1). Total body weight at postnatal day (P) 28 did not show any evidence of a failure to thrive upon loss of *Tubb2a* or *Tubb2b* (Fig 2A–2C, S3 Table). We collected brains for both whole mount analysis and histological examination from P28-31 animals to determine if loss of these tubulin genes caused any discernable malformations of cortical development. Total brain weight at P0 did not differ in *Tubb2a* or *Tubb2b* mutants (Fig 2D–2F, S3 Table). Whole mount imaging and initial histological analysis of the brains did not reveal any gross phenotypes (Fig 2G–2DD, S3 and S4 Figs). This included more detailed analyses of the corpus callosum (Fig 2O–2R), hippocampus (Fig 2S–2V), basal ganglia (Fig 2W–2Z) and cerebellum (Fig 2AA–2DD).

**Table 1:** Survival at weaning of mice with deletions of *Tubb2a* and Tubb2b*.

	wild-type	heterozygous	homozygous	total	p-value
*Tubb2a*^*d3963*^	43	66	41	151	0.33
*Tubb2a*^*d4222*^	24	42	20	87	0.81
*Tubb2b*^*d4185*^	46	87	37	170	0.59

* All data here are from heterozygous intercrosses

**Fig 3:**
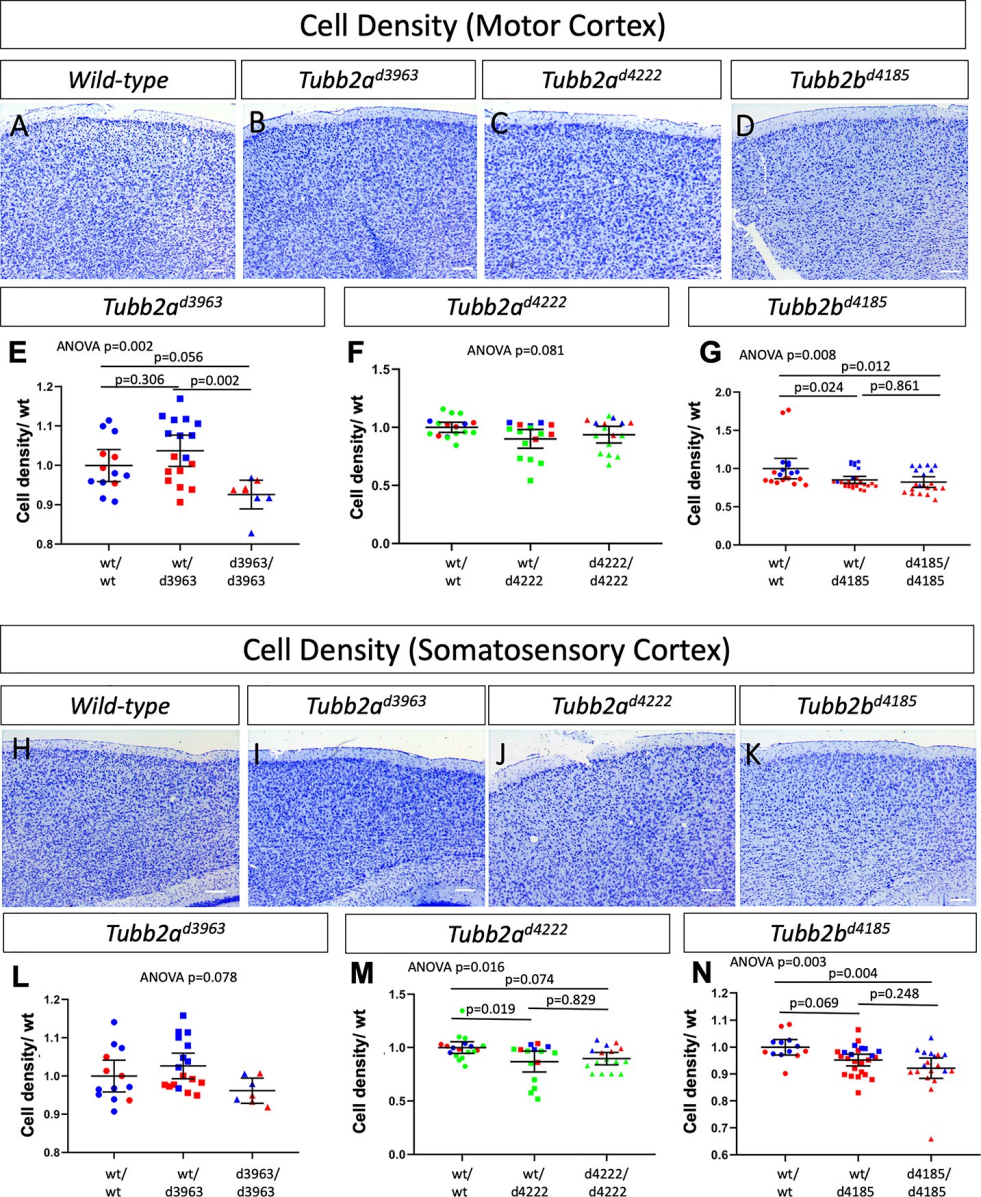
Quantitative analysis of cell density in the motor and somatosensory cortex in *Tubb2a and Tubb2b* deletions alleles. Quantification of Nissl-positive cells/area for both the motor cortex (A-G) and somatosensory (H-N) cortex show some changes in cellular density. ANOVA p values are shown for each experiment and multiple comparison p values are shown when ANOVA p≤0.05. (Individual samples are plotted to indicate sample size, differing colors in E-G, L-N indicate littermates. n = 3 wild-type, 3 heterozygotes and 3 homozygous mutants for *Tubb2a*^*d3963*^; 4 wild-type, 4 heterozygotes and 4 mutants for *Tubb2a*^*d4222*^; and 4 wild-type, 5 heterozygotes, and 4 mutants for *Tubb2b*^*d4185*^).

**Fig 4:**
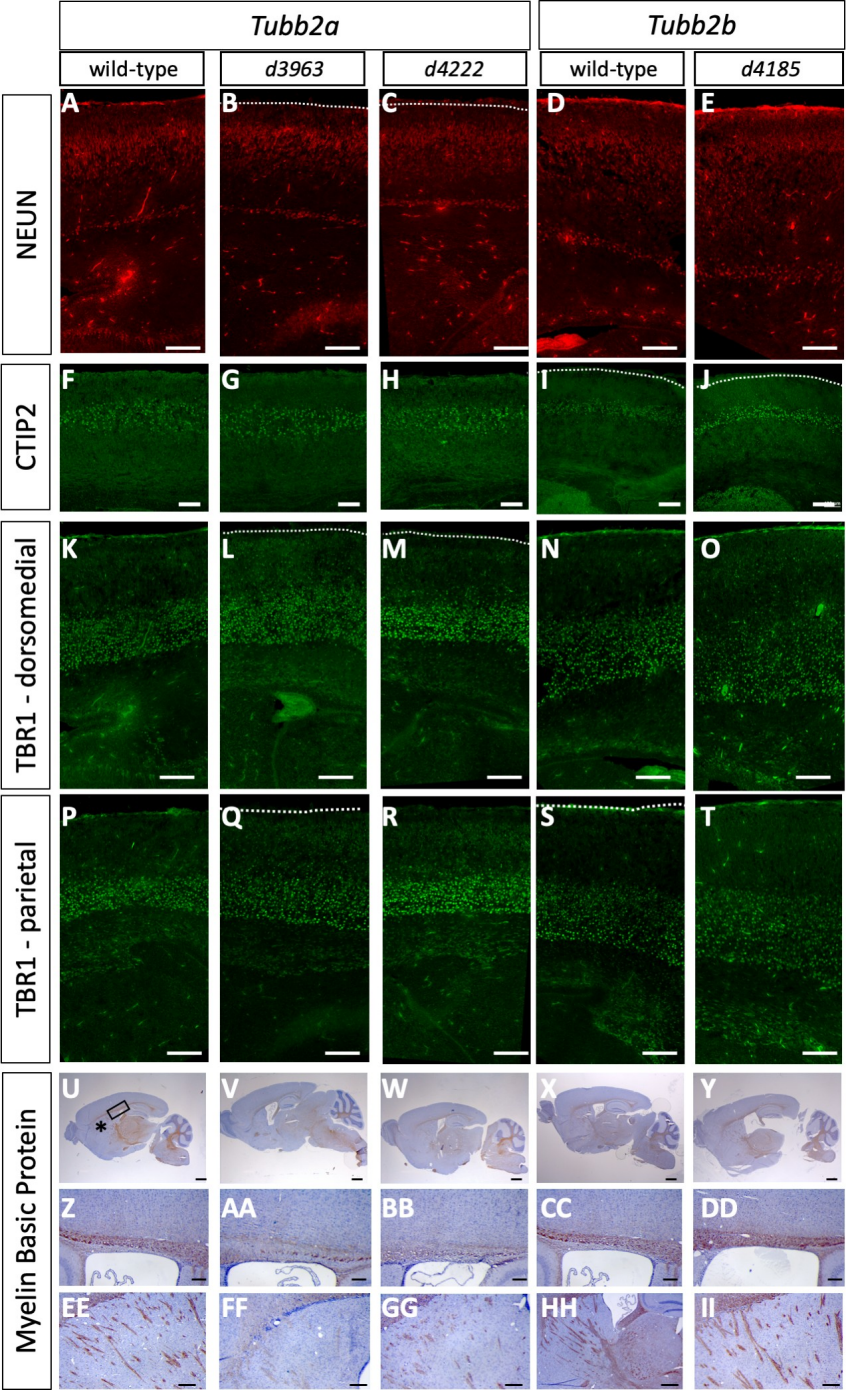
Cortical layering deficits in *Tubb2a* and *Tubb2b* deletion mutants. Immunohistochemistry for NeuN (A-E), CTIP2 (F-J), TBR1 (K-T) and myelin basic protein (MBP, U-II) highlights multiple phenotypes in *Tubb2a*^*d3963/d3963*^ (B,G,L,Q,V,AA,FF), *Tubb2a*^*d4222/d4222*^ (C,H,M,R,W,BB,GG) and *Tubb2b*^*d4185/d4185*^ (E,J,O,T,Y,DD,II) homozygous mice as compared to wild-type (A,D,F,I,K,N,P,S,U,X,Z,CC,EE,HH). (A-E) NeuN staining for differentiated neurons. (F-J) CTIP2-positive upper layer neurons. (K-T) TBR1-positive lower layer neurons. (U-II) MBP immunoreactivity indicates severe loss of axon tracts in the corpus callosum (AA, BB) and pyramidal fiber tracts (FF, GG) of the *Tubb2a* deletion, but not the *Tubb2b* deletion (DD,II). Box and asterisk in U indicate areas shown in Z and EE, respectively. Scale bars indicate 100 μm (A-T), 1 mm (U-Y), 200 μm (Z-DD) and 500 um(EE-II).

We performed a more comprehensive histological and immunohistochemical analysis which did reveal a number of more subtle anomalies in animals lacking tubulin genes (Figs 2–4, S3–S5 Figs). Quantification of the number of cells in the motor and somatosensory cortices as identified through Nissl staining did reveal some subtle differences (Fig 3, S3 Fig, S3 Table). The *Tubb2a*^*d3963/d3963*^, *Tubb2a*^*d4222/d4222*^ and *Tubb2b*^*d4185/d4185*^ homozygous mice all do appear to have slightly decreased cell numbers in both the motor (Fig 3A–3G) and somatosensory (Fig 3H–3N) cortical tissues, albeit with differing levels of statistical certainty.

We complemented this histological analysis with a series of immunohistochemical studies to further examine the effects of homozygous loss of *Tubb2a* or *Tubb2b*. The overall laminar organization was not disrupted in any deletion allele but we did note some subtle disruptions. NeuN is a marker of differentiated neurons and we note subtle differences in the staining as the NeuN cells seem to be more dispersed in the mutant cortex (Fig 4A–4E). CTIP2 marks layer V neurons and appears relatively unaltered in the deletion alleles (Fig 4F–4J, S5A–S5C Fig). We quantified the distribution of CTIP2-postive cells and did see subtle increases in the proportion of cells in the middle third of the cortex (Bin 2) at the expense of the dorsal third (Bin 3) consistent with a very mild migration defect of the CTIP2-positive neurons. This appears to be much less pronounced in the *Tubb2b* deletion mutant (S5 Fig, S4 Table). TBR1 is a marker of layer IV-VI neurons and, as with CTIP2, we observed a similar but more marked reduction in the number of cells in the middle third of the cortex (Bin 2) of the dorsomedial cortex. In the immediately adjacent parietal cortex, this migrational anomaly is again seen in the *Tubb2a*^*d3963/d3963*^ homozygotes but not other mutants (Fig 4K–4T, S5D–S5I Fig). Future studies will be needed to determine how significant and/or widespread these subtle anatomical differences are between the *Tubb2a* alleles. *Tubb2b*^*d4185/d4185*^ mutants also show decreased TBR1-positive cells in the middle third of the dorsomedial cortex but not the parietal cortex (Fig 4N, 4O, 4S and 4T, S5 Fig) We also used Myelin Basic Protein (MBP) to highlight axon tracts and found reduced staining in the corpus callosum and pyramidal fibers of the *Tubb2a* homozygotes but not in *Tubb2b* homozygotes (Fig 4U-II). We conclude from these data that neither *Tubb2a*, nor *Tubb2b*, are uniquely required for survival in the mouse but do result in subtle cortical malformations. However, neither loss of *Tubb2a* nor *Tubb2b* results in the severe cortical malformations seen in the most common tubulinopathy patients.

### Deletion of either *Tubb2a* or *Tubb2b* leads to no change in total β-tubulin protein levels

Tubulin expression is well known to be auto-regulated in mammalian cells [43, 44]. The production of the initial portion of the tubulin polypeptide chain serves as a signal to the cell to reduce the stability of remaining tubulin mRNA [45–51]. This mechanism has implications for the ability of a cell to compensate for deletion of a single tubulin locus. We therefore considered if the amount of tubulin protein in the P0 brain was altered upon deletion of *Tubb2a* or *Tubb2b*. We are unable to definitively demonstrate the loss of TUBB2A or TUBB2B protein in our mutants as the amino acid sequences are identical at all but two amino acids precluding any antibody-based analyses such as Western immunoblotting (S6 and S7 Figs). RNA analysis of gene expression is similarly challenging due to sequence similarity (S8 and S9 Figs). Some RT-PCR primers have been used previously, but our analysis suggests these might amplify other genomic elements such as poorly annotated pseudogenes (S10 Fig). Expression of *Tubb2b* mRNA in mouse has been reported [26], but a further analysis of the published *in situ* probe shows high sequence similarity to the *Tubb2a* sequence, suggesting that this reported expression may, perhaps, be a combination of *Tubb2a* and *Tubb2b* (S11 Fig).

**Fig 5:**
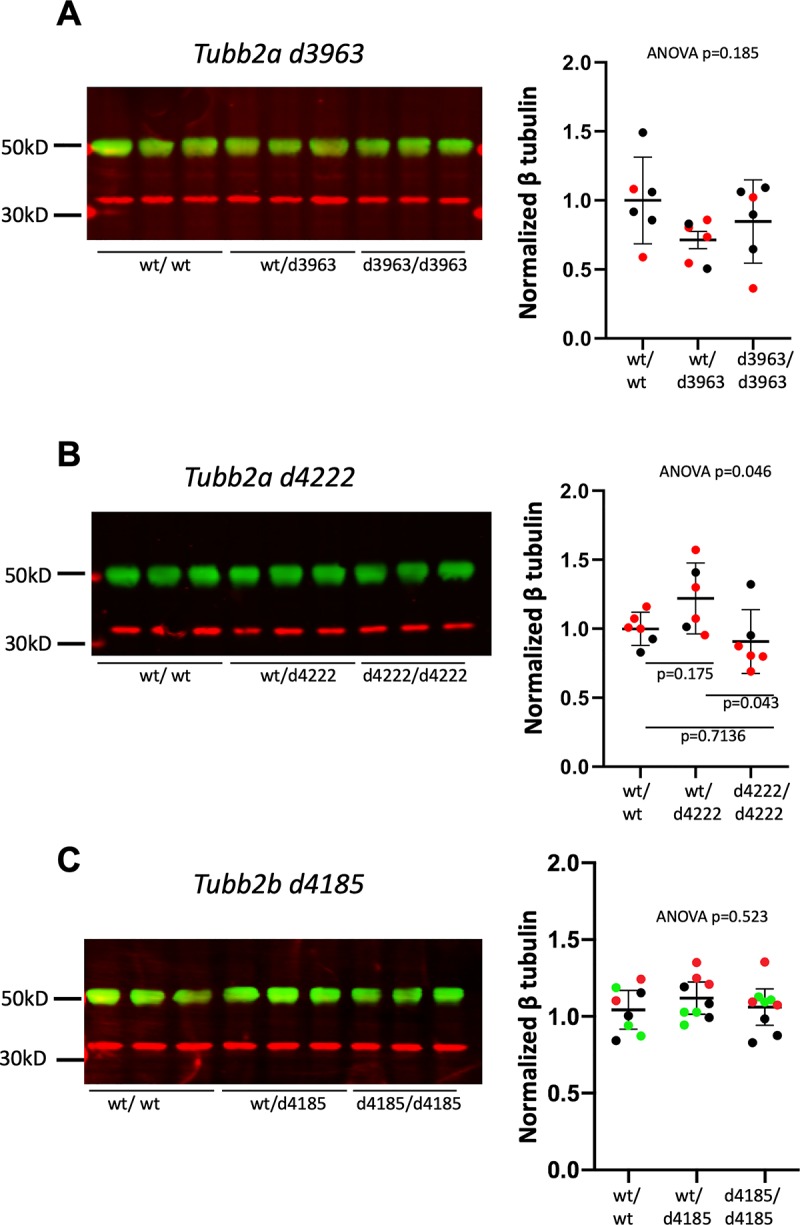
Total β-tubulin protein levels in *Tubb2a* and *Tubb2b* mutants. Western immunoblotting with an antibody recognizing multiple isoforms of **β**-tubulin for P0 brain lysates from (A) *Tubb2a*^*d3963*^ (B) *Tubb2a*^*d4222*^and (C) *Tubb2b*^*d4185*^ mice. Representative immunoblots for each allele are shown. Quantifications are of multiple experiments normalized to wild-type littermate controls. (A,B) Colors indicate littermates. n = 3 animals of each genotype, each set was run in duplicate. (C) Colors indicate replicate experiments from one set of littermates (Green bands indicate tubulin and red indicate GAPDH loading control for all immunoblotting experiments).

However, we were able to address the question more broadly with an antibody that recognizes multiple **β**-tubulins (Fig 5, S12 Fig, S5 Table). Immunoblotting with this antibody did not indicate any biologically significant reductions in the deletion mutants. (We did note an apparent increase in total **β**-tubulin levels in *Tubb2a*^*d4222/wt*^ animals, which become magnified in comparison to a very small decrease in *Tubb2a*^*d4222/d4222*^ total **β**-tubulin when each is compared to wild-type, Fig 5B). We note that the high levels of tubulin in comparison to other specific protein in the cell makes precise quantification difficult with the low levels of total protein analyzed in these experiments (see below discussion in *Tuba1a* mutants). This suggests that loss of TUBB2A or TUBB2B is compensated by other **β**-tubulins expressed in the developing brain. This may explain the relatively mild phenotypes we report here in these homozygous deletion mutants.

### *Tuba1a* is required for survival and brain development

In stark contrast to our findings with *Tubb2a and Tubb2b*, *Tuba1a* is absolutely required for survival to weaning as we did not recover any homozygotes for either *Tuba1a* deletion allele (n = 177 total; Table 2). We do note a small reduction in heterozygote survival among the surviving animals. To begin to understand the reason for the lethality and to assess brain development in the homozygotes, we collected embryos at late organogenesis stages. A combined analysis of both *Tuba1a* deletion alleles in embryos from embryonic day (E)14.5-E18.5 did not reveal a significant loss of homozygous embryos during embryonic stages (Table 2).

**Table 2:** Survival of *Tuba1a* deletion mutants*.

	wild-type	heterozygous	homozygous	total	p-value
**Survival at weaning**					
*Tuba1a*^*d4262*^	36	39	0	75	2.95e^-08^
*Tuba1a*^*d4304*^	38	64	0	102	9.39e^-26^
*Tuba1a* combined	74	103	0	177	3.4e^-15^
**Embryonic survival**					
*Tuba1a*^*d4262*^ x *Tuba1a*^*d4262*^	7	18	11	36	0.64
*Tuba1a*^*d4304*^ x *Tuba1a*^*d4304*^	40	105	51	196	0.17
*Tuba1a* combined	47	123	62	232	0.18

* All data here are from heterozygous intercrosses

**Fig 6:**
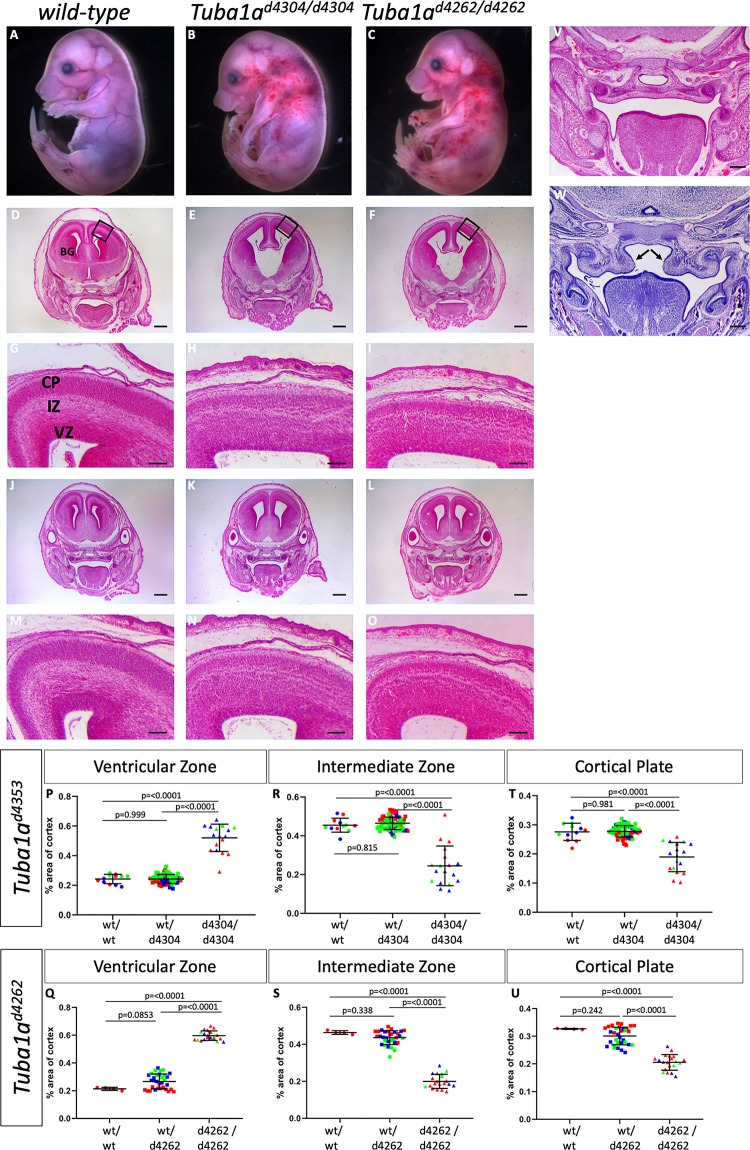
Loss of *Tuba1a* leads to perinatal lethality and cortical malformations. Embryos homozygous for either of two *Tuba1a* deletions (*Tuba1a*^*d4304*^ B,E,H,K,N; *Tuba1a*^*d4262*^ C,F,I,L,O) at E16.5 have significant gross morphological phenotypes when compared to wild-type (A,D,G,J,M). Apparent hemorraghing in the cervical region is often noted in mutants (B,C) making them distinguishable upon initial embryo harvesting. Histological analysis shows enlarged ventricles with hypoplastic basal ganglia (BG) and disruptions to the ventricular zone (VZ), intermediate zone (IZ) and cortical plate (CP) in both mutants. Black boxes in D-F show highlighted areas in G-I, respectively. (Sections in D-I are in a more anterior portion of the embryo than J-O to accentuate different aspects of the cortical malformations). Quantification of the width of the VZ (P,Q), IZ (R,S) and CP (T,U) confirms an increased width of the VZ but reductions in the IZ and CP for both alleles. (n = 3 wild-type, 27 heterozygous and 6 mutant embryos for *Tuba1a* ^*d4304*^ and 1 wild-type, 12 heterozygous, and 5 mutant embryos for *Tuba1a* ^d4262^). Some mutants show cleft palate (W) resulting from a failure of the hypoplastic palatal shelves (arrowheads) to elevate and fuse as seen in wild-type embryos (V). Scale bars indicate 400 μm in D-F,J-L; 100 μm in G-I,M-O; and 200 μm in V,W.

*Tuba1a*^*d4304/d4304*^ and *Tuba1a*^*d4262/d4262*^ animals had very obvious and consistent phenotypes. We noticed thoracic curvature and thoracic edema with significant hemorrhaging in the cervical region extending towards the forelimbs (Fig 6A–6C). Histological analysis of the developing forebrain revealed a striking series of forebrain malformation phenotypes. Sections from both posterior and anterior regions of the forebrain highlight enlarged lateral and third ventricles, reduced basal ganglia, as well as a widened base of the third ventricle in posterior brain regions (Fig 6D–6O). Examination of cortical morphology showed a 115–179% increase in the width of the ventricular zone at E16.5, a 50% reduction in the intermediate zone, and an approximate 30% reduction in width of the cortical plate as compared to wild-type (Fig 6P–6U; S6 Table). We also noted a number of the homozygous mutants had cleft palate (Fig 6W). This was incompletely penetrant and seen in 3/11 *Tuba1a*^*d4304/d4304*^ embryos (27%) and 2/16 *Tuba1a*^*d4262/d4262*^ (12.5%) embryos. We noted cleft palate in only one out of 52 heterozygous embryos analyzed, but we suspect this was likely due to delayed development in that particular embryo based on morphological staging. We conclude that *Tuba1a* is required for survival and that loss of *Tuba1a* leads to major cortical malformations with similarities to what is seen in the human tubulinopathy patients.

### Altered patterns of neurogenesis upon loss of *Tuba1a*

**Fig 7:**
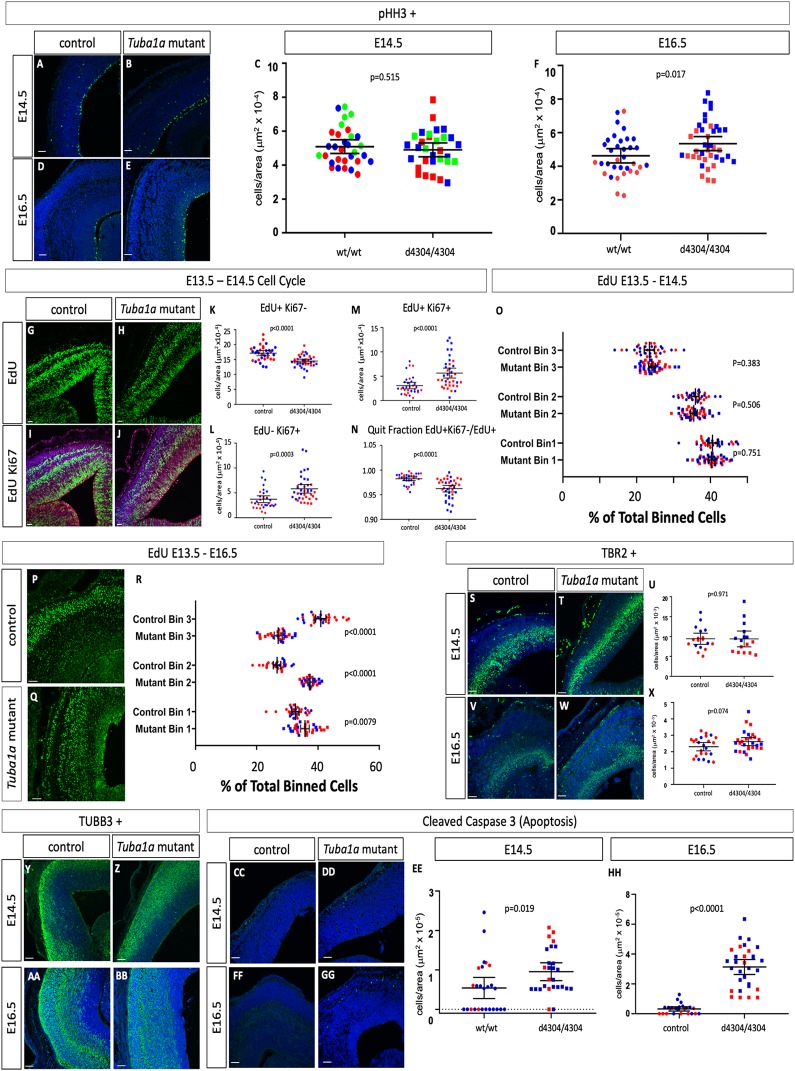
Loss of *Tuba1a* leads to altered patterns of neurogenesis and migration. (A-F) Immunohistochemistry for pHH3 does not indicate a difference between wild-type embryos and homozygous *Tuba1a* mutants at E14.5, but an increase in mutants at E16.5 (n = 3 wild-type and mutant embryos at E14.5 and n = 4 for both at E16.5). (G-R) EdU pulse chase analysis. EdU cells were labeled *in vivo* at E13.5 and embryos were fixed and immunostained for Ki67 and EdU at E14.5 (G-O) or E16.5 (P-R). In comparison to wild-type cells at E14.5 (n = 4 wild-type and mutant embryos), the number of *Tuba1a* mutant total EdU+ cells are reduced (K), EdU-Ki67+ cells are increased (L), EdU+Ki67+ cells are increased (M) and the quit fraction (EdU+Ki67-/EdU+) is decreased (N). (O) The position of EdU+ cells across the dorsal-ventral axis cortex during radial migration shows no difference in tissue distribution between wild-type and mutant. (P-R) Embryos labeled from E13.5-E16.5 show reduced radial migration (n = 3 wild-type and mutant embryos). (S-X) The number of TBR2-positive intermediate progenitors at E14.5 (S-U)does not appear to differ between wild-type and *Tuba1a* mutants but is increased at E16.5 (V-X; n = 4 wild-type and mutant embryos). (Y-BB) TuJI-positive differentiated neurons appear to be dispersed over a broader portion of the cortex in *Tuba1a* mutants at E14.5 and E16.5 (n = 3 control and mutant embryos for both). (CC-HH) Cleaved caspase 3 immunoreactivity as a marker for apoptosis is increased at E14.5 and E16.5 (n = 3 control and mutant embryos for both). Scale bars indicate 100 μm.

We have performed an initial molecular analysis of neurogenesis in the *Tuba1a*^*d4304/d4304*^ embryos at E14.5 and E16.5 as compared to unaffected wild-type or heterozygous littermates (Fig 7, S7 Table). We first measured proliferation rates with immunohistochemistry for phosphorylated histone H3 (pHH3) to mark actively dividing cells in M-phase and found no significant difference between wild-type and controls at E14.5 (Fig 7A–7C) but a slight 15.7% increase at E16.5 (Fig 7D–7F). We further examined neurogenesis with an EdU-Ki67 pulse chase assay in which we treated pregnant dams with EdU at E13.5 to label dividing cells, sacrificed at E14.5 and used an antibody against Ki67 to mark cells in G1, S, and G2 phases of the cell cycle at the time of sacrifice (Fig 7G–7N). This analysis indicated a 15.6% decrease in total EdU-positive, Ki67-negative cells in the mutant cortex at E14.5 (Fig 7K) suggesting that fewer overall cells were produced in the 24-hour period between EdU administration and sacrifice. Cells which are Ki67-positive and EdU-negative are actively dividing at E14.5 and were increased 56.7% (Fig 7L). Note that Ki67 marks a broader sample of the cell cycle than pHH3 which may explain why this is increased while the number of pHH3-positive cells did not appear different. Cells positive for both EdU and Ki67 were progenitors dividing at E13.5 which re-entered cell cycle and these are increased in mutants by 84.3% (Fig 7M). The quit fraction is the proportion of cells leaving the cell cycle over total labeled cells (EdU+Ki67-/EdU+) and is 2% reduced in mutants (Fig 7N). We conclude from these data that the enlarged ventricular zone we see in the homozygous *Tuba1a* deletion mutants is due to disrupted neuroprogenitor cell division kinetics in which some cells at E14.5 are preferentially re-entering cell cycle rather than terminally dividing.

EdU treatment also allowed us to assess the fate of cells 24 and 72 hours after division. We divided the cortex in three equal parts and quantified the percentage of EdU cells in each third of the cortex along the dorsal-ventral axis to query their developmental trajectory as they migrate into the cortical plate. We did not see a significant change in these parameters at E14.5 (Fig 7O). However, when we administered EdU at E13.5 and sacrificed at E16.5, we noted dramatic increases in cells in the middle third of the cortex at the expense of the outer third, consistent with a failure of neurons born at E13.5 to fully survive, differentiate and/or migrate towards the pial surface (Fig 7P–7R).

We also measured the levels of TBR2-positive intermediate progenitors and observed no changes at E14.5 (Fig 7S–7U) but a slight increase at E16.5 (Fig 7V–7X; 13.5% increase). TuJI immunoreactivity of maturing neurons indicated a slight reduction in levels of differentiation and a broader dispersal of TuJI-positive cells throughout the cortical tissue in homozygous mutants at both E14.5 and E16.5 (Fig 7Y-BB). We also examined levels of apoptotic cell death with immunohistochemistry for cleaved caspase 3 and found increases at both E14.5 and E16.5 (elevated 75.9% and 866% respectively, Fig 7CC-HH). Taken together, all of these data lead us to conclude changes in neurogenesis, migration and cell death contribute to the cortical phenotypes. The ultimate cause of the lethality of these cells, and the mouse mutant embryos as a whole, remains to be determined.

### A novel ENU allele of *Tuba1a*

**Fig 8:**
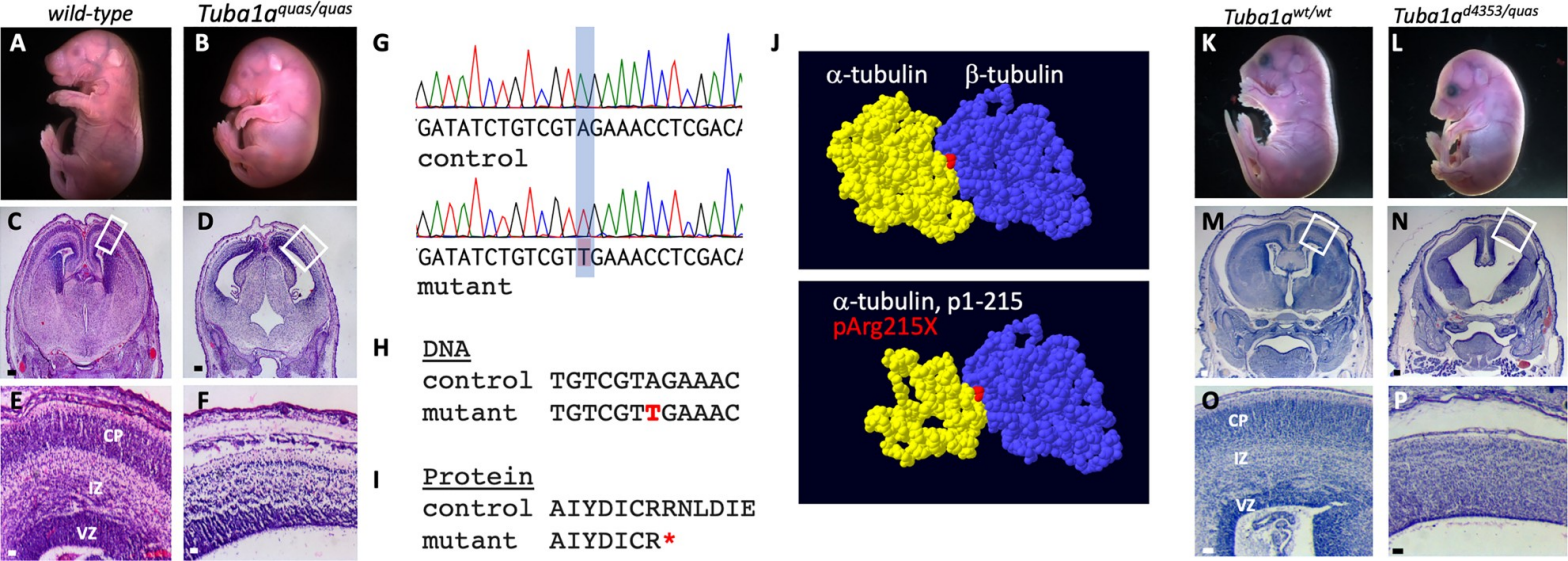
The *quasimodo* mutation is an allele of *Tuba1a*. (A-F) Homozygous *quasimodo* mutants are distinguishable at E18.5 by the systemic edema (B). Histological analysis (E17.5) shows enlargement of the third ventricle (D) and reduced cortical tissue (F). (G) Sanger sequencing confirms homozygosity for a candidate SNP in a conserved DNA (H) and protein (I) sequence (SNP and coding change shown in red, red asterisk indicates premature stop codon). (J) Structure of the wild-type α/β tubulin dimer is shown above and the remaining α-tubulin structure is shown below (red = non-hydrolyzable GTP at the monomer interface). (K-P) Complementation analysis at E17.5 with the *Tuba1a*^*d4304*^ is consistent with *quasimodo* being an allele of *Tuba1a* as *Tuba1a*^*d4304/quas*^ mutants have gross (L) and histological (N,P) phenotypes similar to the other *Tuba1a* homozygous phenotypes. The failure of alleles to complement indicates both mutations are in *Tuba1a*. Boxes in C,D,M,N show areas enlarged in E,F,O,P, respectively. Scale bars indicate 200 μm in C,D,M,N and 50 μm in E,F,O,P.

In a separate experiment utilizing an ENU mutagenesis forward genetic screen to ascertain new alleles important for cortical development, we identified an additional mutant with similar brain phenotypes to those seen in the *Tuba1a* deletion mice. The *quasimodo* mutants were first identified by an abnormal curvature of the thoracic region seen in many of these mutants (Fig 8B). Histological analysis of mutants showed a significant brain malformation with lateral ventriculomegaly, reduced cortical tissue, and enlarged third ventricle (Fig 8C–8F). Whole exome sequencing of three homozygous mutants identified a large number of homozygous variants as predicted. Filtering for SNPs which were shared by all three mutants, not present in dbSNP, predicted to have a high/moderate effect on the protein (e.g., missense variants at conserved residues, premature stop codons, etc.), and having only one variant in the gene (i.e., not a highly polymorphic sequence) left a total of ten variants. A review of literature to identify known consequences for loss of function in these genes or known roles of these genes did not yield any compelling candidates (Table 3).

**Table 3:** Mouse exome variant analysis in *Tuba1a*^*quas*^ homozygous mutants.

Exome Variants	# of variants
total	199,085
Homozygous in all three animals	982
not in dbSNP	575
SNP	324
High/ moderate predicted effect	43
Single variant in gene (*Rapgef4*, *Ckap5*, *Gon4l*, *Cope1*, *Fat2*, *Med1*, *Nrxn3*, *Glac*, *Ptpn21*, *Dnahc11*)	10

We then performed a parallel analysis for all heterozygous missense variants shared by the three sequenced mutants with the alternative hypothesis that the *quasimodo* phenotype may be an incompletely penetrant heterozygous variant. One variant from this analysis was a nonsense mutation in *Tuba1a* creating a premature stop codon at amino acid 215 (p.R215*). We noted that the sequence in this region of *Tuba1a*, *Tuba1b* and *Tuba1c* is highly similar and carefully analyzed the exome .bam files. Virtually all of the reads for this region of genomic sequence were aligned to the *Tuba1a* gene and virtually no reads were mapped to *Tuba1b* or *Tuba1c*. Given the phenotypic similarity to the *Tuba1a* deletion, we hypothesized the similarity among these genes may have confounded the automated sequence alignment and pursued Sanger sequencing with intronic primers that would specifically amplify *Tuba1a*. This analysis showed that the *quasimodo* mutants were all in fact homozygous for the *Tuba1a* variant creating the nonsense mutation (Fig 8G–8I). This mutation is predicted to produce a protein missing approximately half of the amino acid sequence, if that protein is even stable in the cellular context (Fig 8J). The mutant protein would be missing significant portions of the polypeptide which would normally interface with the adjacent β-tubulin monomer.

In order to confirm that the *Tuba1a* R215* variant is actually the causative variant in the *quasimodo* mutants, we performed a complementation test with the *Tuba1a*^*d4304*^ deletion allele. We crossed *Tuba1a*^*quas/wt*^ with *Tuba1a*^*d4304/wt*^ animals and in three litters (22 live pups), we recovered no *Tuba1a*^*d4304/quas*^ live animals at weaning (Table 4). We further explored this hypothesis with embryonic dissections and recovered two *Tuba1a*^*d4304/quas*^ embryos at E17.5. These mutants exhibited the exaggerated curvature of the thoracic region, as previously seen in *Tuba1a*^*quas/quas*^ mice and *Tuba1a*^*d4304/d4304*^ mice (Figs 6B and 8L). Histological analysis showed ventriculomegaly with disrupted cortical architecture including a loss of the intermediate zone, hypoplastic basal ganglia, and cleft palate in both mutants (Fig 8M–8P). The similarity of these phenotypes to the *Tuba1a*^*d4304/d4304*^ and *Tuba1a*^*quas/quas*^ mutants suggests that the *Tuba1a* R215* variant found in *quasimodo* mutant is indeed the causal variant. Moreover, this would support the above conclusion that loss of *Tuba1a* function results in significant cortical malformations.

**Table 4:** *Quasimodo* is an allele of *Tuba1a*.

	wild-type	*quas/*wt	*d4304*/wt	*d4304*/quas	total	p
*Tuba1a*^*d4304/wt*^ x *Tuba1a*^*quas/wt*^ weaning	7	6	9	0	22	0.04
*Tuba1a*^*d4304/wt*^ x *Tuba1a*^*quas/wt*^ embryos	6	9	8	2	25	0.42

### Proliferation and radial migration phenotypes in *Tuba1a*^*quas*^ mutants

**Fig 9:**
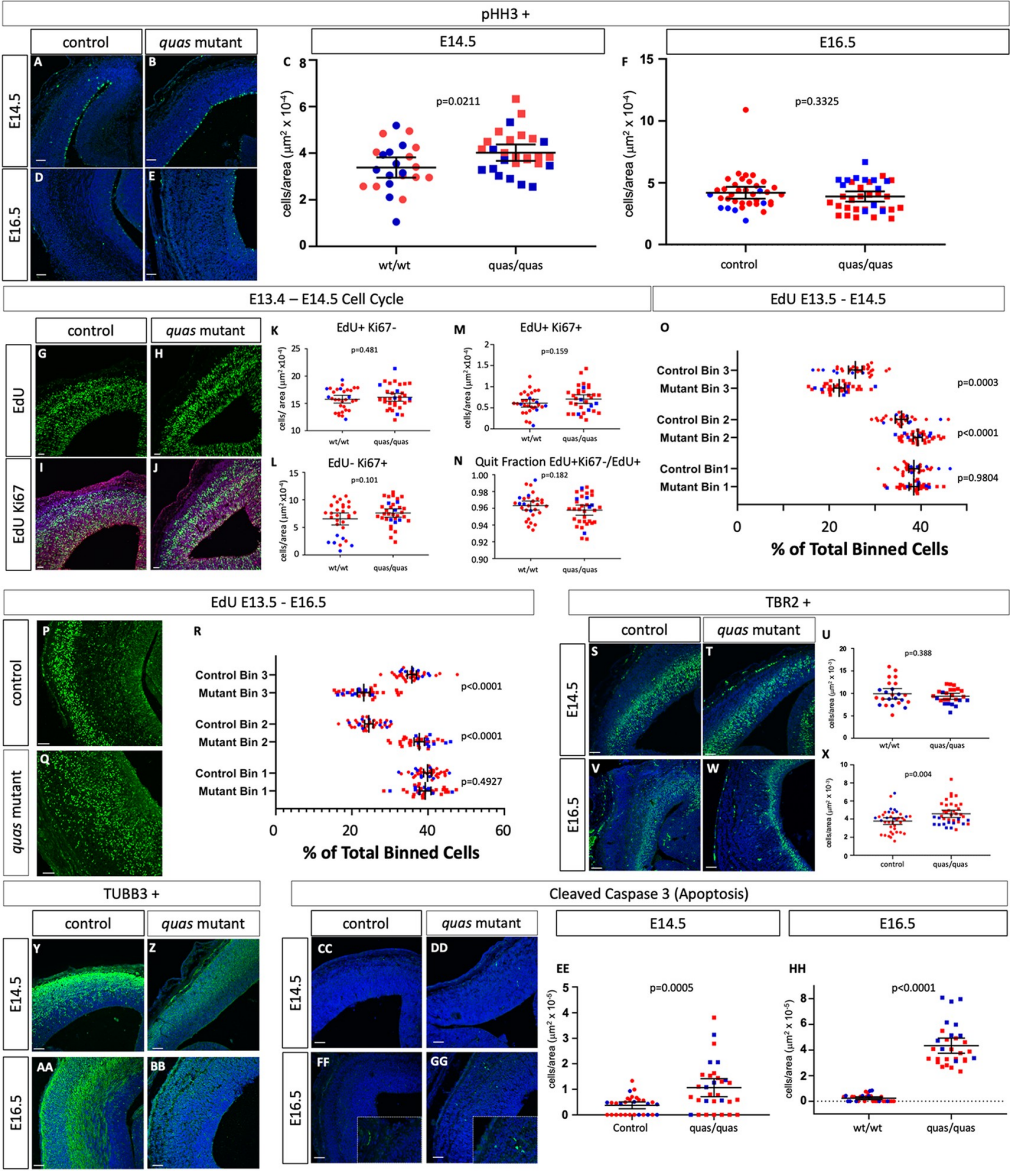
Altered proliferation, radial migration and apoptosis in *Tuba1a*^*quas*^ mutants. (A-F) Immunohistochemistry for pHH3 indicates a difference between wild-type embryos and homozygous *Tuba1a*^*quas*^ mutants at E14.5, but not E16.5 (n = 3 wild-type and mutant embryos for each). (G-R) EdU pulse chase analysis. EdU cells were labeled *in vivo* at E13.5 and embryos were fixed and immunostained for Ki67 and EdU at E14.5 (G-O) or E16.5 (P-R). In comparison to wild-type cells at E14.5 (n = 4 wild-type and mutant embryos), none of the cell cycle parameters appear to differ (G-N). (O) The position of EdU+ cells across the dorsal-ventral axis cortex during radial migration shows altered migration patterns. (P-R) Embryos labeled from E13.5-E16.5 also show reduced radial migration (n = 4 wild-type and mutant embryos). (S-X) The number of TBR2-positive intermediate progenitors at E14.5 (S-U) does not appear to differ between wild-type and *Tuba1a*^*quas*^ mutants, but is increased at E16.5 (V-X; n = 4 wild-type and mutant embryos). (Y-BB) TuJI-positive differentiated neurons appear to be dispersed over a broader portion of the cortex in *Tuba1a*^*quas*^ mutants at E14.5 and E16.5 (n = 3 control and mutant embryos for both). (CC-HH) Apoptosis is increased at E14.5 and E16.5 (n = 3 control and mutant embryos for both). Scale bars indicate 100 μm.

We performed a molecular analysis of neurogenesis in the *Tuba1a*^*quas*^ mutants as we did for the *Tuba1a* deletion mutant (Fig 9, S7 Table). Surprisingly, although the terminal phenotypes are similar, the data suggest different underlying mechanisms. Proliferation in *Tuba1a*^*quas*^ mutants was increased by 18.9% as measured by pHH3 immunoreactivity at E14.5 (Fig 9A–9C) but decreased 7.2% at E16.5 (Fig 9D–9F). In contrast to the *Tuba1a* deletions however, the EdU-Ki67 pulse chase experiments did not reveal any robust differences in patterns of neurogenesis as we saw in the *Tuba1a* deletion mutant (Fig 9G–9N). The EdU labeling to analyze cell migration highlighted a significant reduction in cells labeled at E13.5 which migrated to the upper third of the cortex at E14.5 and a corresponding increase in number of cells in the middle third (Fig 9O). The same patterns are seen at E16.5 with more marked changes in cellular distributions (Fig 9P–9R). This data is most consistent with a decreased radial migration by cells born on E13.5, similar to what we observed in the *Tuba1a* deletion mutants.

We also saw no marked changes in the numbers of TBR2-positive intermediate progenitors in the *Tuba1a*^*quas*^ mutants at E14.5 (Fig 9S–9U) but a slight 21.5% increase at E16.5 (Fig 9V–9X). TuJI immunoreactivity again showed an increased range of differentiated cells similar to the *Tuba1a* deletion mutants at both E14.5 and E16.5 (Fig 9Y-BB). Similar to the analysis of the *Tuba1a* deletion mutants, *Tuba1a*^*quas*^ mutants show elevated levels of apoptotic cell death which is readily apparent at E14.5 and E16.5 (elevated 185% and 1721% respectively, Fig 9CC-HH). We again conclude that the *Tuba1a*^*quas*^ phenotype is the result of disrupted neurogenesis, disrupted migration and a marked increase in cell death.

### Mutations at *Tuba1a* locus lead to changes in α-tubulin and β-tubulin protein levels

**Fig 10:**
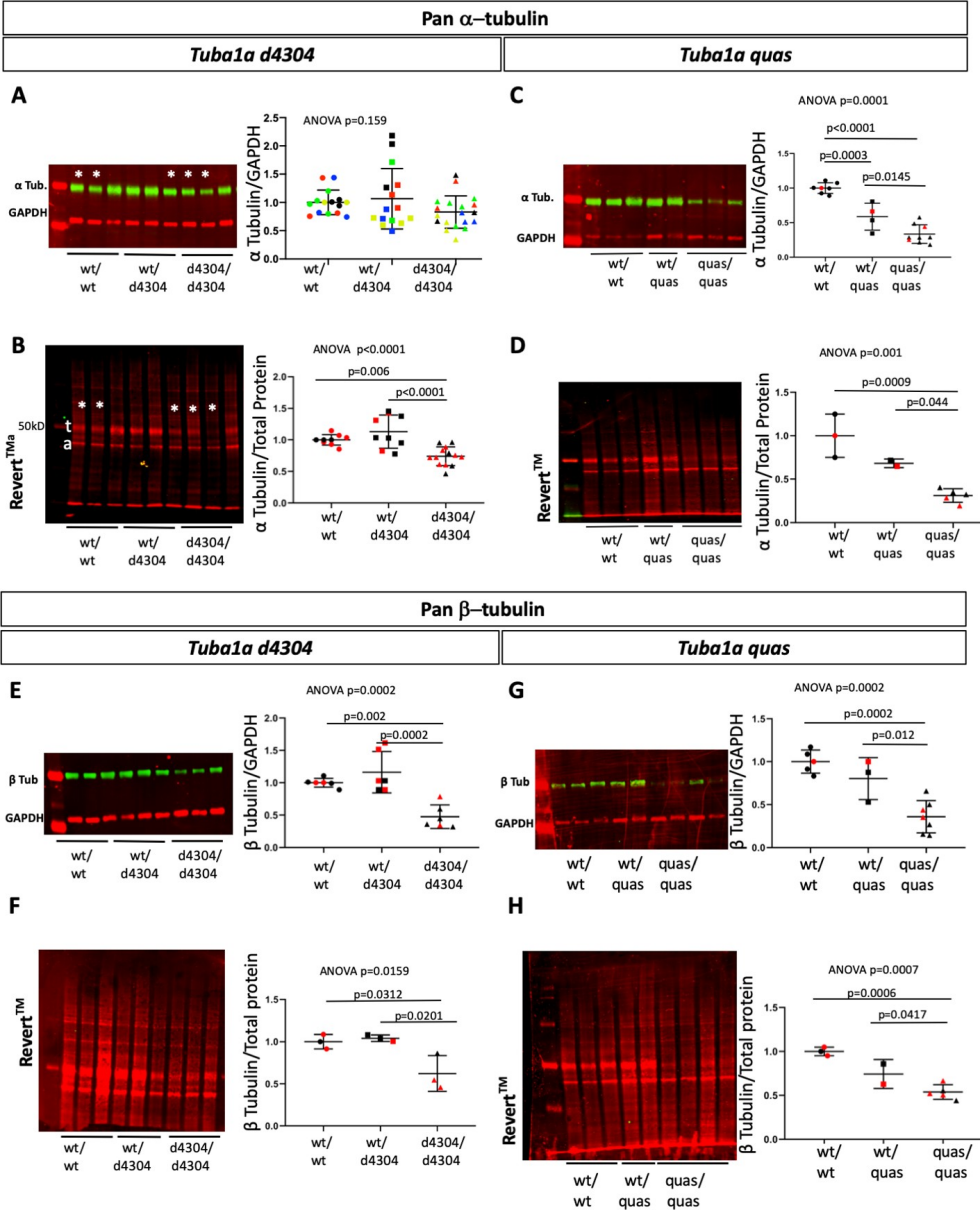
Total α-tubulin and β-tubulin protein levels in *Tuba1a* mutants. Immunoblotting for pan-α (A-D) and pan-β (E-H) tubulin in *Tuba1a*^*d4304*^ (A,B,E,F) and *Tuba1a* ^*quas*^ (C,D,G,H) wild-type, heterozygous and mutant embryos. Protein levels were quantified by comparison to GAPDH loading control (A,C,E,G) and REVERT total protein stain (B,D,F,H). (A) Colors indicate results from five different technical replicates. (B-H) Colors indicate littermates (technical replicates are shown for A-C,E,G). For *Tuba1a*^*d4304*^, n = 3 animals of each genotype, for *Tuba1a*^*quas*,^ wild-type n = 3, heterozygous n = 2, and mutant n = 5. (t = presumed tubulin protein, a = presumed actin protein).

Similar to the analysis we performed on the levels of β-tubulin protein in *Tubb2a* and *Tubb2b* mutants (Fig 5), we also analyzed total α-tubulin levels in the *Tuba1a* mutants described here. We used an antibody that shares similarity with multiple α-tubulin proteins at the N-terminal portion of the reported epitope (Fig S13). We find that total α-tubulin levels do not appear to be reduced in *Tuba1a*^*d4304/d4304*^ homozygous mutants when normalized to GAPDH levels within each litter matched set (Fig 10A). Even after five independent experiments, we could not convincingly demonstrate loss of α-tubulin even though matched samples (e.g., asterisks in Fig 10A are a set of littermates) often indicated a subtle decrease. We reasoned the very low level of protein input to our experiments may have challenged the limits of accurate and robust detection of the imaging system in embryonic brain lysate. We note GAPDH levels are extremely low at this stage of development. We ran a parallel analysis for total protein, rather than a single protein, as a loading control. REVERT total protein stain is not based on any particular protein epitope. When we analyzed REVERT staining of the transfer membrane we saw a striking decrease in total protein around the molecular weight of tubulin in mutant samples (Fig 10B). We reason this species is likely mostly tubulin protein. When we normalize the levels of total α-tubulin to total protein as measured by REVERT total protein stain, we do see a significant reduction in total α-tubulin in mutants as compared to both wild-type and *Tuba1a*^*d4304*^ heterozygous littermates (Fig 10B, S8 Table). We performed a similar analysis of total α-tubulin in *Tuba1a*^*quas*^ mutants and regardless of which tubulin normalization method used, we see a dramatic loss of more than half the α-tubulin in mutant tissue (Fig 10C and 10D).

The dramatic reduction in overall tubulin on the membranes stained for total protein (e.g., Fig 10B) led us to hypothesize that decreased levels of α-tubulin might have an effect on β-protein as well. This may be through an unknown mechanism to keep massive amounts of unpolymerized β-tubulin monomers from flooding the cell. To our surprise, we do indeed see a decrease in total β-tubulin upon loss of *Tuba1a* (Fig 10E and 10F) and in homozygous *Tuba1a*^*quas*^ mutants (Fig 10G and 10H). This regulation of tubulin monomers is a very intriguing question to be addressed further in future research. This will likely require work *in vitro* with tagged versions of the tubulins of interest.

## Discussion

We have for the first time demonstrated the consequences of the loss of three tubulin genes known to be important for normal mammalian cortical development: *Tuba1a*, *Tubb2a*, and *Tubb2b*. We also identified a new *Tuba1a* mouse allele with severe CNS phenotypes through an ENU mutagenesis experiment. These alleles contribute to a larger body of work indicating the importance of tubulin genes in cortical development and disease. These deletion alleles allow us to further explore the model that the human variants identified in *TUBA1A*, *TUBB2A* and *TUBB2B* are acting as dominant negative heterozygous mutations (e.g. [52]), but may not be truly essential for development. We find that *Tubb2a* and *Tubb2b* can be deleted from the mouse genome with no obvious effect on gross cortical development, animal survival, or overall health. *Tuba1a*, however, is quite different, as loss of *Tuba1a* is lethal in the mouse and leads to severe malformations of cortical development. Thus, other tubulin genes with high sequence similarity cannot compensate for *Tuba1a* but may be doing so for *Tubb2a* and *Tubb2b* (e.g., *Tubb3)*. Together, these data show that some tubulin genes are absolutely critical for mouse CNS development and organism survival, while others are less critical.

*Tubb2a* and *Tubb2b* are highly similar and immediately adjacent in the genomes of both mice and humans, suggesting that a recent duplication event in a common ancestor may have rendered these genes redundant for each other. This may explain why a single deletion of each does not lead to major CNS phenotypes. However, we do note more subtle cortical phenotypes including changes in cell number in the motor and somatosensory cortices and in disrupted cortical lamination consistent with more mild radial migration defects. We also note a striking reduction in MBP immuno-staining in the *Tubb2a* deletion homozygous mice. We suspect this is a result of reduced axon tracts rather than defective myelination. This phenotype and the possible behavioral consequences of the cortical disruptions should be interrogated rigorously in future studies. We acknowledge that mouse models of human cortical malformations may not be perfect due to species differences. However, we do not believe any such differences can explain the normal presentation of these mice. Indeed, we have previously shown structural and behavioral phenotypes in a mouse *Tubb2b* missense allele [42, 53].

While the *Tubb2a* and *Tubb2b* deletion mutants appear to have relatively subtle phenotypes, they each may be rescued from more global malformations through functional redundancy. This model can be tested with a double mutant analysis. The genes are sufficiently close to each other within the genome such that this experiment will require creation of an independent deletion or conditional allele in *cis* to one of the existing mutations.

Given these findings that homozygous deletions of *Tubb2a* or *Tubb2b* do not result in catastrophic malformations of cortical development, it is perhaps surprising that there are only two homozygous coding missense variants and no homozygous loss of function variants in both *TUBB2A* and *TUBB2B* combined in the gnomAD collection of over 140,000 healthy individuals as of this writing [54]. Given the phenotypes we now report, it may be that the homozygous missense mutations lead to neurological disease which has not yet been attributed to tubulin genes, but would prevent inclusion of the affected individuals in the gnomAD and ExAC databases. The sequence similarities may also confound mapping of genomic information.

The mice heterozygous for the *Tubb2a* or *Tubb2b* deletions do not show any biologically significant loss of total beta-protein (Fig 5, S5 Table). We hypothesize other tubulins are produced at higher levels to compensate for the loss of each single deletion (e.g., *Tubb3*). These findings are consistent with the known data on tubulin autoregulation (see above). The reduced mRNA levels from the deletions should lead to reduced polypeptide production and, thereby, less de-stabilization of the tubulin mRNA, ultimately keeping monomer levels stable.

The TUBA1A polypeptide sequence is highly similar to TUBA1B (99.6% identical) and TUBA1C (98.2%) and three genes coding for these similar proteins are also clustered in the genome. These features suggest they may also be able to compensate for each other similar to our hypothesis regarding *Tubb2a* and *Tubb2b*. In direct contrast to our findings with *Tubb2a* and *Tubb2b*, however, loss of *Tuba1a* is catastrophic for the mouse embryo. The individual requirements for *Tuba1b* and *Tuba1c* remain unexplored and we will address this hypothesis with future deletion alleles. Loss of *Tuba1a* leads to an increased ventricular zone at E16.5 and our initial data from E14.5 and E16.5 suggests this is from a dysregulation of neural progenitor proliferation and cell cycle exit. Ultimately, failures in radial migration and/or apoptosis contribute to the phenotypes we observed. We also report an independently derived ENU-induced mutation in *Tuba1a* which encodes a transcript with a premature stop codon approximately halfway through the protein coding sequence. These homozygous *Tuba1a*^*quas*^ mutants have similar cortical phenotypes to the deletion mutants, but apparently develop through different mechanisms as the cell cycle kinetics do not appear to be disrupted (Figs 7 vs. 9). However, we do also see evidence for abnormal radial migration and elevated cell death in these *Tuba1a*^*quas*^ mutants. The significant difference in molecular phenotypes is likely to be the effect of a deletion of the gene as compared to production of a truncated polypeptide that may perturb microtubule dynamics.

The analysis of total α-tubulin protein levels in these mutants raises some other interesting points. Deletion of *Tuba1a* leads to decreased overall α-tubulin levels but not as dramatically as the reduction in the *Tuba1a*^*quas*^ mutants. This is consistent with the autoregulation of tubulin and suggests that other isoforms of *Tuba1a* are upregulated in the deletion mutant. However, given the significant phenotypes in the *Tuba1a* mutant, these other isoforms are likely unable to carry out some currently unknown critical function of the TUBA1A protein. Note that the ENU variant protein in the *Tuba1a*^*quas*^ allele is likely to create a protein lacking the amino acids needed for the anti-α-tubulin antibody we used. However, if the first several amino acids are translated from this locus as a missense mutation rather than completely missing in a deletion, this is consistent with the model of tubulin autoregulation where this initial translation leads to destabilizing existing tubulin mRNA transcripts. Given the extreme phenotype of the *Tuba1a*^*quas*^ homozygous embryos, we hypothesize the truncated TUBA1A protein produced in these mutants is not sufficient for normal embryonic development and in fact may interfere with multiple processes of tubulin physiology.

It is not known why the paradigm of genetic compensation is so different for these two gene clusters (*Tuba1a/b/c* as compared to *Tubb2a/b*). Why does loss of one result in severe malformations of cortical development while loss of others does not compromise survival or gross brain development? Do cells express a mix of α and β tubulin monomers? Are there changes in specific gene expression over time, perhaps between neurogenesis stages and terminal differentiation? These questions remain largely unanswered although a *Tubb2b-eGFP* transgenic line suggests *Tubb2b* is expressed in both progenitors and postmitotic neurons [55]. A detailed expression analysis would be useful in generating models to explain these genetic findings. Unfortunately, this is currently quite challenging. No antibodies will distinguish between these proteins in an immunohistochemical analysis (S6 and S7 Figs). RNA expression studies are complicated by this homology as well (S8 and S9 Figs). RNA *in situ* hybridization probes against the untranslated regions may be able to distinguish between genes but some hypotheses about co-expression at the cellular level would be challenging with *in situ* hybridization-based approaches. RNA-Seq, both bulk sequencing and single cell sequencing, would potentially be useful in addressing these models at least in a preliminary way. However, our experience with the *quasimodo* mouse mutant exome sequencing suggests the current library preparations and/or sequence alignment algorithms are confounded by the sequence similarities between these genes, and these data sets should to be interpreted with extreme caution. We propose that a series of epitope-fusion protein alleles in the mouse would be a useful tool set to explore both expression of discrete tubulin genes and will serve as a platform for biochemical approaches to identify microtubule associated protein and other cellular interaction partners.

While the phenotypes we present here are striking malformations of cortical development, we do not yet completely know the cellular and molecular basis for these phenotypes. The histological changes in the ventricular zone, intermediate zone and cortical plate all suggest fundamental perturbations of neurogenesis and/or cellular migration. Detailed mechanistic studies are needed to determine how these processes are altered in the face of tubulin mutations. The human missense mutations are not extensively modeled in mice, with the exception of the *Tubb3*^*R262C*^ model of CFEOM [31]. A mouse model of some of the most common mutations in *Tuba1a* would be helpful in both understanding the underlying mechanism(s), but also as a platform for testing potential therapeutic intervention.

Human genetics studies have identified a series of tubulin genes as crucial building blocks of the brain and tubulins have long been appreciated as important components for neuronal cell biology. As our genomic tools and mouse modeling capabilities have advanced, we now have the exciting opportunity to return to some fundamental questions about the requirement(s) and role(s) of tubulin genes in mammalian development and disease.

## Materials & methods

### Ethics statement

This study was carried out in strict accordance with the recommendations in the Guide for the Care and Use of Laboratory Animals of the National Institutes of Health. Animals were housed at or below IACUC determined densities with AALAC-approved veterinary care and fed Autoclavable Mouse Breeder Diet 5021 (LabDiet, St. Louis, MO). The protocol was approved by the Institutional Animal Care and Use Committee of the Cincinnati Children’s Hospital Medical Center (protocol number IACUC2016-0068). All euthanasia (cervical dislocation followed by thoracotomy) and embryo harvests were performed after isoflurane sedation to minimize animal suffering and discomfort.

### Mouse allele generation

CRISPR guides flanking the tubulin genes of interest were evaluated using the Fusi (Benchling.com) and Moreno-Mateos (Crisprscan.org) algorithms. Potential guide RNA (gRNA) sequences were selected and ordered as complementary oligonucleotide pairs with BbsI over-hangs (S1 Table; IDT, Coralville, IA). These were ligated into the pSpCas9(BB)-2A-GFP (px458) vector and transfected into MK4 cells at low confluence using the Lipofectamine 2000 transfection reagent (Thermo Fisher Scientific, Massachusetts). Cells were harvested 48 h after transfection and genomic DNA was isolated and used with the Surveyor mutation detection kit (IDT) in order to test gRNA cutting efficiency. As a control, cutting efficiencies of potential guides were compared with that of a previously-published mTet2 gRNA. Cas9 and gRNAs were injected into C57BL/6N zygotes (Taconic) by the CCHMC Transgenic Core. Potential founders were validated with Sanger sequencing of tail DNA and subsequently maintained on a C57BL/6J (Jackson Labs) background. pSpCas9(BB)-2 A-GFP (PX458) was a gift from Feng Zhang (Addgene plasmid #48138).

### Animal husbandry

For embryo collections, noon of the day of vaginal plug detection was designated as E0.5. Embryos and brains were removed and imaged using a Zeiss Discovery.V8 stereo-microscope.

### ENU mutagenesis

ENU mutagenesis was performed as previously described [56, 57] on C57BL/6J males.

### Histological analysis

For adult histology, littermate animals underwent transcardial perfusion using cold heparinized phosphate buffered saline (PBS) and formalin (SIGMA) solution. Brains were dissected and fixed for 72 h in formalin at room temperature followed by immersion in 70% ethanol (for histology). Embryo samples were fixed in Bouin’s fixative solution, formalin or 4% paraformaldehyde (PFA). Samples were then paraffin embedded or sucrose-dehydrated and cryo-embedded, sectioned at 6μm for adult tissue and 10μm for embryonic tissue, and processed through hematoxylin and eosin (H&E) or Nissl staining. Sections were sealed using Cytoseal Mounting Medium (Thermo-Scientific). All adult histological samples shown are representative examples from at least 3 animals of each genotype with at least 2 slides analyzed per animal. Dot plots are used to precisely show how many measurements are made.

### Immunohistochemistry

Immunohistochemistry was done accorded to standard protocols. In brief, E14.5 and E16.5 embryos and P0 brains were fixed overnight in 4% paraformaldehyde, dehydrated in 30% sucrose, cryo-embedded and sectioned at a thickness of 10 μm. P0 sections were collected at a thickness of 12 μm. Images were collected from a sample set of at least 2 slides of at least 3–5 sections for each animal and 3 control and 3 mutant embryos for each genotype at E14.5 and E16.5. P0 immunohistochemistry done on at least two animals per genotype, with 4 slides from each animal with 3–5 sections per slide. Sections for pHH3, TuJ1, NeuN, CTIP2, MBP, TBR1 were placed in boiling citrate buffer (sodium citrate 1M, citric acid 1M pH 6.0) and allowed to cool on bench top for 40 minutes. Sections for TBR2 were boiled for 4 minutes in antigen unmasking solution (Vector Labs, Burlingame, CA). Slides were blocked in 4% Normal Goat Serum in PBST for 30 minutes and then incubated overnight at 4 ^o^C in primary antibody. Secondary antibodies were added for 1 hour at room temperature. Slides were then washed in 1X PBS and DAPI was added for 15 minutes, after which slides were sealed with ProLong Gold (Invitrogen) and imaged on a Nikon Eclipse Ti or Nikon C2 confocal microscope. Primary antibodies used were pHH3 (1:500, SIGMA), TuJ1 (1:500, SIGMA), TBR2 (1:200, Abcam ab23345), TBR1 (1:500, Abcam ab31940), CTIP2 (1:1000, Abcam ab28448), and NeuN (1:1000, Millipore clone A60). Secondary antibodies were goat anti-rabbit (1:500 Invitrogen Alexa Fluor 488) for pHH3, TBR2, TBR1, and CTIP2, and goat anti-mouse (1:500 Invitrogen Alex Fluor 488) for TuJ1 and NeuN.

MBP immunohistochemistry staining was done with 3,3′-Diaminobenzidine (DAB) labeling. 5μm paraffin sections were de-paraffinized, rehydrated in graded ethanol, and incubated with chicken anti-MBP (1:500, Aves MBP) for 2 hours at room temperature, rinsed, and incubated in biotinylated goat-anti chicken secondary antibody (1:500; Vector Laboratories, Burlingame, CA, USA) for 2 hours at room temperature. Sections were then washed in PBS and incubated in avidin-biotin-complex (ABC) solution (Vector Laboratories) for 1 hour at room temperature. Sections were then washed and visualized using DAB for 5 min followed by a PBS wash, ethanol dehydration, xylene clearance, and mounted with Cytoseal (Thermo). Images were obtained with a Zeiss Discovery.V8 Stereoscope. From each genotype, 2 animals were included. From each of these animals, 2 slides each with 3–6 sections were stained. Sample sizes for quantifications are indicated in each relevant data plot. For P0 and adult histological analysis, areas of higher magnification analysis are shown (S3 Fig). Established anatomical landmarks were used to define cortical areas.

Quantification of pHH3-positive cells was done on Imaris Image Analysis software (Bitplane, South Windsor, CT) by manually drawing a surface around the Ventricular Zone and calculating the number of positive cells. Quantification of TBR2 and CC3 was done using Nikon Elements software by manually drawing a Region of Interest (ROI) around the entire cortex and calculating immuno-positive cells with a bright spot detection function.

“Binning” data for CTIP2 and TBR1-positive cells were acquired with Nikon Elements software after drawing an ROI that extended from the VZ to the pial surface. The length of this ROI is divided by three. The three identical squares were drawn to represent the three bins. Bright spot detection tool was employed to determine the amount of immune-positive cells in each bin. GraphPad Prism was used to plot the data and perform statistical analysis.

### EdU/Ki67 staining and quantification

EdU (Invitrogen) was injected at a concentration of 20mg/kg interperitoneally into E13.5 pregnant females. Embryos were harvested 24 or 72 hours later and stained as above. Ki67 primary antibody (1:200 Abcam ab15580) was added overnight at 4°C. A goat anti-rabbit secondary antibody (1:500 Invitrogen Alexa-Fluor 594) was added for 1 hour at room temperature. Slides were washed twice with 1X PBS and once with 3% BSA in PBS. EdU was labeled using the Click-iT EdU Alexa Fluor 488 Imaging Kit (Invitrogen).

Quantification was done using Imaris software package. A surface was manually created that contained the cortex from the VZ to the CP and the enclosed area was quantified. EdU positive cells and Ki67 positive cells were then identified using the Imaris “Spots” function. Cells both EdU and Ki67 positive were quantified using the “Colocalize Spots” function. Each positive cell count was normalized to the area of the section. Binning data for EdU positive cells was performed with Nikon Elements. Three identical rectangular Region of Interest bins were made for each section by dividing the width of the cortex into three equal parts. The number of cells in each bin was calculated using bright spot detection. The percentage of cells in each bin was used to determine any differences.

### Western immunoblotting

Whole cell lysate from P0/P1 brains were extracted with RIPA buffer (Thermo Scientific) with a protease and phosphatase inhibitor cocktail (Calbiochem) added immediately before cell lysis. A standard fluorescent western blotting protocol was followed. 5 μg of protein lysates were electrophoresed in 4–12% gradient Tris glycine PAGE gel (Thermo Fisher Scientific) and 1X Tris -Glycine SDS running buffer (Bio-Rad) and then transferred into PVDF membrane using Tris-Glycine transfer buffer with 20% methanol. Proteins were blocked in Odyssey blocking buffer (Licor Bioscience). Antibodies used were mouse anti TUBB3 (Tuj1, 1:400, SIGMA T8660), mouse anti-pan-β-tubulin (1:1000, Millipore 05–661), anti-pan-α-tubulin (1:500, Sigma T6199) and rabbit anti-actin (1:3000, SIGMA A2066) and were incubated with membrane overnight. Species specific secondary antibodies were applied to the membrane after stringent PBS washes of the primary antibodies. Odyssey CLx Imaging System were used to detect the bands of expression and Image Studio Lite software was used to analyze the band intensity. In each western experiment, GAPDH served as loading control and intensity of tubulin was normalized against GAPDH band intensity. For lysate from embryonic brains, we used a second loading/ normalization method by normalizing against total protein stain (REVERT from Licor Bioscience using manufacturer protocols).

### Statistical analysis

Analyses and data plots were generated with Prism8 (GraphPad) and plots show the mean +/- the 95% confidence interval. An ANOVA was performed for experiments with more than two comparisons. If an ANOVA p-value was ≤ 0.05, a Tukey’s multiple comparison test was performed to analyze which group(s) within the experiment were different from each other. Experiments with two sets of data were analyzed with a student’s t-test. The p-values for these experiments are shown in the relevant figure and/or supplemental data set. We report the statistical test values directly to facilitate readership and largely refrain from assigning labels of “significance” on our own.

All data presented here are available to the community in accordance with best practices for data sharing.

## Supporting information

S1 Fig **Additional CRISPR-Cas9 deletions of *Tubb2a* (A,C) and *Tuba1a* (B,D)**.
(TIF)

S2 Fig **Extended PCR analysis of *Tubb2a (A)*, *Tubb2b (B)*, *and Tuba1a (C)* deletion alleles**.
(PDF)

S3 Fig Approximate location of detailed images shown in Figs 2–4. (TIF)

S4 Fig Extended histological analysis of *Tubb2a* and *Tubb2b* homozygous mice. High magnification images with Hematoxylin and Eosin staining as shown for these alleles in Fig 2 for wild-type (A,E,I), *Tubb2a*^*d3964*^ (B,F,J), *Tubb2a*^*d4222*^ (C,G,K), and *Tubb2b*^*d4185*^ (D,H,L) homozygous mice.
(TIF)

S5 Fig **Quantification of the CTIP2 (A-C) and TBR1 (D-I) in the *Tubb2a* (A,B,D,E,G,H) and *Tubb2b* (C,F,I) deletion mutants**.
(TIF)

S6 Fig CLUSTAL 2.1 multiple sequence alignment of TUBB2A and TUBB2B protein sequence. (DOCX)

S7 Fig CLUSTAL 2.1 multiple sequence alignment of TUBA1A, TUBA1B and TUBA1C protein sequence. (DOCX)

S8 Fig CLUSTAL O(1.2.4) Multiple Sequence Alignment of *Tubb2a* and *Tubb2b* mRNA. (DOCX)

S9 Fig CLUSTAL O(1.2.4) Multiple Sequence Alignment of *Tuba1a*, *Tuba1b*, and *Tuba1c* mRNA sequences. (DOCX)

S10 Fig **Analysis of *Tubb2a* and *Tubb2b* genomic DNA (A,C) and cDNA (B,D) in *Tubb2a* and *Tubb2b* mutants**.
(DOCX)

S11 Fig *Tubb2b* mRNA in situ probe sequence from Jaglin et al., and homologous Tubb2a sequence. (DOCX)

S12 Fig Alignment of amino acids 400–434 of cow TUBB with 6 mouse β-tublin genes. (DOCX)

S13 Fig Alignment of amino acids 426–450 of human TUBA1A sequence with seven mouse α-tubulin genes. (DOCX)

S1 Table CRISPR guide and PCR primers sequences. (DOCX)

S2 Table Predicted “off-target” sites for CRISPR guides. (DOCX)

S3 Table *Tubb2a* and *Tubb2b* deletion allele statistical analysis. (DOCX)

S4 Table Statistical analysis of immunohistochemistry for layer markers in *Tubb2a* and *Tubb2b* deletion mutants. (DOCX)

S5 Table Statistical analysis of immunoblotting for total beta-tubulin and TUBB3 in *Tubb2a* and *Tubb2b* deletion alleles. (DOCX)

S6 Table Statistical analysis of width of ventricular zone, intermediate zone, and cortical plate, in *Tuba1a* deletion alleles. (DOCX)

S7 Table Neurogenesis and migration in *Tuba1a* mutants. (DOCX)

S8 Table Statistical analysis of immunoblotting for total α-tubulin and β-tubulin in *Tuba1a* mutants. (DOCX)
