# Supplementary figures and images for: Differential requirements of tubulin genes in mammalian forebrain development
Source: PLoS Genet. 2019 Aug 6;15(8):e1008243. doi: 10.1371/journal.pgen.1008243 (PMC6697361; doi:10.1371/journal.pgen.1008243)

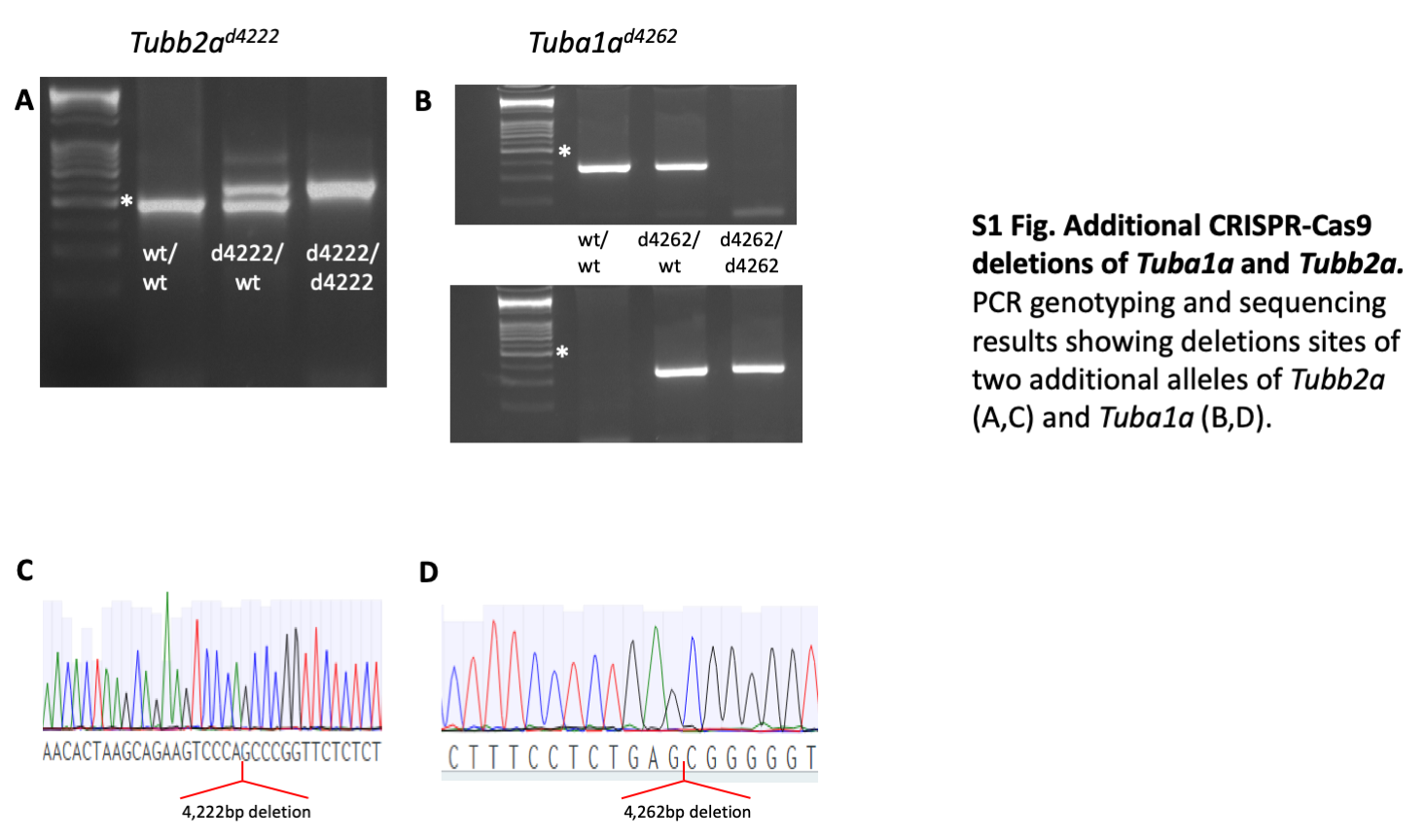

Supplement: S1 Fig — Additional CRISPR-Cas9 deletions of Tubb2a (A,C) and Tuba1a (B,D). (TIF) [file pgen.1008243.s001.tif]

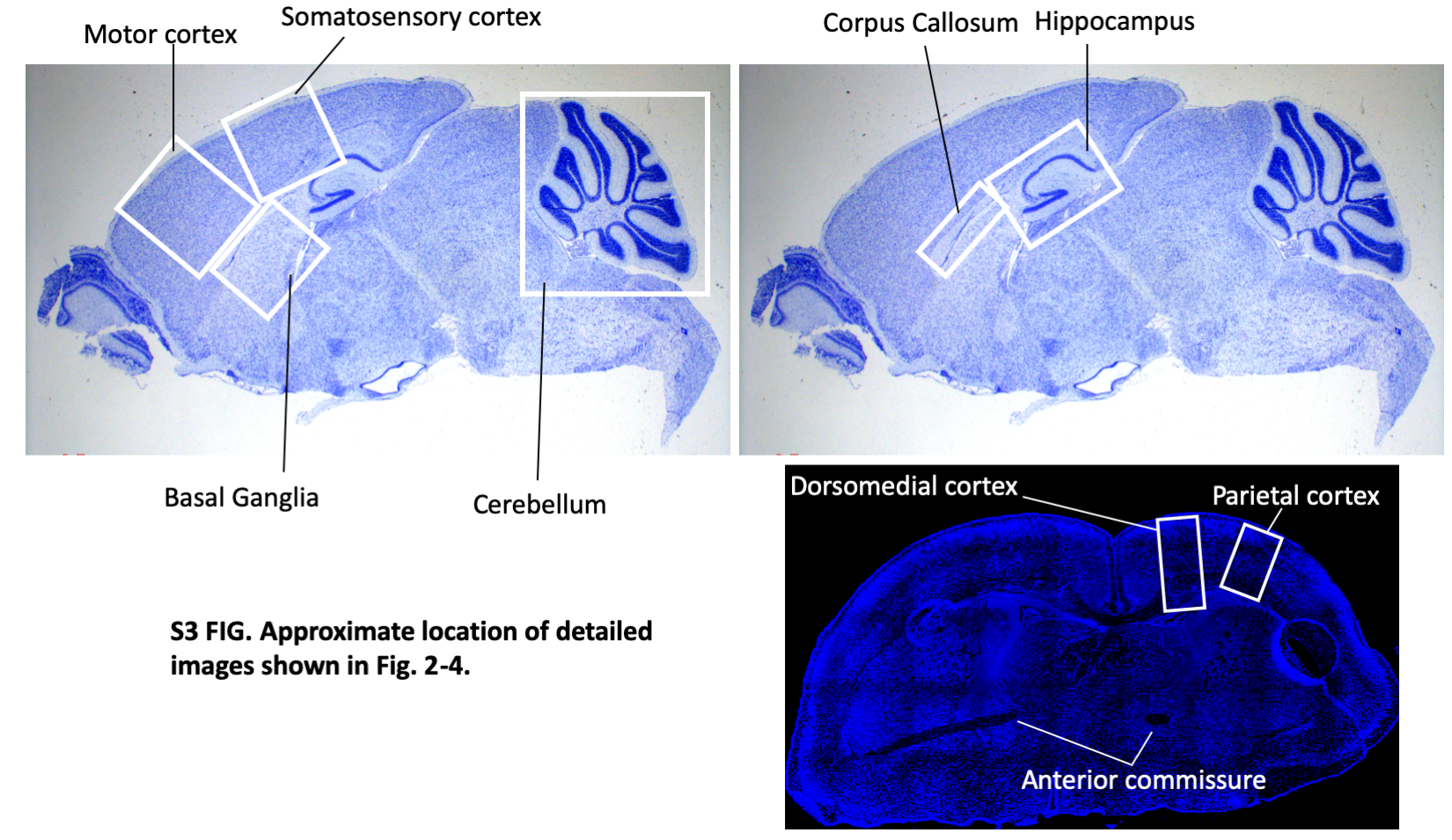

Supplement: S3 Fig — (TIF) [file pgen.1008243.s003.tif]

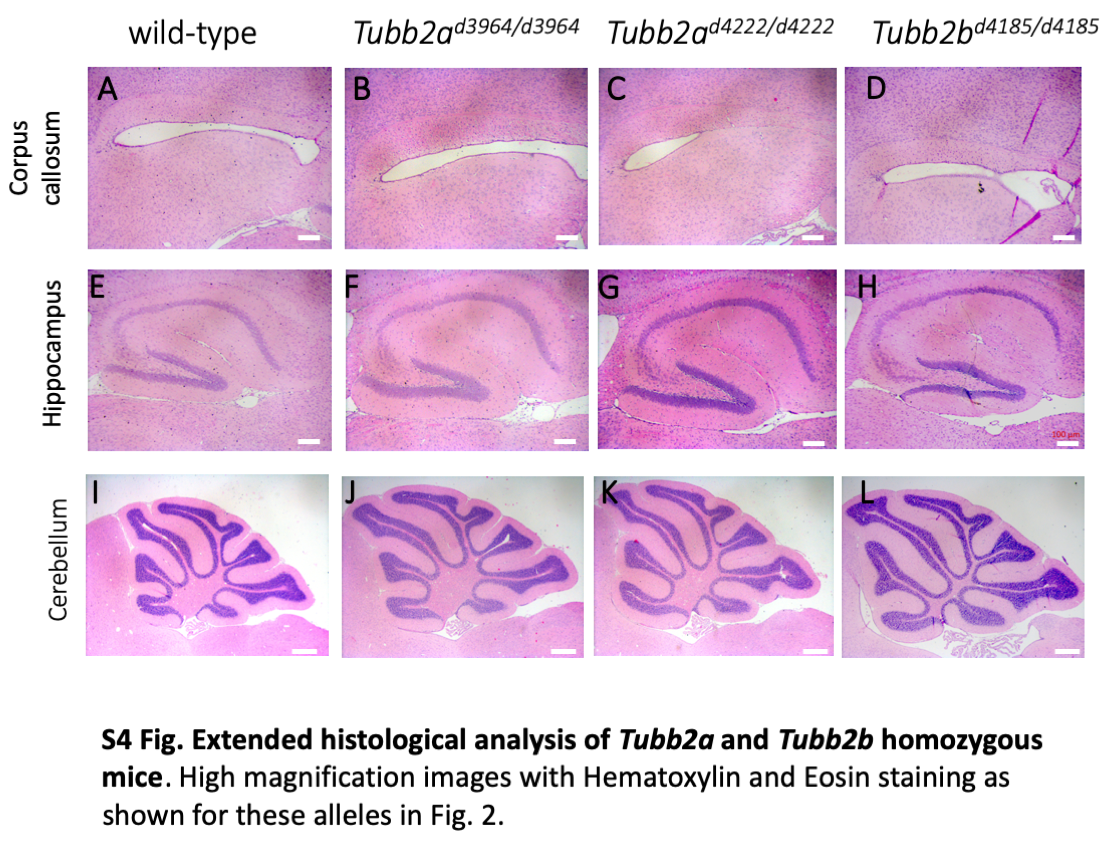

Supplement: S4 Fig — High magnification images with Hematoxylin and Eosin staining as shown for these alleles in Fig 2 for wild-type (A,E,I), Tubb2ad3964 (B,F,J), Tubb2ad4222 (C,G,K), and Tubb2bd4185 (D,H,L) homozygous mice. (TIF) [file pgen.1008243.s004.tif]

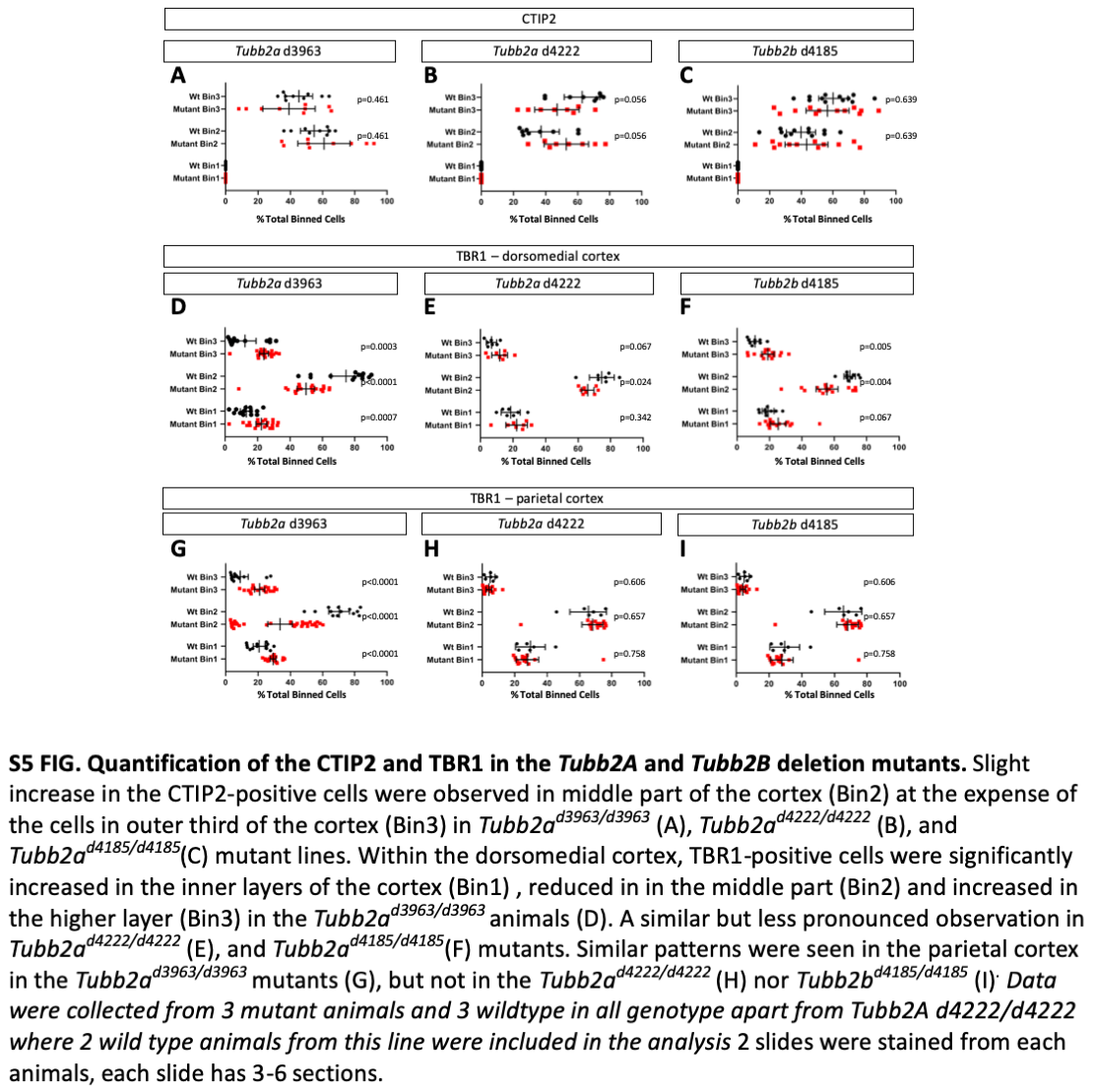

Supplement: S5 Fig — Quantification of the CTIP2 (A-C) and TBR1 (D-I) in the Tubb2a (A,B,D,E,G,H) and Tubb2b (C,F,I) deletion mutants. (TIF) [file pgen.1008243.s005.tif]
