# Supplementary material for: Differential requirements of tubulin genes in mammalian forebrain development
Source: PLoS Genet. 2019 Aug 6;15(8):e1008243. doi: 10.1371/journal.pgen.1008243 (PMC6697361; doi:10.1371/journal.pgen.1008243)
Supplement: S2 Fig — Extended PCR analysis of Tubb2a (A), Tubb2b (B), and Tuba1a (C) deletion alleles. (PDF) [file pgen.1008243.s002.pdf]

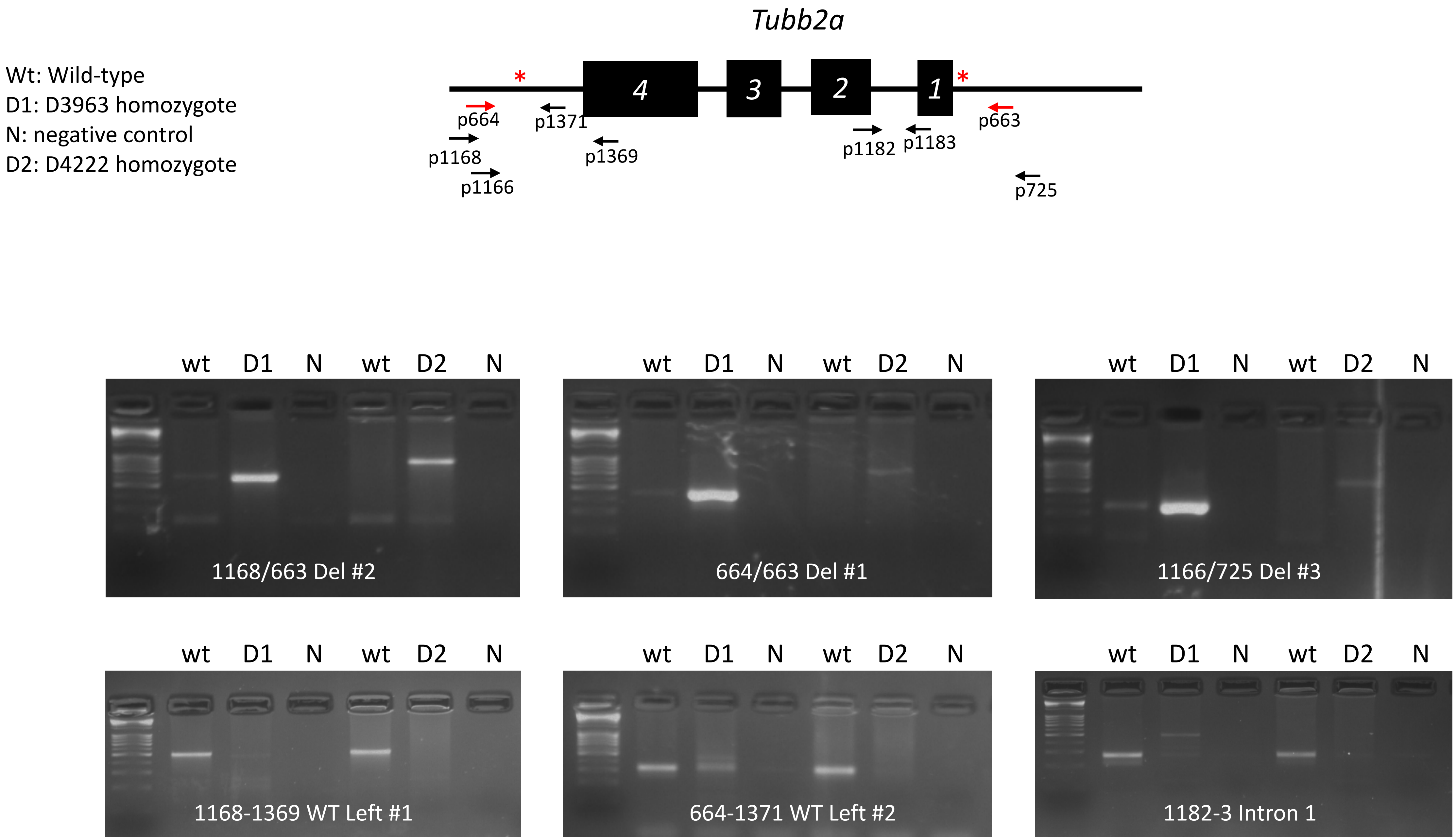

S2 Fig. (A) Extended PCR analysis of *Tubb2a* deletion alleles.

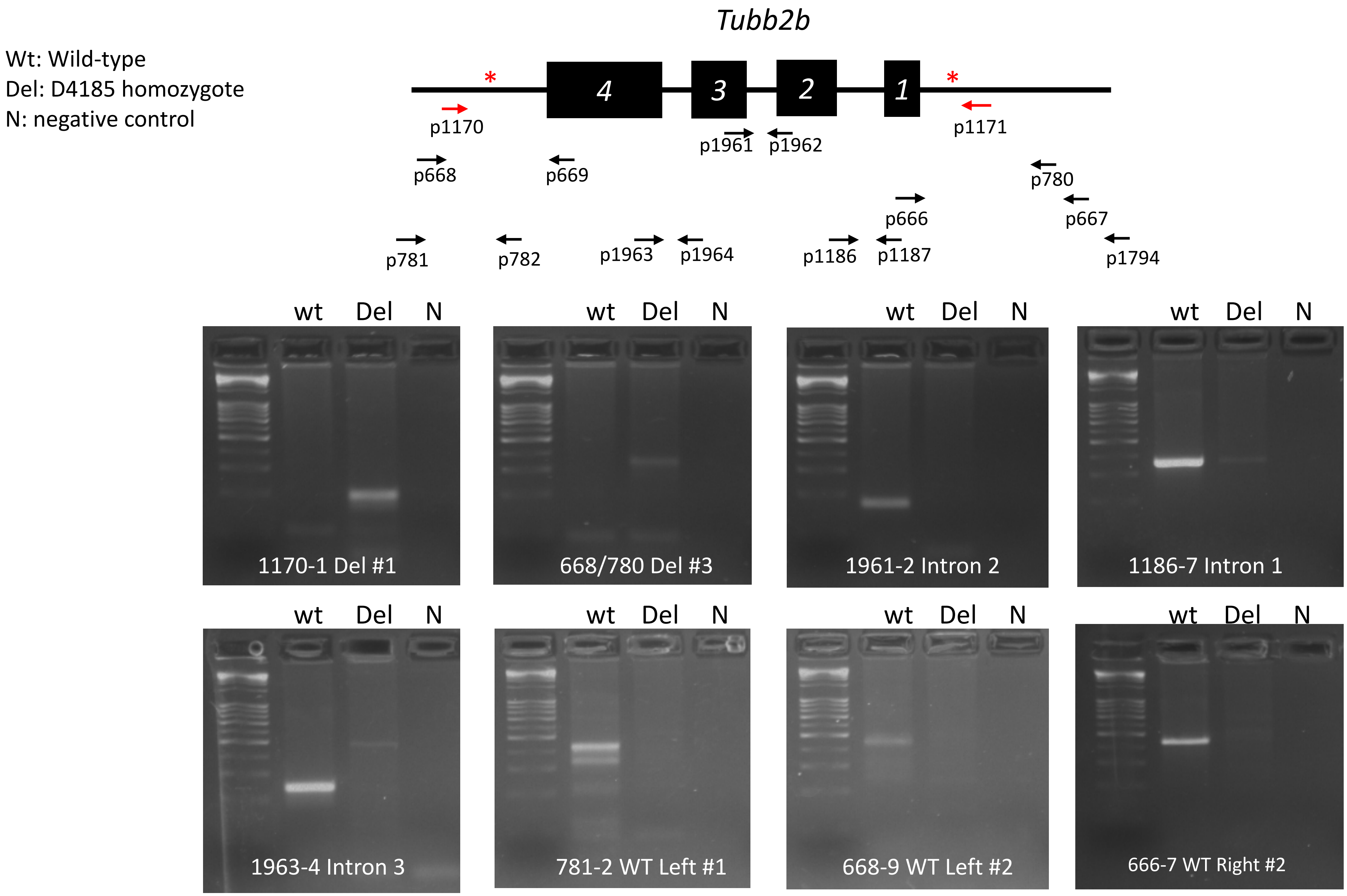

S2 Fig. (B) Extended PCR analysis of *Tubb2b* deletion alleles.

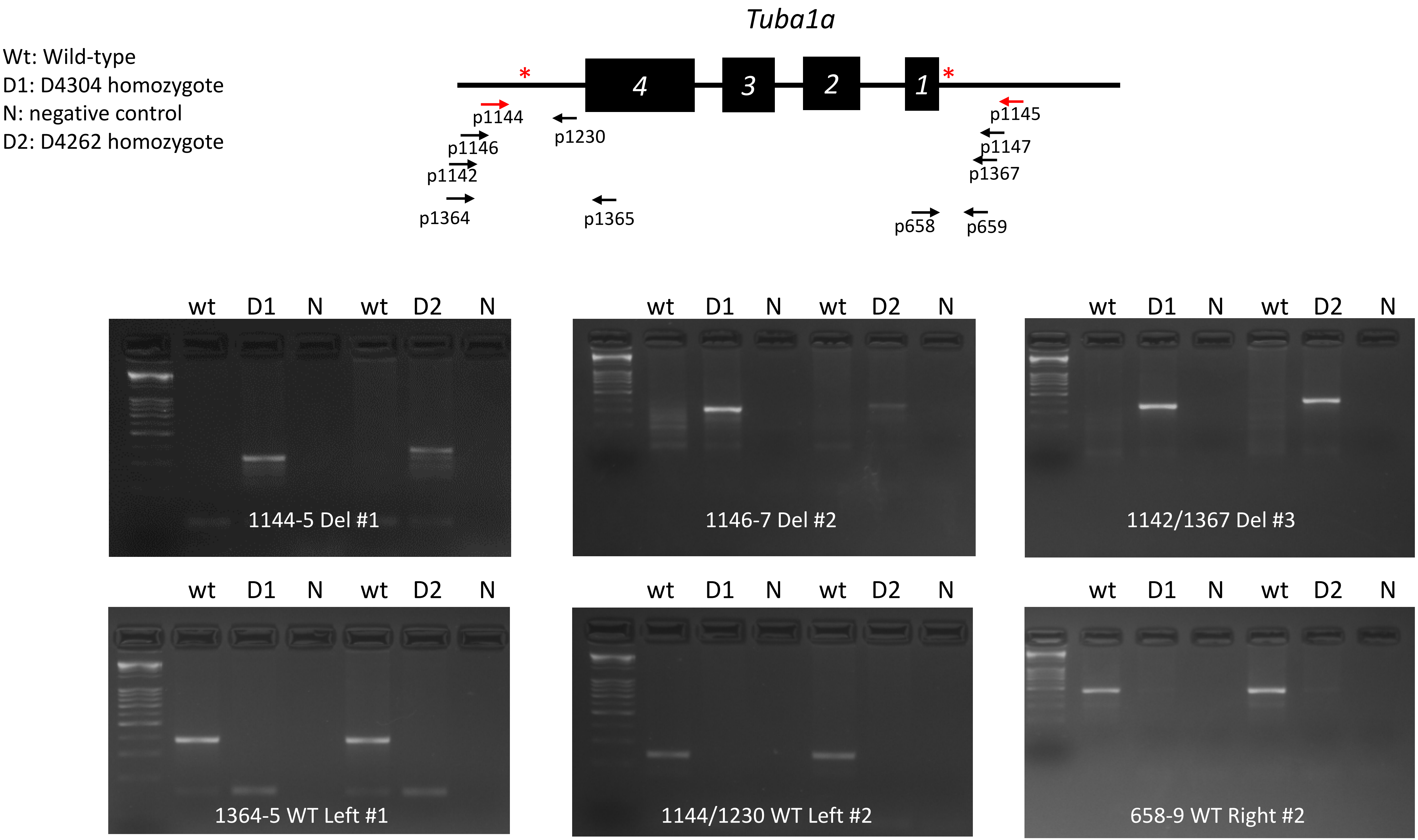

S2 Fig. (C) Extended PCR analysis of *Tuba1a* deletion alleles.
