# Supplementary material for: Differential requirements of tubulin genes in mammalian forebrain development
Source: PLoS Genet. 2019 Aug 6;15(8):e1008243. doi: 10.1371/journal.pgen.1008243 (PMC6697361; doi:10.1371/journal.pgen.1008243)
Supplement: S6 Fig — (DOCX) [file pgen.1008243.s006.docx]

mTubb2a_NP_033476 MREIVHIQAGQCGNQIGAKFWEVISDEHGIDPTGSYHGDSDLQLERINVYYNEAAGNKYV

mTubb2b_NP_076205 MREIVHIQAGQCGNQIGAKFWEVISDEHGIDPTGSYHGDSDLQLERINVYYNEATGNKYV

******************************************************:*****

mTubb2a_NP_033476 PRAILVDLEPGTMDSVRSGPFGQIFRPDNFVFGQSGAGNNWAKGHYTEGAELVDSVLDVV

mTubb2b_NP_076205 PRAILVDLEPGTMDSVRSGPFGQIFRPDNFVFGQSGAGNNWAKGHYTEGAELVDSVLDVV

************************************************************

mTubb2a_NP_033476 RKESESCDCLQGFQLTHSLGGGTGSGMGTLLISKIREEYPDRIMNTFSVMPSPKVSDTVV

mTubb2b_NP_076205 RKESESCDCLQGFQLTHSLGGGTGSGMGTLLISKIREEYPDRIMNTFSVMPSPKVSDTVV

************************************************************

mTubb2a_NP_033476 EPYNATLSVHQLVENTDETYSIDNEALYDICFRTLKLTTPTYGDLNHLVSATMSGVTTCL

mTubb2b_NP_076205 EPYNATLSVHQLVENTDETYCIDNEALYDICFRTLKLTTPTYGDLNHLVSATMSGVTTCL

********************.***************************************

mTubb2a_NP_033476 RFPGQLNADLRKLAVNMVPFPRLHFFMPGFAPLTSRGSQQYRALTVPELTQQMFDSKNMM

mTubb2b_NP_076205 RFPGQLNADLRKLAVNMVPFPRLHFFMPGFAPLTSRGSQQYRALTVPELTQQMFDSKNMM

************************************************************

mTubb2a_NP_033476 AACDPRHGRYLTVAAIFRGRMSMKEVDEQMLNVQNKNSSYFVEWIPNNVKTAVCDIPPRG

mTubb2b_NP_076205 AACDPRHGRYLTVAAIFRGRMSMKEVDEQMLNVQNKNSSYFVEWIPNNVKTAVCDIPPRG

************************************************************

mTubb2a_NP_033476 LKMSATFIGNSTAIQELFKRISEQFTAMFRRKAFLHWYTGEGMDEMEFTEAESNMNDLVS

mTubb2b_NP_076205 LKMSATFIGNSTAIQELFKRISEQFTAMFRRKAFLHWYTGEGMDEMEFTEAESNMNDLVS

************************************************************

mTubb2a_NP_033476 EYQQYQDATADEQGEFEEEEGEDEA

mTubb2b_NP_076205 EYQQYQDATADEQGEFEEEEGEDEA

*************************

**S6 FIG. CLUSTAL 2.1 multiple sequence alignment of TUBB2A and TUBB2B protein sequence.**
