# Supplementary material for: Differential requirements of tubulin genes in mammalian forebrain development
Source: PLoS Genet. 2019 Aug 6;15(8):e1008243. doi: 10.1371/journal.pgen.1008243 (PMC6697361; doi:10.1371/journal.pgen.1008243)
Supplement: S7 Fig — (DOCX) [file pgen.1008243.s007.docx]

mTuba1a_NP_035783 MRECISIHVGQAGVQIGNACWELYCLEHGIQPDGQMPSDKTIGGGDDSFNTFFSETGAGK

mTuba1b_NP_035784 MRECISIHVGQAGVQIGNACWELYCLEHGIQPDGQMPSDKTIGGGDDSFNTFFSETGAGK

mTuba1c_NP_033474 MRECISIHVGQAGVQIGNACWELYCLEHGIQPDGQMPSDKTIGGGDDSFNTFFSETGAGK

************************************************************

mTuba1a_NP_035783 HVPRAVFVDLEPTVIDEVRTGTYRQLFHPEQLITGKEDAANNYARGHYTIGKEIIDLVLD

mTuba1b_NP_035784 HVPRAVFVDLEPTVIDEVRTGTYRQLFHPEQLITGKEDAANNYARGHYTIGKEIIDLVLD

mTuba1c_NP_033474 HVPRAVFVDLEPTVIDEVRTGTYRQLFHPEQLITGKEDAANNYARGHYTIGKEIIDLVLD

************************************************************

mTuba1a_NP_035783 RIRKLADQCTGLQGFLVFHSFGGGTGSGFTSLLMERLSVDYGKKSKLEFSIYPAPQVSTA

mTuba1b_NP_035784 RIRKLADQCTGLQGFLVFHSFGGGTGSGFTSLLMERLSVDYGKKSKLEFSIYPAPQVSTA

mTuba1c_NP_033474 RIRKLADQCTGLQGFLVFHSFGGGTGSGFTSLLMERLSVDYGKKSKLEFSIYPAPQVSTA

************************************************************

mTuba1a_NP_035783 VVEPYNSILTTHTTLEHSDCAFMVDNEAIYDICRRNLDIERPTYTNLNRLIGQIVSSITA

mTuba1b_NP_035784 VVEPYNSILTTHTTLEHSDCAFMVDNEAIYDICRRNLDIERPTYTNLNRLISQIVSSITA

mTuba1c_NP_033474 VVEPYNSILTTHTTLEHSDCAFMVDNEAIYDICRRNLDIERPTYTNLNRLISQIVSSITA

***************************************************.********

mTuba1a_NP_035783 SLRFDGALNVDLTEFQTNLVPYPRIHFPLATYAPVISAEKAYHEQLSVAEITNACFEPAN

mTuba1b_NP_035784 SLRFDGALNVDLTEFQTNLVPYPRIHFPLATYAPVISAEKAYHEQLSVAEITNACFEPAN

mTuba1c_NP_033474 SLRFDGALNVDLTEFQTNLVPYPRIHFPLATYAPVISAEKAYHEQLTVAEITNACFEPAN

**********************************************:*************

mTuba1a_NP_035783 QMVKCDPRHGKYMACCLLYRGDVVPKDVNAAIATIKTKRTIQFVDWCPTGFKVGINYQPP

mTuba1b_NP_035784 QMVKCDPRHGKYMACCLLYRGDVVPKDVNAAIATIKTKRSIQFVDWCPTGFKVGINYQPP

mTuba1c_NP_033474 QMVKCDPRHGKYMACCLLYRGDVVPKDVNAAIATIKTKRTIQFVDWCPTGFKVGINYQPP

***************************************:********************

mTuba1a_NP_035783 TVVPGGDLAKVQRAVCMLSNTTAIAEAWARLDHKFDLMYAKRAFVHWYVGEGMEEGEFSE

mTuba1b_NP_035784 TVVPGGDLAKVQRAVCMLSNTTAIAEAWARLDHKFDLMYAKRAFVHWYVGEGMEEGEFSE

mTuba1c_NP_033474 TVVPGGDLAKVQRAVCMLSNTTAIAEAWARLDHKFDLMYAKRAFVHWYVGEGMEEGEFSE

************************************************************

mTuba1a_NP_035783 AREDMAALEKDYEEVGVDSVEGEGEEEGEEY

mTuba1b_NP_035784 AREDMAALEKDYEEVGVDSVEGEGEEEGEEY

mTuba1c_NP_033474 AREDMAALEKDYEEVGADSAEGDDEGEEY--

****************.**.**:.* *

**S7 FIG. CLUSTAL 2.1 multiple sequence alignment of TUBA1A, TUBA1B, and TUBA1C protein sequence.**
