# Supplementary material for: Differential requirements of tubulin genes in mammalian forebrain development
Source: PLoS Genet. 2019 Aug 6;15(8):e1008243. doi: 10.1371/journal.pgen.1008243 (PMC6697361; doi:10.1371/journal.pgen.1008243)
Supplement: S8 Fig — (DOCX) [file pgen.1008243.s008.docx]

1. ***Tubb2a* and *Tubb2b* mRNA**

mTubb2a_NM_009450.2 --------------ggtcttttgcgctccttag--ccctctgtccacgcactgc---tcc

mTubb2b_NM_023716.2 ctcagcccgtagcccgtcggttccgga-gtaagttccaggtggcccagcagtgggtgtgg

*** ** ** * ** ** ** ** *** ** *

mTubb2a_NM_009450.2 gagggcaagagcctccacccctt-------------------cta------caaccagca

mTubb2b_NM_023716.2 aaggggaggatcatcagacccactgacacagacccaagacagcaagaagctaaccaggca

**** * ** * ** *** * * * * ***

mTubb2a_NM_009450.2 ccatgcgcgagatcgtgcacatccaggcgggccagtgtggcaaccagatcggcgctaagt

mTubb2b_NM_023716.2 ccatgcgagagatcgtgcacattcaggcgggccagtgcggcaaccagatcggtgccaagt

******* ************** ************** ************** ** ****

mTubb2a_NM_009450.2 tttgggaggtgataagcgatgagcatggcatcgaccccactggcagttaccatggcgaca

mTubb2b_NM_023716.2 tttgggaggtcatcagtgatgagcatggtatagaccccactggaagttaccatggagaca

********** ** ** *********** ** *********** *********** ****

mTubb2a_NM_009450.2 gtgacttgcagctggagaggatcaatgtgtactacaatgaagctgctggcaacaaatatg

mTubb2b_NM_023716.2 gtgatttgcaactggaaagaatcaatgtatactacaatgaagcaactggtaataaatatg

**** ***** ***** ** ******** ************** **** ** *******

mTubb2a_NM_009450.2 tacctcgggccatcctagtggacctggagccgggcaccatggactcagtgaggtcgggac

mTubb2b_NM_023716.2 tgcctagggccatcctggtggacctggagccaggcacaatggactcagtcaggtctggac

* *** ********** ************** ***** *********** ***** ****

mTubb2a_NM_009450.2 cattcggccagatcttcaggccagacaactttgtgttcggccagagtggtgcaggaaata

mTubb2b_NM_023716.2 catttgggcagatcttcaggccggacaacttcgtgttcggccagagtggtgcaggaaata

**** ** ************** ******** ****************************

mTubb2a_NM_009450.2 actgggcaaaaggccactacacagagggggccgagctggtggactctgtcctagatgtgg

mTubb2b_NM_023716.2 actgggcaaagggccactacacagagggggccgagctggtggactctgtcctggacgtgg

********** ***************************************** ** ****

mTubb2a_NM_009450.2 tgaggaaggagtctgaaagctgtgactgtctccagggtttccagctgacccactcactgg

mTubb2b_NM_023716.2 tgaggaaggagtcagaaagctgtgactgtcttcagggcttccagctgacccactcactgg

************* ***************** ***** **********************

mTubb2a_NM_009450.2 ggggaggcactggctcaggcatggggaccctgctcatcagcaagatcagagaggagtacc

mTubb2b_NM_023716.2 ggggaggcactggctcaggcatggggaccctgctcatcagcaagatcagagaggagtacc

************************************************************

mTubb2a_NM_009450.2 cagaccgcatcatgaacaccttcagcgtcatgccctcacccaaggtctctgatactgtgg

mTubb2b_NM_023716.2 cagaccgcatcatgaacaccttcagcgtcatgccctcacccaaggtctctgacactgtgg

**************************************************** *******

mTubb2a_NM_009450.2 tggagccctataatgccaccctctcagtgcaccagctggtagagaacacagatgaaacct

mTubb2b_NM_023716.2 tggagccctataatgccaccctctcagtgcaccagctggtagagaacacagatgaaacct

************************************************************

mTubb2a_NM_009450.2 attccattgacaatgaggctctgtatgacatctgtttccgcaccctgaagctgaccacac

mTubb2b_NM_023716.2 actgcatcgataatgaggctctgtatgacatctgtttccgcaccctgaagctgaccacac

* * *** ** *************************************************

mTubb2a_NM_009450.2 ccacctatggcgatctcaaccacctggtgtcagccaccatgagtggagtgaccacctgcc

mTubb2b_NM_023716.2 ccacctatggcgatctcaaccacctggtgtcagccaccatgagtggagtgaccacctgcc

************************************************************

mTubb2a_NM_009450.2 tgcgcttcccaggccagctgaacgcagacctgcgcaagctggccgtgaacatggtgccat

mTubb2b_NM_023716.2 tgcgcttcccaggccagctgaacgcagacctgcgcaagctggccgtgaacatggtgccat

************************************************************

mTubb2a_NM_009450.2 tcccacgcctgcacttcttcatgccaggctttgcacctctgaccagccggggcagccagc

mTubb2b_NM_023716.2 tcccacgcctgcacttcttcatgccaggctttgcacctctgaccagccggggcagccagc

************************************************************

mTubb2a_NM_009450.2 agtaccgagccctgacggtgcccgagctgacccagcagatgttcgactccaagaacatga

mTubb2b_NM_023716.2 agtaccgagccctgacggtgcccgagctgacccagcagatgttcgactccaagaacatga

************************************************************

mTubb2a_NM_009450.2 tggctgcctgcgaccctcgccatggccgctacctgaccgtggccgccattttccgtggcc

mTubb2b_NM_023716.2 tggctgcctgcgaccctcgccatggccgctacctgaccgtggccgccattttccgtggcc

************************************************************

mTubb2a_NM_009450.2 gcatgtccatgaaggaggtggatgagcagatgctcaacgtgcagaacaagaacagcagct

mTubb2b_NM_023716.2 gcatgtccatgaaggaggtggatgagcagatgctcaacgtgcagaacaagaacagcagct

************************************************************

mTubb2a_NM_009450.2 acttcgtggagtggatccccaacaacgtcaagacggccgtgtgtgacatccctcctcgtg

mTubb2b_NM_023716.2 acttcgtggagtggatccccaacaacgtcaagacggccgtgtgtgacatccctcctcgtg

************************************************************

mTubb2a_NM_009450.2 gcctcaagatgtcagccaccttcattggcaacagcactgccatccaggagctgttcaagc

mTubb2b_NM_023716.2 gcctcaagatgtcagccaccttcattggcaacagcactgccatccaggagctgttcaagc

************************************************************

mTubb2a_NM_009450.2 gcatctcggagcagttcactgccatgttccggcgcaaggctttcctgcactggtacacgg

mTubb2b_NM_023716.2 gcatctcggagcagttcactgctatgttccggcgcaaggctttcctgcactggtacacgg

********************** *************************************

mTubb2a_NM_009450.2 gtgagggcatggacgagatggagttcaccgaggcggagagcaacatgaatgacctggtgt

mTubb2b_NM_023716.2 gtgagggcatggacgagatggagttcaccgaggcggagagcaacatgaatgacctggtgt

************************************************************

mTubb2a_NM_009450.2 ctgagtaccagcagtaccaggatgccacggctgatgagcagggcgagttcgaggaggagg

mTubb2b_NM_023716.2 ctgagtaccagcagtaccaggatgccacggctgatgagcagggcgagttcgaggaggaag

********************************************************** *

mTubb2a_NM_009450.2 agggtgaagatgaggcttgagaacttc-----------------------tcagataca-

mTubb2b_NM_023716.2 agggcgaggatgaggcttgagttccccaggccaagcaggttagggaaagctgagatgaaa

**** ** ************* * * * **** *

mTubb2a_NM_009450.2 -------gtgtgcaccctta----gtgaacttctgttgtcc---------tccagcatgg

mTubb2b_NM_023716.2 ggagggggtgggggggcttaatctgtgaaaataccttggcagttgaaggaaggagaatgg

*** * **** ***** * *** * ** ****

mTubb2a_NM_009450.2 tctt--------------------------------------------------tc--ta

mTubb2b_NM_023716.2 tcttaggtttgtgctgggtctctggtgctcttcactgttgcctgtcactttttttctctt

**** ** *

mTubb2a_NM_009450.2 tttgtaaattatggt---g--------ctcagtttgcctctgtcagaaattcactgttga

mTubb2b_NM_023716.2 tttgtaa--taccgataacatcaatgtaacacttgagatctttctgaactc-----ctgt

******* ** * ** ** *** ** *** * **

mTubb2a_NM_009450.2 tgtaatagt---------gtgaacctctttcaagatcacagtattgt-------------

mTubb2b_NM_023716.2 tgtaatggctaaaatcacataaacctttgtg-tcctaacggtgtcctcttttctttctct

****** * * ***** * * * ** ** * *

mTubb2a_NM_009450.2 ------ctc---agaaatct-----atatgaataaaaaagcatgtgtataaaaaaaaaaa

mTubb2b_NM_023716.2 tcctttctccctatcaagctctttgttatcaacttaaatccacctttctgaacacagaaa

*** * ** ** *** ** *** ** * * * ** * * ***

mTubb2a_NM_009450.2 aa----------------------------------------------------------

mTubb2b_NM_023716.2 attttcttcctttagaaaagactgaaagctcaggtgtttgtttcttctttgggtatgcta

*

mTubb2a_NM_009450.2 ------------------aaa----------------aaaaaaaaaaaaaaaaaaa----

mTubb2b_NM_023716.2 ttaatataagttgaaccaaaaatggccttactcaatccaacaatgagaagaaacaatgga

*** ** ** * ** *** **

mTubb2a_NM_009450.2 -----------

mTubb2b_NM_023716.2 ttttaagatgt

**B.5’UTR**

mTubb2a__NM_009450.2_5’UTR ggtcttttgcgctccttagccc-tc---------------------tgtccacgcactgc 38

mTubb2b_NM_023716.2_5’UTR ------ctcag-cccgtagcccgtcggttccggagtaagttccaggtggcccagcagtgg 53

* * ** ****** ** ** ** *** **

mTubb2a_NM_009450.2_5’UTR ---tccgagggcaagagcctccaccccttctacaaccagcaccatgcgcgagatcgtgca 95

mTubb2b_NM_023716.2_5’UTR gtgtggaaggggaggatcatcagacccactgaca------------------------ca 89

* **** * ** * ** *** *** **

mTubb2a_NM_009450.2_5’UTR catccaggcgggccagtgtggcaaccagatcggcgctaag 135

mTubb2b_NM_023716.2_5’UTR gacccaagacagcaag-aagctaaccagg----cacc--- 121

* *** * ** ** * ****** * *

1. **exon2**

mTubb2a_NM_009450.2_exon 2 ttttgggaggtgataagcgatgagcatggcatcgaccccactggcagttaccatggcgac 60

mTubb2b_NM_023716.2_exon 2 ttttgggaggtcatcagtgatgagcatggtatagaccccactggaagttaccatggagac 60

*********** ** ** *********** ** *********** *********** ***

mTubb2a_NM_009450.2_exon 2 agtgacttgcagctggagaggatcaatgtgtactacaatgaagctgctg 109

mTubb2b_NM_023716.2_exon 2 agtgatttgcaactggaaagaatcaatgtatactacaatgaagcaactg 109

***** ***** ***** ** ******** ************** ***

1. **exon3**

mTubb2a_NM_009450.2_exon 3 gcaacaaatatgtacctcgggccatcctagtggacctggagccgggcaccatggactcag 60

mTubb2b_NM_023716.2_exon 3 gtaataaatatgtgcctagggccatcctggtggacctggagccaggcacaatggactcag 60

* ** ******** *** ********** ************** ***** **********

mTubb2a_NM_009450.2_exon 3 tgaggtcgggaccattcg--------------------------------- 78

mTubb2b_NM_023716.2_exon 3 tcaggtctggaccatttgggcagatcttcaggccggacaacttcgtgttcg 111

* ***** ******** *

1. **exon 4**

mTubb2a_NM_009450.2_exon 4 gccagatcttcaggccagacaactttgtgttcggccagagtggtgcaggaaataactggg 60

mTubb2b_NM_023716.2_exon 4 ---------------------------------gccagagtggtgcaggaaataactggg 27

***************************

mTubb2a_NM_009450.2_exon 4 caaaaggccactacacagagggggccgagctggtggactctgtcctagatgtggtgagga 120

mTubb2b_NM_023716.2_exon 4 caaagggccactacacagagggggccgagctggtggactctgtcctggacgtggtgagga 87

**** ***************************************** ** **********

mTubb2a_NM_009450.2_exon 4 aggagtctgaaagctgtgactgtctccagggtttccagctgacccactcactggggggag 180

mTubb2b_NM_023716.2_exon 4 aggagtcagaaagctgtgactgtcttcagggcttccagctgacccactcactggggggag 147

******* ***************** ***** ****************************

mTubb2a_NM_009450.2_exon 4 gcactggctcaggcatggggaccctgctcatcagcaagatcagagaggagtacccagacc 240

mTubb2b_NM_023716.2_exon 4 gcactggctcaggcatggggaccctgctcatcagcaagatcagagaggagtacccagacc 207

************************************************************

mTubb2a_NM_009450.2_exon 4 gcatcatgaacaccttcagcgtcatgccctcacccaaggtctctgatactgtggtggagc 300

mTubb2b_NM_023716.2_exon 4 gcatcatgaacaccttcagcgtcatgccctcacccaaggtctctgacactgtggtggagc 267

********************************************** *************

mTubb2a_NM_009450.2_exon 4 cctataatgccaccctctcagtgcaccagctggtagagaacacagatgaaacctattcca 360

mTubb2b_NM_023716.2_exon 4 cctataatgccaccctctcagtgcaccagctggtagagaacacagatgaaacctactgca 327

******************************************************* * **

mTubb2a_NM_009450.2_exon 4 ttgacaatgaggctctgtatgacatctgtttccgcaccctgaagctgaccacacccacct 420

mTubb2b_NM_023716.2_exon 4 tcgataatgaggctctgtatgacatctgtttccgcaccctgaagctgaccacacccacct 387

* ** *******************************************************

mTubb2a_NM_009450.2_exon 4 atggcgatctcaaccacctggtgtcagccaccatgagtggagtgaccacctgcctgcgct 480

mTubb2b_NM_023716.2_exon 4 atggcgatctcaaccacctggtgtcagccaccatgagtggagtgaccacctgcctgcgct 447

************************************************************

mTubb2a_NM_009450.2_exon 4 tcccaggccagctgaacgcagacctgcgcaagctggccgtgaacatggtgccattcccac 540

mTubb2b_NM_023716.2_exon 4 tcccaggccagctgaacgcagacctgcgcaagctggccgtgaacatggtgccattcccac 507

************************************************************

mTubb2a_NM_009450.2_exon 4 gcctgcacttcttcatgccaggctttgcacctctgaccagccggggcagccagcagtacc 600

mTubb2b_NM_023716.2_exon 4 gcctgcacttcttcatgccaggctttgcacctctgaccagccggggcagccagcagtacc 567

************************************************************

mTubb2a_NM_009450.2_exon 4 gagccctgacggtgcccgagctgacccagcagatgttcgactccaagaacatgatggctg 660

mTubb2b_NM_023716.2_exon 4 gagccctgacggtgcccgagctgacccagcagatgttcgactccaagaacatgatggctg 627

************************************************************

mTubb2a_NM_009450.2_exon 4 cctgcgaccctcgccatggccgctacctgaccgtggccgccattttccgtggccgcatgt 720

mTubb2b_NM_023716.2_exon 4 cctgcgaccctcgccatggccgctacctgaccgtggccgccattttccgtggccgcatgt 687

************************************************************

mTubb2a_NM_009450.2_exon 4 ccatgaaggaggtggatgagcagatgctcaacgtgcagaacaagaacagcagctacttcg 780

mTubb2b_NM_023716.2_exon 4 ccatgaaggaggtggatgagcagatgctcaacgtgcagaacaagaacagcagctacttcg 747

************************************************************

mTubb2a_NM_009450.2_exon 4 tggagtggatccccaacaacgtcaagacggccgtgtgtgacatccctcctcgtggcctca 840

mTubb2b_NM_023716.2_exon 4 tggagtggatccccaacaacgtcaagacggccgtgtgtgacatccctcctcgtggcctca 807

************************************************************

mTubb2a_NM_009450.2_exon 4 agatgtcagccaccttcattggcaacagcactgccatccaggagctgttcaagcgcatct 900

mTubb2b_NM_023716.2_exon 4 agatgtcagccaccttcattggcaacagcactgccatccaggagctgttcaagcgcatct 867

************************************************************

mTubb2a_NM_009450.2_exon 4 cggagcagttcactgccatgttccggcgcaaggctttcctgcactggtacacgggtgagg 960

mTubb2b_NM_023716.2_exon 4 cggagcagttcactgctatgttccggcgcaaggctttcctgcactggtacacgggtgagg 927

**************** *******************************************

mTubb2a_NM_009450.2_exon 4 gcatggacgagatggagttcaccgaggcggagagcaacatgaatgacctggtgtctgagt 1020

mTubb2b_NM_023716.2_exon 4 gcatggacgagatggagttcaccgaggcggagagcaacatgaatgacctggtgtctgagt 987

************************************************************

mTubb2a_NM_009450.2_exon 4 accagcagtaccaggatgccacggctgatgagcagggcgagttcgaggaggaggagggtg 1080

mTubb2b_NM_023716.2_exon 4 accagcagtaccaggatgccacggctgatgagcagggcgagttcgaggaggaagagggcg 1047

**************************************************** ***** *

mTubb2a_NM_009450.2_exon 4 aagatgaggcttga 1094

mTubb2b_NM_023716.2_exon 4 aggatgaggcttga 1061

* ************

1. **3’UTR**

mTubb2a_NM_009450.2_3’UTR ------------------------------------------------------------ 0

mTubb2b_NM_023716.2_3’UTR gttccccaggccaagcaggttagggaaagctgagatgaaaggagggggtgggggggctta 60

mTubb2a_NM_009450.2_3’UTR ------------------------------------------------------------ 0

mTubb2b_NM_023716.2_3’UTR atctgtgaaaataccttggcagttgaaggaaggagaatggtcttaggtttgtgctgggtc 120

mTubb2a_NM_009450.2_3’UTR -----------------------------------------------------gaacttc 7

mTubb2b_NM_023716.2_3’UTR tctggtgctcttcactgttgcctgtcactttttttctctttttgtaataccgataacatc 180

*** **

mTubb2a_NM_009450.2_3’UTR tcagatacagtgtgcacccttagtgaacttctgttgtcctcca--gcatggtctttctat 65

mTubb2b_NM_023716.2_3’UTR aatgtaacact-tgagatctttctgaactcctgttgtaatggctaaaatcacataaacct 239

* *** * ** *** ****** ******* * ** * *

mTubb2a_NM_009450.2_3’UTR ttgtaa-attatggtgctcagt---------------ttgcctctgtcagaaattcactg 109

mTubb2b_NM_023716.2_3’UTR ttgtgtcctaacggtgtcctcttttctttctcttcctttctccctatc--aagctctttg 297

**** * * **** * * ** * ** ** ** ** **

mTubb2a_NM_009450.2_3’UTR ttgatgtaatagtgtgaacctctttcaagatcacagta--ttgtc-----tcagaaatct 162

mTubb2b_NM_023716.2_3’UTR tta-tcaactt---aaatccacctttctgaacacagaaaattttcttcctttagaa---- 349

** * * * * ** * ** ** ***** * ** ** * ****

mTubb2a_NM_009450.2_3’UTR atatgaataaaaaagcatgtgt-----------------a-----------ta------a 188

mTubb2b_NM_023716.2_3’UTR --aagactgaaagctcaggtgtttgtttcttctttgggtatgctattaatataagttgaa 407

* ** * *** ** **** * ** *

mTubb2a_NM_009450.2_3’UTR aaaaaaaaa--------aaaaaaaaaaaaaaaaaaaaaaaa--------------- 221

mTubb2b_NM_023716.2_3’UTR ccaaaaatggccttactcaatccaacaatgagaagaaacaatggattttaagatgt 463

***** ** ** ** * ** *** **

**S8 FIG. CLUSTAL O(1.2.4) Multiple Sequence Alignment of *Tubb2a* and *Tubb2b* mRNA.** (A) Entire mRNA sequence. (B)5’UTR sequence. (C) Exon 2. (D) Exon 3. (E) Exon 4. (F) 3’UTR sequence.
