# Supplementary material for: Differential requirements of tubulin genes in mammalian forebrain development
Source: PLoS Genet. 2019 Aug 6;15(8):e1008243. doi: 10.1371/journal.pgen.1008243 (PMC6697361; doi:10.1371/journal.pgen.1008243)
Supplement: S9 Fig — (DOCX) [file pgen.1008243.s009.docx]

mTuba1c_NM_009448.4 gaagctgtcgcagtcaccaccctgccggcagccccgggtcacctctctggctcactcttc 60

mTuba1a_NM_011653.2 ------------------------------------------------------------ 0

mTuba1b_NM_011654.2 ------------------------------------------------------------ 0

mTuba1c_NM_009448.4 cgggctccaaggcctgggtggggcgaccgagccacaagtgcggcggcgatcagctgtgag 120

mTuba1a_NM_011653.2 ------------------------------------------------------------ 0

mTuba1b_NM_011654.2 ------------------------------------------------------------ 0

mTuba1c_NM_009448.4 tccccggtggctgagccaggccgcccccctctctacctgggactttcttccggccagagc 180

mTuba1a_NM_011653.2 ------------------------------------------------------------ 0

mTuba1b_NM_011654.2 ------------------------------------------------------------ 0

mTuba1c_NM_009448.4 tccacaaagatctcgctgtcctccaacttccaatcgccttccccaccttcttccccgccc 240

mTuba1a_NM_011653.2 ------------------------------------------------------------ 0

mTuba1b_NM_011654.2 ------------------------------------------------------------ 0

mTuba1c_NM_009448.4 cacctggccctggcgtggaccctgggtgtctcctccccttcccacttccagctccttcta 300

mTuba1a_NM_011653.2 ------------------------------------------------------------ 0

mTuba1b_NM_011654.2 ------------------------------------------------------------ 0

mTuba1c_NM_009448.4 cggagtttgatttgaactttcgaacccgccaatcatcggcttcgctgcggcccggggcgg 360

mTuba1a_NM_011653.2 ------------------------------------------------------------ 0

mTuba1b_NM_011654.2 ------------------------------------------------------------ 0

mTuba1c_NM_009448.4 ggtccggtgtccgccctcccgacgcgtggcccggccagggtatataagccctgtcctgga 420

mTuba1a_NM_011653.2 ------------------------------------------------------------ 0

mTuba1b_NM_011654.2 ------------------------------------------------------------ 0

mTuba1c_NM_009448.4 ggcggaccttacacagcttctcccccggactccttggtagtctgttagtgggagatcttc 480

mTuba1a_NM_011653.2 ------------------------------------------------------------ 0

mTuba1b_NM_011654.2 --------------------------------------------------cgagacccgg 10

mTuba1c_NM_009448.4 -gtcaccct----ttttcacttcctcagttttcgcggaccacttcaaggactaaatatgc 535

mTuba1a_NM_011653.2 --cctcct------cctcgcctccgccatccacccggcagccgcgaagcagcaaccatgc 52

mTuba1b_NM_011654.2 tgtctgcttctatctctcaccctcgccttctaaccc-----------gttgctatcatgc 59

* * ** * * * * * * * ****

mTuba1c_NM_009448.4 gtgagtgcatctccatccacgttggccaggctggtgtccagatcggcaatgcctgctggg 595

mTuba1a_NM_011653.2 gtgagtgcatctccatccatgttggccaggctggtgtccagatcggcaatgcctgctggg 112

mTuba1b_NM_011654.2 gtgagtgcatctccatccacgttggccaggctggtgtccagatcggcaatgcctgctggg 119

******************* ****************************************

mTuba1c_NM_009448.4 agctctactgcctggaacatggcatccagcctgatggccagatgccaagtgacaagacca 655

mTuba1a_NM_011653.2 agctctactgcctggaacatggcatccagcctgatggccagatgccaagtgacaagacca 172

mTuba1b_NM_011654.2 agctctactgcctggaacatggcatccagcctgatggccagatgccaagtgacaagacca 179

************************************************************

mTuba1c_NM_009448.4 ttgggggaggagatgactccttcaacaccttcttcagtgagacaggagctggcaagcatg 715

mTuba1a_NM_011653.2 ttgggggaggagatgactccttcaacaccttcttcagtgagacaggagctggcaagcatg 232

mTuba1b_NM_011654.2 ttgggggaggagatgactccttcaacaccttcttcagtgagacaggagctggcaagcatg 239

************************************************************

mTuba1c_NM_009448.4 tgccccgggcagtgttcgtagacctggaacccacggtcatcgatgaagttcgcaccggca 775

mTuba1a_NM_011653.2 tgccccgggcagtgttcgtagacctggaacccacggtcatcgatgaagttcgcaccggca 292

mTuba1b_NM_011654.2 tgccccgggcagtgttcgtagacctggaacccacggtcatcgatgaagttcgcaccggca 299

************************************************************

mTuba1c_NM_009448.4 cctaccgccagctcttccatcctgagcagctcatcacaggcaaggaggatgctgccaata 835

mTuba1a_NM_011653.2 cctaccgccagctcttccaccctgagcagctcatcacaggcaaggaggatgctgccaata 352

mTuba1b_NM_011654.2 cctaccgccagctcttccatcctgagcagctcatcacaggcaaggaggatgctgccaata 359

******************* ****************************************

mTuba1c_NM_009448.4 actatgcccgtggccactacaccattggcaaggagatcattgaccttgtcctggacagga 895

mTuba1a_NM_011653.2 actatgctcgtggccactacaccattggcaaggagatcattgaccttgtcctggacagga 412

mTuba1b_NM_011654.2 actatgcccgtggccactacaccattggcaaggagatcattgaccttgtcctggacagga 419

******* ****************************************************

mTuba1c_NM_009448.4 ttcgcaagctggctgaccagtgcacgggtctccagggcttcttggttttccacagctttg 955

mTuba1a_NM_011653.2 ttcgcaagctggctgaccagtgcacaggtctccagggcttcttggttttccacagctttg 472

mTuba1b_NM_011654.2 ttcgcaagctggctgaccagtgcacaggtctccagggcttcttggttttccacagctttg 479

************************* **********************************

mTuba1c_NM_009448.4 gtgggggaactggctctggcttcacctccctgctgatggagcggctctctgtggattacg 1015

mTuba1a_NM_011653.2 gcgggggaactggctctggcttcacctccctgctgatggagcggctctctgtggattacg 532

mTuba1b_NM_011654.2 gtgggggaactggctctggcttcacctccctgctgatggagcggctctctgtggattacg 539

* **********************************************************

mTuba1c_NM_009448.4 gaaagaagtccaagctggagttctccatttacccagccccccaggtttccactgctgtgg 1075

mTuba1a_NM_011653.2 gaaagaagtccaagctggagttctccatttacccagccccccaggtttccactgctgtgg 592

mTuba1b_NM_011654.2 gaaagaagtccaagctggagttctccatttacccagccccccaggtttccactgctgtgg 599

************************************************************

mTuba1c_NM_009448.4 ttgagccctacaattccatcctcaccacccacaccaccctggagcactctgattgtgcct 1135

mTuba1a_NM_011653.2 ttgagccctacaattccatcctcaccacccacaccaccctggagcactctgattgtgcct 652

mTuba1b_NM_011654.2 ttgagccctacaattccatcctcaccacccacaccaccctggagcactctgattgtgcct 659

************************************************************

mTuba1c_NM_009448.4 tcatggtagacaatgaggccatctatgacatctgtcgtagaaacctcgacattgagcgcc 1195

mTuba1a_NM_011653.2 tcatggtagacaatgaggccatctatgatatctgtcgtagaaacctcgacattgagcgcc 712

mTuba1b_NM_011654.2 tcatggtagacaatgaggccatctatgacatctgtcgtagaaacctcgacattgagcgcc 719

**************************** *******************************

mTuba1c_NM_009448.4 caacctacaccaaccttaaccgccttattagccagattgtgtcttccatcactgcttccc 1255

mTuba1a_NM_011653.2 caacctacactaacctaaacaggttgataggtcaaattgtgtcttccatcactgcttccc 772

mTuba1b_NM_011654.2 caacctacaccaaccttaaccgccttattagccagattgtgtcttccatcactgcttccc 779

********** ***** *** * * ** * ** *************************

mTuba1c_NM_009448.4 tcagatttgatggggccctgaatgttgatctgacagaattccagaccaacctggtaccct 1315

mTuba1a_NM_011653.2 tcagatttgatggggccctgaatgttgatctgacagaattccagaccaacctggtaccct 832

mTuba1b_NM_011654.2 tcagatttgatggggccctgaatgttgatctgacagaattccagaccaacctggtaccct 839

************************************************************

mTuba1c_NM_009448.4 accctcgcatccacttccctctggccacttatgcccctgtcatctctgctgagaaagcct 1375

mTuba1a_NM_011653.2 accctcgtatccacttccctctggccacttatgcccctgtcatctctgctgagaaagcct 892

mTuba1b_NM_011654.2 accctcgcatccacttccctctggccacttatgcccctgtcatctctgctgagaaagcct 899

******* ****************************************************

mTuba1c_NM_009448.4 accatgagcagcttacagtagcagagatcaccaatgcctgctttgagccagccaaccaga 1435

mTuba1a_NM_011653.2 accacgagcagctttctgtagcagagatcaccaatgcctgctttgagccagccaaccaga 952

mTuba1b_NM_011654.2 accatgagcagctttctgtagcagagatcaccaatgcctgctttgagccagccaaccaga 959

**** ********* * *******************************************

mTuba1c_NM_009448.4 tggtgaaatgtgaccctcgccatggtaaatacatggcttgctgcctgctgtaccgtggtg 1495

mTuba1a_NM_011653.2 tggtgaaatgtgaccctcgccatggtaaatacatggcttgctgcctgctgtaccgtggtg 1012

mTuba1b_NM_011654.2 tggtgaaatgtgaccctcgccatggtaaatacatggcttgctgcctgctataccgtggtg 1019

************************************************* **********

mTuba1c_NM_009448.4 atgtggttcccaaagatgtcaatgctgccattgccaccatcaagaccaagcgtaccatcc 1555

mTuba1a_NM_011653.2 atgtggttcccaaagatgtcaatgctgccattgccaccatcaagaccaagcgtaccatcc 1072

mTuba1b_NM_011654.2 atgtggttcccaaagatgtcaatgctgccattgccaccatcaagaccaagcgcagcatcc 1079

**************************************************** * *****

mTuba1c_NM_009448.4 agtttgtggactggtgccccactggcttcaaggttggcattaactaccagcctcccactg 1615

mTuba1a_NM_011653.2 agtttgtggactggtgccccactggcttcaaggttggcattaactaccagcctcccactg 1132

mTuba1b_NM_011654.2 agtttgtagactggtgccccactggcttcaaggttggcattaattaccagcctcccactg 1139

******* *********************************** ****************

mTuba1c_NM_009448.4 tggtacccggtggtgacctggccaaggtgcagagagctgtgtgcatgctgagcaacacca 1675

mTuba1a_NM_011653.2 tggtacccggtggtgacctggccaaggtgcagagagctgtgtgcatgctgagcaacacca 1192

mTuba1b_NM_011654.2 tggtacccggtggtgacctggccaaggtgcagagagctgtgtgcatgctgagcaacacca 1199

************************************************************

mTuba1c_NM_009448.4 cagccattgctgaggcctgggctcgcctagatcacaagtttgatctgatgtatgccaagc 1735

mTuba1a_NM_011653.2 cagccattgctgaggcctgggctcgcctagatcacaagtttgatctgatgtatgccaagc 1252

mTuba1b_NM_011654.2 cagccattgctgaggcctgggctcgcctagatcacaagtttgatctgatgtatgccaagc 1259

************************************************************

mTuba1c_NM_009448.4 gtgcctttgtgcactggtatgtgggtgagggcatggaggagggtgagttctctgaggccc 1795

mTuba1a_NM_011653.2 gtgcctttgtgcactggtatgtgggtgagggcatggaggagggtgagttctctgaggccc 1312

mTuba1b_NM_011654.2 gtgcctttgtgcactggtatgtgggtgagggcatggaggagggtgagttctctgaggccc 1319

************************************************************

mTuba1c_NM_009448.4 gtgaggacatggctgccctagagaaggattatgaggaggttggggcagatagtgctgaag 1855

mTuba1a_NM_011653.2 gtgaggacatggctgccctagagaaggattatgaggaggttggtgtggattctgtggaag 1372

mTuba1b_NM_011654.2 gtgaggacatggctgccctagagaaggattatgaggaggttggtgtggattctgtggaag 1379

******************************************* * *** ** ****

mTuba1c_NM_009448.4 gaga------cgatgagggtgaggaatattaactcatgtgctgccat------------- 1896

mTuba1a_NM_011653.2 gcgagggggaggaagaaggagaggaatactaaattaaatgtcacaaggtgctgcttccac 1432

mTuba1b_NM_011654.2 gcgagggggaggaagaaggagaggagtactaagtccattccttgagccccctgtgtcgtc 1439

* ** ** ** ** ***** ** *** * *

mTuba1c_NM_009448.4 --------tct-------------------atacttctgtggtctcatctttgtcttt-- 1927

mTuba1a_NM_011653.2 agg--ga-tgtttattgtgttccaacacagaaagttgt--ggtctgatcagttaatttgt 1487

mTuba1b_NM_011654.2 aaactgctccagtattagttgcaggcactgatgcttct--gtgctgtttcc---a---tt 1491

* ** * * ** *

mTuba1c_NM_009448.4 --------gtgtgtgctctaact------gtcctaaactgtcaataaaggtgttt----- 1968

mTuba1a_NM_011653.2 atgtggcaatgtgtgctttcata---cagttactgacttatgaatgattgatttgacaga 1544

mTuba1b_NM_011654.2 ctgtgatcatgtcttctc-catgttgtacctcttaagttttccatgatgt----ctcaaa 1546

*** * ** * * * * * * ** *

mTuba1c_NM_009448.4 ------------------cc-------gttgtaa-------------------------- 1977

mTuba1a_NM_011653.2 gacccaagctgcccatttcacttatgggttttaaataaaatactccc------------- 1591

mTuba1b_NM_011654.2 g---------------------taaaagctttaagaaaaaaaaaaaaaaaaaaaaaaaaa 1585

* * ***

mTuba1c_NM_009448.4 ------------------------------------------------------------ 1977

mTuba1a_NM_011653.2 ------------------------------------------------------------ 1591

mTuba1b_NM_011654.2 aaaaaaaaaaaaaaaaaaaaaaaaaaaaaaaaaaaaaaaaaaaaaaaaaaaaaaaaaaaa 1645

mTuba1c_NM_009448.4 ------------------------------------------------------------ 1977

mTuba1a_NM_011653.2 ------------------------------------------------------------ 1591

mTuba1b_NM_011654.2 aaaaaaaaaaaaaaaaaaaaaaaaaaaaaaaaaaaaaaaaaaaaaaaaaaaaaaaaaaaa 1705

mTuba1c_NM_009448.4 ------------------------------------------ 1977

mTuba1a_NM_011653.2 ------------------------------------------ 1591

mTuba1b_NM_011654.2 aaaaaaaaaaaaaaaaaaaaaaaaaaaaaaaaaaaaaaaaaa 1747

**S9 Fig. CLUSTAL O(1.2.4) Multiple Sequence Alignment of *Tuba1a, Tuba1b,* and *Tuba1c* mRNA sequences.**
