# Supplementary material for: Differential requirements of tubulin genes in mammalian forebrain development
Source: PLoS Genet. 2019 Aug 6;15(8):e1008243. doi: 10.1371/journal.pgen.1008243 (PMC6697361; doi:10.1371/journal.pgen.1008243)
Supplement: S10 Fig — Analysis of Tubb2a and Tubb2b genomic DNA (A,C) and cDNA (B,D) in Tubb2a and Tubb2b mutants. (DOCX) [file pgen.1008243.s010.docx]

*Tubb2a* cDNA in *Tubb2a* deletion mutants.

(A) We used previously published [37] primers (*Tubb2a* F: AACCAGATCGGCGCTAAGT; *Tubb2a* R: TCCAGCTGCAAGTCACTGTC). We performed multiple PCR reactions with genomic DNA (Platinum High Fidelity Taq at 58^o^ C with a 2:00 extension time) from control and homozygous deletion animals and were able to amplify a product approximately 1,400 bp in size. Sanger sequencing of this reaction indicates this is indeed wild-type *Tubb2a* genomic sequence. In multiple, but not all, experiments, we only amplify a sequence greater than 3,000 bp in deletion mutant DNA, which has been refractory to quality Sanger sequencing. We suspect this product represents a non-desired target sequence to which the PCR primers are only able to hybridize with in the absence of the preferred *Tubb2a* sequence deleted in the CRISPR-CAS9 alleles.

D4222/D4222


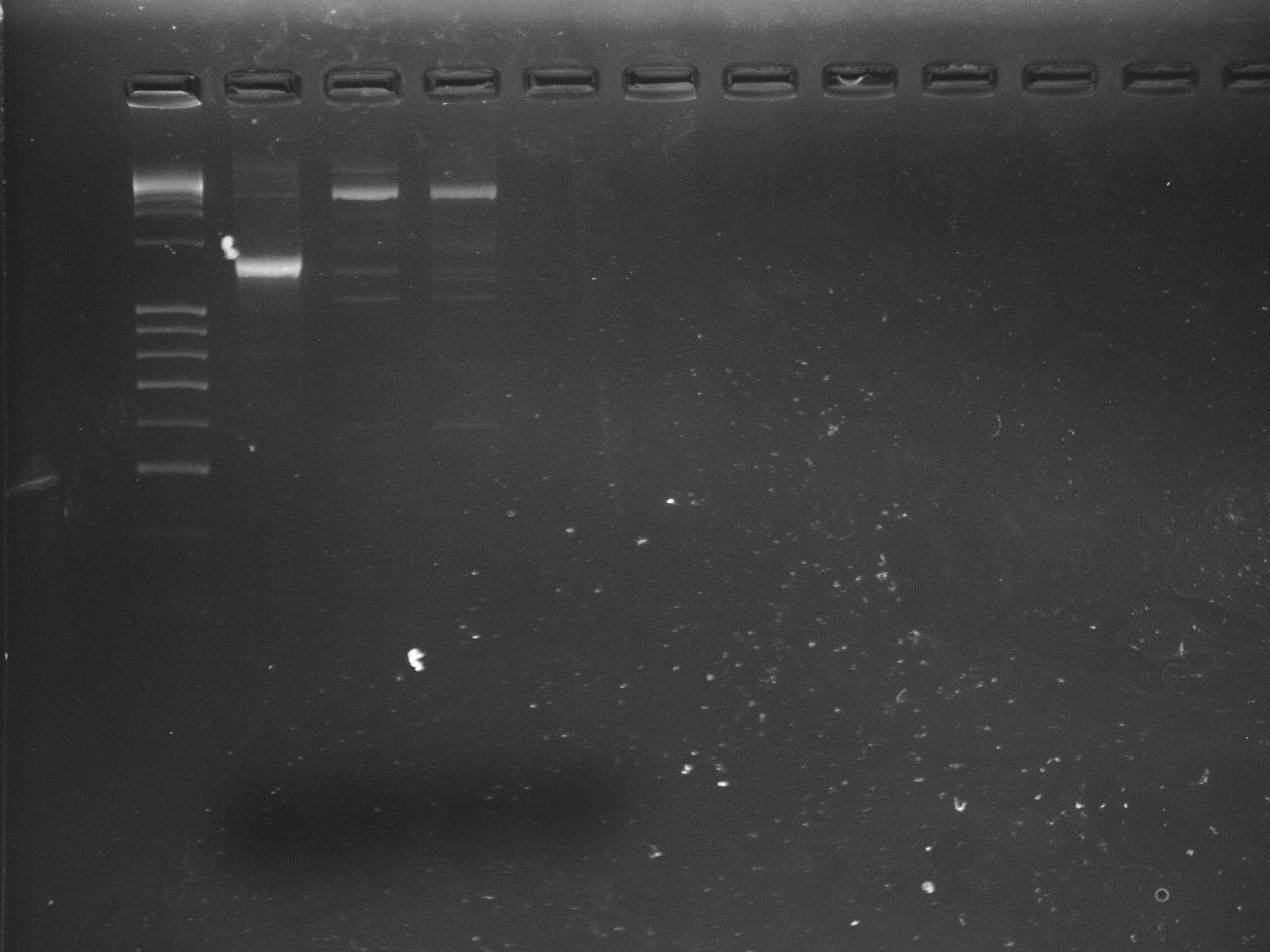


**S10 Fig. (A)** *Tubb2a* Primers Platinum Taq HiFi 1:50 Genomic DNA

wild-type mutant mutant neg control

+/+

D3963/D3963

1000bp

1500bp

(B) We also performed PCR with the same primers on cDNA isolated from wild-type and homozygous deletion mutants. We were able to amplify the expected 95 bp product from wild-type as well as both deletion mutants. These short products are not ideal for Sanger sequencing short of sub-cloning into plasmids to overcome initial poor quality sequencing at the ends of an amplicon. In performing alignments of the sequences we do retrieve, amplicons from mutant and wild-type can align to both *Tubb2a* as well as exon 4 of 4930447K03Rik. Our interpretation of this data is that the cDNA product from the *Tubb2a* deletion samples is actually from a highly similar region of the genome which is likely to be transcribed, at least at some low level.

500bp


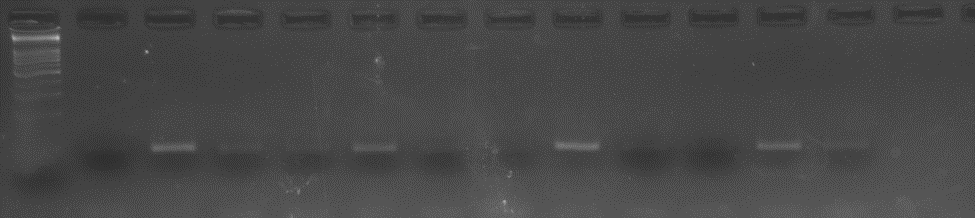


wild-type neg. mutant neg. wild-type neg. mutant neg.

Wt gDNA

Wt cDNA

mut gDNA

mut cDNA

**S10 Fig. (B)** *Tubb2a* Primers Platinum Taq HiFi on 1:50 genomic DNA and cDNA

mut cDNA

mut gDNA

Wt cDNA

Wt gDNA

*Tubb2b* cDNA in *Tubb2b* deletion mutants.

(C) We again used published primers [37] (*Tubb2b* F: TCATCAGACCCACTGACACAG *Tubb2bR*: TTTCCAGTTGCAAATCACTGTC). We first noted the reverse primer aligns to *Tubb2b* sequence but also to *Tubb2a-ps2*. A PCR reaction with the same conditions as above amplifies the expected product in wild-type animals, but not mutants.


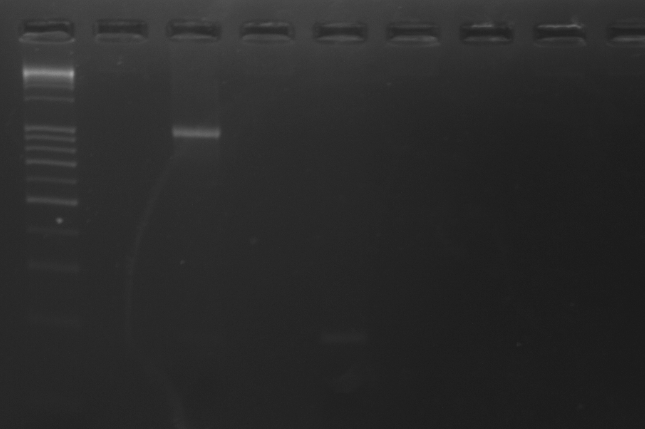


Blank wild-type Blank mutant Blank neg.

control

+/+

D4185/D4185

**S10 Fig. (C)** *Tubb2b* Primers Platinum Taq HiFi 1:50 Genomic DNA

1000bp

(D) PCR on cDNA from wild-types and mutants generates a product of the predicted size in both wild-type and deletion mutants. Sanger sequencing of these products aligned perfectly to *Tubb2b*. It also aligns very well (98.4%; 175/178 nucleotides) with the *Tubb2a-ps2*. We therefore conclude that these highly homologous sequences confound this analysis, but our genomic DNA analysis is consistent with deletions as reported.


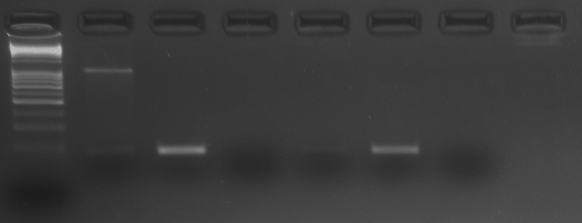


wild-type neg. control mutant neg. control

Wt gDNA

Wt cDNA

mut gDNA

mut cDNA

**S10 Fig. (D):** *Tubb2b* Primers Platinum Taq HiFi 1:50 genomic DNA and cDNA
