# Supplementary material for: Differential requirements of tubulin genes in mammalian forebrain development
Source: PLoS Genet. 2019 Aug 6;15(8):e1008243. doi: 10.1371/journal.pgen.1008243 (PMC6697361; doi:10.1371/journal.pgen.1008243)
Supplement: S11 Fig — (DOCX) [file pgen.1008243.s011.docx]

mTubb2a ---GGTCTTTTGCGC------TCCTTAG------CCCTCTGTCCACGCACTG--------

mTubb2b probe CTCAGCCCGTAGCCCGTCGGTTCCGGAGTAAGTTCCAGGTGGCCCAGCAGTGGGTGTGGA

.* * *:** * *** ** **. ** **..*** **

mTubb2a ----------CTCCGAGGGCAAGAGCCTCCACCCCTT--CTACAA--------CCAG-CA

mTubb2b probe AGGGGAGGATCATC-AGACCCACTGACACAGACCCAAGACAGCAAGAAGCTAACCAGGCA

*: * **. *.* :*.*:*...***:: *:.*** **** **

mTubb2a CCATGCGCGAGATCGTGCACATCCAGGCGGGCCAGTGTGGCAACCAGATCGGCGCTAAG

mTubb2b probe CCATGCGAGAGATCGTGCACATTCAGGCGGGCCAGTGCGGCAACCAGATCGGTGCCAAG

*******.************** ************** ************** ** ***

**S11 FIG*.*** ***Tubb2b* mRNA in situ probe sequence from Jaglin et al., and homologous *Tubb2a* sequence**
