# Supplementary material for: Differential requirements of tubulin genes in mammalian forebrain development
Source: PLoS Genet. 2019 Aug 6;15(8):e1008243. doi: 10.1371/journal.pgen.1008243 (PMC6697361; doi:10.1371/journal.pgen.1008243)
Supplement: S12 Fig — (DOCX) [file pgen.1008243.s012.docx]

Cow TUBB EGMDEMEFTEAESNMNDLVSEYQQYQDATAEEEED

Mouse TUBB3 EGMDEMEFTEAESNMNDLVSEYQQYQDATAEEEGE

Mouse TUBB2A EGMDEMEFTEAESNMNDLVSEYQQYQDATADEQGE

Mouse TUBB2B EGMDEMEFTEAESNMNDLVSEYQQYQDATADEQGE

Mouse TUBB5 EGMDEMEFTEAESNMNDLVSEYQQYQDATAEEEED

Mouse TUBB4A EGMDEMEFTEAESNMNDLVSEYQQYQDATAEEGEF

Mouse TUBB4B EGMDEMEFTEAESNMNDLVSEYQQYQDATAEEEGE

******************

**S12 Fig. Alignment of amino acids 400-434 of cow TUBB with 6 mouse β-tublin genes.** Epitope for pan- β−tubulin antibody is underlined. Asterisks indicate identity between epitope sequence and mouse TUBB sequences.
