# Supplementary material for: Differential requirements of tubulin genes in mammalian forebrain development
Source: PLoS Genet. 2019 Aug 6;15(8):e1008243. doi: 10.1371/journal.pgen.1008243 (PMC6697361; doi:10.1371/journal.pgen.1008243)
Supplement: S13 Fig — (DOCX) [file pgen.1008243.s013.docx]

Human TUBA1A AALEKDYEEVGVDSVEGEGEEEGEE

Mouse TUBA1A AALEKDYEEVGVDSVEGEGEEEGEE

Mouse TUBA1B AALEKDYEEVGVDSVEGEGEEEGEE

Mouse TUBA1C AALEKDYEEVGADSAEGDDEGEE

Mouse TUBA3A AALEKDYEEVGVDSVEAEAEEGEE

Mouse TUBA3B AALEKDYEEVGVDSVEAEAEEGEE

Mouse TUBA4A AALEKDYEEVGIDSYEDEDEGEE

Mouse TUBA8 AALEKDYEEVGTDSFEEENEGEE

*********** ** * *

**S13 Fig. Alignment of amino acids 426-450 of human TUBA1A sequence with seven mouse α-tubulin genes.** This sequence is reported to be the epitope used for the DMIA anti-α-tubulin clone. Asterisks indicate identity between epitope sequence and mouse TUBA sequences.
