# Supplementary material for: Differential requirements of tubulin genes in mammalian forebrain development
Source: PLoS Genet. 2019 Aug 6;15(8):e1008243. doi: 10.1371/journal.pgen.1008243 (PMC6697361; doi:10.1371/journal.pgen.1008243)
Supplement: S1 Table — (DOCX) [file pgen.1008243.s014.docx]

**S1 Table.** CRISPR guide and PCR primers sequences.

| **CRISPR Guides** | **Sequence** |  |
| --- | --- | --- |
| *Tuba1a* 5’UTR #1 | CAAAGTCTACGGATGCTAGG |  |
| *Tuba1a* 5’UTR #2 | AAAAGTAGCAGAAGATACGG |  |
| *Tuba1a* 3’UTR #1 | AATAAATACCACGACCTCAG |  |
| *Tuba1a* 3’UTR #2 | TACTGTCTGTCCATCAAGCG |  |
| *Tubb2a* 5’UTR #1 | GCAGGACTTGAACTGCAGCC |  |
| *Tubb2a* 5’UTR #2 | GGGGGCGTAATAACCCTAGG |  |
| *Tubb2a* 3’UTR #1 | ACTAAGCAGAAGTCCCATGA |  |
| *Tubb2a* 3’UTR #2 | TCTGAAATAGAAACCATCAT |  |
| *Tubb2b* 5’UTR #1 | GGGCTTGTGGCCAATCAGCG |  |
| *Tubb2b* 5’UTR #2 | ACGTAATGCTCTGAGCCCCA |  |
| *Tubb2b* 3’UTR #1 | TTAGGGGTCCAGTCACCTAT |  |
| *Tubb2b* 3’UTR #2 | GATAATGCTATTCTTCAGGG |  |
|  |  |  |
| **PCR/Sequencing Primers (numbers as used in S2 Fig).** |  |  |
| *Tuba1a* Deletion F* (p1144) | ATTAGTGTGGGAAGGTTGGGTC |  |
| *Tuba1a* Deletion R (p1145) | ACTAAGGCCAGTTAAGATGACCT |  |
| *Tuba1a* Deletion F #2 (p1146) | TCTTTGGGAGCATCATTTTCTTCA |  |
| *Tuba1a* Deletion R #2 (p1147) | CTGCAAGTCTTCCTGCCTCT |  |
| *Tuba1a* Deletion F #3 (p1142) | TTTCTTTCTTCCCTTTCCACCC |  |
| *Tuba1a* Deletion R #3 (p1367) | GTCTTCCTGCCTCTGACTCC |  |
| *Tuba1a* WT Left F (p1364) | GCCTCCATCACACTAGTCTGT |  |
| *Tuba1a* WT Left R (p1365) | TTGACAGAGACCCAAGCTGC |  |
| *Tuba1a* WT Left #2 R (p1230) | TTGAGTGTGTGCCAGCTTTC |  |
| *Tuba1a* WT Right F (p658) | ATGCGGCTCCTTATATACAGCT |  |
| *Tuba1a* WT Right R (p659) | CCCCGTATCTTCTGCTACTTTT |  |
|  |  |  |
| *Tubb2a* Deletion F* (p664) | TGGGAAACATTTGTAGTGTTCCT |  |
| *Tubb2a* Deletion R (p663) | ATTAGCTACCTCCACTTCCCTC |  |
| *Tubb2a* Deletion #2 F (p1168) | TCTTCCCTGCAATGTGACTCTA |  |
| *Tubb2a* Deletion #3 F (p1166) | GTGTTCCTGTTTGCTATTTTAGGAC |  |
| *Tubb2a* Deletion #3 R (p725) | TAAGAGGCTTTTAGGACCCAAG |  |
| *Tubb2a* WT Left #1 R (p1369) | AATTATGGTGCTCAGTTTGCC |  |
| *Tubb2a* WT Left #2 R (p1371) | ATGGTGCTCAGTTTGCCTCT |  |
| *Tubb2a* Intron 1 F (p1182) | CCTGAAAGCCGACCAACTTG |  |
| *Tubb2a* Intron 1 R (p1183) | CCCAAAGACACGCCTCTGTA |  |
| *Tubb2b* Deletion F (p1170) | GCCCTCCTTTTAAAGCCGTG |  |
| *Tubb2b* Deletion R (p1171) | ACTCAGCTTGCCCTTCTCAA |  |
| *Tubb2b* Deletion #2 F (p668) | TTATGCCCTCCTTTTAAAGCCG |  |
| *Tubb2b* Deletion #2 R (p780) | AAAGCTCCCTACCAGTGCTC |  |
| *Tubb2b* WT Left F (p781) | TGCTCATACTTCTCCTTGCAAC |  |
| *Tubb2b* WT Left R (p782) | CCACAGCATCCACAGTTTTACA |  |
| *Tubb2b* WT Left #2 R (p669) | CATCACTGTGTTCAAGGAGCAG |  |
| *Tubb2b* WT Right F (p666) | TCGGTCTTTTATGTAGGGAGGG |  |
| *Tubb2b* WT Right R (p667) | AGAGGAATTGCTCAAGTAACGC |  |
| *Tubb2b* Deletion Sequencing (p1794)* | AAGCTCCCTACCAGTGCTCA |  |
| *Tubb2b* Intron 1 F (p1186) | GCGCGGCTCTAGGTAAATAC |  |
| *Tubb2b* Intron 1 R (p1187) | CGAGGAAGCTGTAGGGTGAT |  |
| *Tubb2b* Intron 2 F (p1961) | GTCCCACCTCCTTCCTGTTT |  |
| *Tubb2b* Intron 2 R (p1962) | TCTGGCATCCCTAAGCTCTG |  |
| *Tubb2b* Intron 3 F (p1963) | GCAAAGGAGGATTAAGTCAGCC |  |
| *Tubb2b* Intron 3 R (p1964) | GGGAGCATACATAGCAGTGG |  |
| *Tuba1a quasimodo F ** | ACTGGATGGTACGCTTGGTC |  |
| *Tuba1a quasimodo R* | TGGGAGGGTGTCTTGGTATC |  |
| * Primers used for sequencing | | |
|  | | |
| **Primers used for *Tubb2a*/ *Tubb2b* cDNA analysis (S10 Fig)** | | |
| *Tubb2a F* | AACCAGATCGGCGCTAAGT | |
| *Tubb2a R* | TCCAGCTGCAAGTCACTGTC | |
| *Tubb2b F* | TCATCAGACCCACTGACACAG | |
| *Tubb2b R* | TTTCCAGTTGCAAATCACTGTC | |
