# Supplementary material for: Differential requirements of tubulin genes in mammalian forebrain development
Source: PLoS Genet. 2019 Aug 6;15(8):e1008243. doi: 10.1371/journal.pgen.1008243 (PMC6697361; doi:10.1371/journal.pgen.1008243)
Supplement: S2 Table — (DOCX) [file pgen.1008243.s015.docx]

**S2 Table**. Predicted “off-target” sites for CRISPR guides.

*Tuba1a* 3’UTR #1 Guide: AATAAATACCACGACCTCAG AGG chr 15: 98,949,762

| **Off-target Sequence** | **Score** | **chr Location** |
| --- | --- | --- |
| GGTGAATGCCACGACCTCAG | 1.5 | chr7:-83491819 |
| AAGCAATAGGACGACCTCAG | 0.8 | chr3:-23708176 |
| AAGCAATAGGACGACCTCAG | 0.8 | chr3:-23707556 |
| TACAAATGCCAGGACCTCAG | 0.8 | chr17:+28297383 |
| TAAAGATACCAAGACCTCAG | 0.8 | chr17:-8683891 |
| AATATATACCATGACCTCAA | 0.8 | chr4:-90446340 |
| AATAAAGACAACCACCTCAG | 0.7 | chr18:-15171401 |
| TATAAACACAACGACCTCAA | 0.6 | chr1:+150828417 |
| AATCAAGACAGCGACCTCAG | 0.5 | chr2:-39161508 |
| AAATAATAGCATGACCTCAG | 0.5 | chr12:-28982200 |

*Tuba1a* 3’UTR #2 Guide: TACTGTCTGTCCATCAAGCG GGG chr15: 98,949,721

| **Off-target Sequence** | **Score** | **chr Location** |
| --- | --- | --- |
| GGCTGTCTGTCCATCAAGCG | 5.2 | chr11:+52012531 |
| TACGGTCTGTCCATCAAGTG | 4.3 | chr1:+79635089 |
| TGCTGTCTGTCCCTCAAGCG | 3.6 | chr10:+75006034 |
| CCCTGTCTGTCCATCAAGCA | 2.0 | chr8:+28345267 |
| TAATGTCTCCCCATCAAGCG | 1.5 | chr15:-50405161 (48.5 Mb, 24cM) |
| TACTGACAGTCCATCAAGAG | 0.7 | chr1:+33001934 |
| AAATCTCTGTCCATCAAGTG | 0.6 | chr3:+117843514 |
| TGCAATCTGTCCATCAAGAG | 0.6 | chrX:-106031348 |
| TACTTTCTGTCCATCAGGCC | 0.6 | chr13:-71553485 |
| TACAGCCGCTCCATCAAGCG | 0.5 | chr17:+28313317 |

*Tuba1a* 5’UTR #1 Guide: CAAAGTCTACGGATGCTAGG GGG chr15: 98,953,719

| **Off-target Sequence** | **Score** | **chr Location** |
| --- | --- | --- |
| CCAATTCTAGGGATGCTAGG | 2.6 | chr5:+102121981 |
| AAGAGTCAAAGGATGCTAGG | 1.5 | chr13:-92435618 |
| CCAGGTCAAAGGATGCTAGG | 1.4 | chr4:+138930030 |
| CTACTTCTAGGGATGCTAG | 1.4 | chr3:+88535620 |
| CAGCATCTAAGGATGCTAGG | 1.3 | chr1:-180991328 |
| CAAAGTCAGAGGATGCTAGG | 1.3 | chr2:-148497404 |
| TAAAGTCCACGGGTGCTAGG | 1.3 | chr5:+74299424 |
| AAAATTCAACGGATGCTAGA | 0.9 | chrX:+153695867 |
| TTAAGTCTATGGATGCTAGA | 0.9 | chr4:-43878776 |
| AAAAGCCCAAGGATGCTAGG | 0.9 | chrX:-103725186 |

*Tuba1a* 5’UTR #2 Guide: AAAAGTAGCAGAAGATACGG GGG chr15: 98,954,015

| **Off-target Sequence** | **Score** | **chr Location** |
| --- | --- | --- |
| AACAGTAGCCAAAGATACGG | 1.4 | chr8:-117896517 |
| GAAAGTAGTAGTAGATACGG | 1.0 | chr2:+5834051 |
| TAAATTAATAGAAGATACGG | 0.9 | chr3:+21092061 |
| AAGACTGGCTGAAGATACGG | 0.9 | chr12:+107049314 |
| GAAGGTATCAAAAGATACGG | 0.9 | chr9:+21060983 |
| ACAATTAGATGAAGATACGG | 0.9 | chr14:+63465798 |
| AAAGCTACAAGAAGATACGG | 0.9 | chr4:-87865811 |
| AGATATAGCAAAAGATACGG | 0.9 | chr16:+38421482 |
| AAACAAAACAGAAGATACGG | 0.8 | chr8:-105729509 |
| ACAATTAGCAGAAGATAGGG | 0.8 | chr19:-39016294 |

*Tubb2a* 3’UTR Guide: ACTAAGCAGAAGTCCCATGA TGG chr13: 34,074,222

| **Off-target Sequence** | **Score** | **Chr. Location** |
| --- | --- | --- |
| ACCAAGCAGAATTCCCATGA | 3.9 | chr12:+16278857 |
| CCTACACAGAAGTCCCATGA | 1.6 | chr2:-46918038 |
| ACCAAGCATCAGTCCCATGA | 1.5 | chr12:+76813195 |
| TCCCAGCAGGAGTCCCATGA | 1.4 | chr10:-83661130 |
| GGAGAGCAGAAGTCCCATGA | 1.3 | chr17:-46028356 |
| ACTGAGCAGCAGTCCCTTGA | 1.2 | chr15:+96526850 |
| AAGAAGCCAAAGTCCCATGA | 0.9 | chr14:-28752693 |
| ACCAAGCACAACTCCCATGA | 0.9 | chr11:-37260418 |
| AGTAAGCAGCAGTCACATGA | 0.9 | chr3:-84886560 |
| GCTGAGCAGAAGTCCCAGGA | 0.8 | chr5:+30641736 |

*Tubb2a* 3’UTR #2 Guide: TCTGAAATAGAAACCATCAT GGG chr13: 34,074,209

| **Off-target Sequence** | **Score** | **chr. Location** |
| --- | --- | --- |
| TATAAAATAGAAACCATCAT | 5.5 | chr18:+79031786 |
| CCTAGAATAGAAACCATCAT | 2.5 | chr1:-47390943 |
| TGTCCAATAGAAACCATCAT | 2.4 | chr2:-178489185 |
| TCTGAAGTAGGAACCATCAT | 2.3 | chr18:+31521803 |
| ACAGAAATAGAAACCATCAA | 2.0 | chr9:-46277492 |
| TATGAAAAAGAAACCATCAA | 1.9 | chr19:+24421900 |
| TCTGAAATAGCAACCATCAA | 1.9 | chr1:+33215086 |
| ACTGAAATAAAAACCATCAA | 1.8 | chr7:+116087698 |
| TCAGAAATATAAACCATCAA | 1.6 | chr9:-31177639 |
| TTTTAAATAGAAACCAGCAT | 1.5 | chrX:-112897669 |

*Tubb2a* 5’UTR Guide: GCAGGACTTGAACTGCAGCC CGG chr13: 34,078,431

| **Off-target Sequence** | **Score** | **chr Location** |
| --- | --- | --- |
| TCAGAACTAGAACTGCAGCC | 1.8 | chr15:+74175139 |
| GCAATACTTGAACTGCAGCT | 1.7 | chr17:-74645412 |
| TGAGAACTTTAACTGCAGCC | 1.5 | chr3:-25131926 |
| CCAGGACTTCAGCTGCAGCC | 1.5 | chr5:+128435954 |
| CCAGGACTTCATCTGCAGCC | 1.5 | chr2:-35045018 |
| GCAGTACTCAAACTGCAGCC | 1.5 | chrX:+159996039 |
| AAATGACTTAAACTGCAGCC | 1.4 | chr6:-70836207 |
| GGAGCACTTGAACTGCAGTC | 1.3 | chr4:-44911742 |
| GATACACTTGAACTGCAGCC | 1.3 | chr1:-185284971 |
| GCAGGACCTCACCTGCAGCC | 1.1 | chr7:+145207308 |

*Tubb2a* 5’UTR #2 Guide: GGGGGCGTAATAACCCTAGG AGG chr13: 34,078,164

| **Off-target Sequence** | **Score** | **chr Location** |
| --- | --- | --- |
| TGGTGGGGAATAACCCTAGG | 0.9 | chr8:+108102731 |
| GGGAGCTTAATAACCCTATG | 0.8 | chr10:-86707885 |
| AGTGGCGAAATAACCCAAGG | 0.8 | chr9:+45519805 |
| AGGGGCGAAGTAACCCCAGG | 0.7 | chr3:-131107876 |
| GGGGGAATAAAAACCCTAGG | 0.6 | chr12:+106716037 |
| TGGAGTGTAAGAACCCTAGG | 0.5 | chr2:+17930065 |
| GGGGCCGTGATAACACTAGG | 0.5 | chr19:-8666554 |
| TGGAGGGTAATTACCCTAGG | 0.5 | chr10:+115907895 |
| GGGGTTGTATTTACCCTAGG | 0.4 | chr3:-104388965 |
| GGTGGCTTAATAAACCTAGG | 0.3 | chr7:+116657947 |

*Tubb2b* 3’UTR Guide: TTAGGGGTCCAGTCACCTAT AGG chr13: 34,126,379

| **Off-target Sequence** | **Score** | **chr Location** |
| --- | --- | --- |
| TTAGGGGTGCAGTCACCTGT | 1.7 | chr14:+58182357 |
| AGTGGGGTCCAGTCACCTAA | 0.9 | chr5:-66462898 |
| TATGGGGTCAAATCACCTAT | 0.8 | chr5:-33659521 |
| TTAGGTGACCAGTCACCTGT | 0.7 | chr8:-20939427 |
| AGAGAGGTCCAGTCACCTCT | 0.7 | chr16:+48430361 |
| TTTGGGGGCAAGTCACTTAT | 0.6 | chr7:-97332928 |
| TAAGGGTCCCTGTCACCTAT | 0.6 | chrX:+143201526 |
| CTAGGGTTCAAGTCACCTAG | 0.6 | chr4:+114421550 |
| GTAGGTGTATAGTCACCTAT | 0.5 | chr11:+6426255 |
| TAAGGGGTCTTGTCACCTAG | 0.4 | chr9:-111941960 |

*Tubb2b* 3’UTR #2 Guide: GATAATGCTATTCTTCAGGG AGG chr13: 34,126,820

| **Off-target Sequence** | **Score** | **chr Location** |
| --- | --- | --- |
| GGCAATGCTCTTCTTCAGGG | 2.6 | chr2:-76575119 |
| GGTCATGCCATTCTTCAGGG | 1.7 | chr16:+85596080 |
| GATTTAGCTATTCTTCAGGG | 1.4 | chr1:-60517082 |
| GAAGTTGCTTTTCTTCAGGG | 1.3 | chr5:-8289838 |
| GAAAATGCTTTGCTTCAGGG | 1.3 | chr6:-20206144 |
| AAAAATCCTCTTCTTCAGGG | 1.0 | chr9:+89722526 |
| TATAATGCAATCCTTCAGGG | 1.0 | chr4:-144364304 |
| TATAATGCAATCCTTCAGGG | 1.0 | chr4:+143964244 |
| TATAATGCAATCCTTCAGGG | 1.0 | chr4:+143948301 |
| TATAATGCAATCCTTCAGGG | 1.0 | chr4:+143928066 |

*Tubb2b* 5’UTR Guide: GGGCTTGTGGCCAATCAGCG CGG chr13: 34,130,518

| **Off-target Sequence** | **Score** | **chr Location** |
| --- | --- | --- |
| TAGCCTGTGGCCAATCAGTG | 0.7 | chr9:+44113549 |
| AGGCCTGTGTCCAATCAGAG | 0.6 | chr6:-86602209 |
| AGCCTTCTGGCCAATCAGCA | 0.6 | chr15:-89568297 |
| GAGCTTGCAGCCAATCAGCT | 0.5 | chr2:-91962428 |
| AGGCTTGGGGACAATCAGCT | 0.5 | chr18:+71679479 |
| AGGCCTGTGGCCAATCTGCC | 0.4 | chr8:+118603271 |
| GTGGGTGTGGCCACTCAGCG | 0.3 | chr8:-92522325 |
| GGTCATGTGGCCCATCAGGG | 0.2 | chr9:+102674627 |
| TGGCTGGTTGCCAATCAGAG | 0.2 | chr7:+41498686 |
| GGCCTTCTGGGCTATCAGCG | 0.2 | chr11:-120017382 |

*Tubb2b* 5’UTR #2 Guide: ACGTAATGCTCTGAGCCCCA GGG chr13: 34,130,518

| **Off-target Sequence** | **Score** | **chr Location** |
| --- | --- | --- |
| AAGGAATGCTCGGAGCCCCA | 1.5 | chr2:-158230556 |
| TCCTCATTCTCTGAGCCCCA | 1.5 | chr14:+58015539 |
| TCTTAATCCCCTGAGCCCCA | 1.5 | chr3:+100022952 |
| CCAGAATGCCCTGAGCCCCA | 1.4 | chr11:+82684143 |
| CCACCATGCTCTGAGCCCCA | 1.4 | chr8:+126566745 |
| AAAGGATGCTCTGAGCCCCA | 1.3 | chr5:+38497227 |
| CACAAATGCTCTGAGCCCCA | 1.3 | chr2:+153505685 |
| AAGTAATGCCCTAAGCCCCA | 1.1 | chr7:+88118409 |
| CTGTAAGACTCTGAGCCCCA | 1.0 | chr5:-125124336 |
| ACTCAACTCTCTGAGCCCCA | 1.0 | chr11:-47677497 |

Top 10 predicted off-target sites (Benchling.com) are shown for each guide. Sequences on the same chromosome are shown and intervening distance in physical coordinates and cM is indicated (based on average value of 2Mb = 1 cM as defined in *Mouse Genetics* Lee Silver 1995).
