# Supplementary material for: Differential requirements of tubulin genes in mammalian forebrain development
Source: PLoS Genet. 2019 Aug 6;15(8):e1008243. doi: 10.1371/journal.pgen.1008243 (PMC6697361; doi:10.1371/journal.pgen.1008243)
Supplement: S3 Table — (DOCX) [file pgen.1008243.s016.docx]

**S3 Table**. *Tubb2a* and *Tubb2b* deletion allele statistical analysis.

|  | **ANOVA F statistic**  **(P value)** |  | **Tukey’s multiple comparison adjusted P value** | **Mean difference**  increase  decrease |  |
| --- | --- | --- | --- | --- | --- |
| **Body weight** | | | | | |
| *Tubb2a* d3963 | 0.209 (0.812) |  |  |  |  |
| *Tubb2b* d4222 | 0.141 (0.869) |  |  |  |  |
| *Tubb2b* d4185 | 0.485 (0.619) |  |  |  |  |
| **Brain weight** | | | | | |
| *Tubb2a* d3963 | 1.817 (0.201) |  |  |  |  |
| *Tubb2b* d4222 | 0.040 (0.961) |  |  |  |  |
| *Tubb2b* d4185 | 0.808 (0.464) |  |  |  |  |
| **Motor Cortex - Cell Densities** | | | | | |
| *Tubb2a* d3963 | 7.216 (0.0024) | wt vs. *Tubb2a* d3963/wt | 0.306 | 3.7% increase |  |
|  |  | *Tubb2a* d3963/wt vs. *Tubb2a* d3963/d3963 | 0.002 | 11.1% decrease |  |
|  |  | wt vs. *Tubb2a* d3963/d3963 | 0.056 | 7.4% decrease |  |
| *Tubb2a* d4222 | 2.662 (0.081) | wt vs. *Tubb2b* d4222/wt | 0.070 | 10.0% decrease |  |
|  |  | *Tubb2b* d4222/wt vs. *Tubb2b* d4222/d4222 | 0.702 | 4.45% increase |  |
|  |  | wt vs. *Tubb2b* d4222/d4222 | 0.322 | 6.4% decrease |  |
| *Tubb2b* d4185 | 5.216 (0.008) | wt vs.  *Tubb2b* d4185/wt | 0.024 | 14.8% decrease |  |
|  |  | *Tubb2b* d4185/wt vs.  *Tubb2b* d4185/ d4185 | 0.861 | 2.8% decrease |  |
|  |  | wt vs.  *Tubb2b* d4185/ d4185 | 0.011 | 17.7% decrease |  |
| **Somatosensory Cortex - Cell Densities** | | | | |  |
| *Tubb2a* d3963 | 2.755 (0.078) | wt vs. *Tubb2a* d3963/wt | 0.486 | 2.6% increase |  |
|  |  | *Tubb2a* d3963/wt vs. *Tubb2a* d3963/d3963 | 0.067 | 6.7% decrease |  |
|  |  | wt vs. *Tubb2a* d3963/d3963 | 0.399 | 2.9% decrease |  |
| *Tubb2a* d4222 | 4.515 (0.016) | wt vs. *Tubb2b* d4222/wt | 0.020 | 13.1% decrease |  |
|  |  | *Tubb2b* d4222/wt vs. *Tubb2b* d4222/d4222 | 0.829 | 2.8% increase |  |
|  |  | wt vs. *Tubb2b* d4222/d4222 | 0.074 | 10.3% decrease |  |
| *Tubb2b* d4185 | 6.200 (0.004) | wt vs.  *Tubb2b* d4185/wt | 0.069 | 4.7% decrease |  |
|  |  | *Tubb2b* d4185/wt vs.  *Tubb2b* d4185/ d4185 | 0.248 | 3.0% decrease |  |
|  |  | wt vs.  *Tubb2b* d4185/ d4185 | 0.003 | 7.8% decrease |  |
