# Supplementary material for: Differential requirements of tubulin genes in mammalian forebrain development
Source: PLoS Genet. 2019 Aug 6;15(8):e1008243. doi: 10.1371/journal.pgen.1008243 (PMC6697361; doi:10.1371/journal.pgen.1008243)
Supplement: S4 Table — (DOCX) [file pgen.1008243.s017.docx]

**S4 Table.** Statistical analysis of immunohistochemistry for layer markers in *Tubb2a* and *Tubb2b* deletion mutants.

|  | | | | **T-test p value** | | **Mean difference**  Increase. decrease | |
| --- | --- | --- | --- | --- | --- | --- | --- |
| **CTIP2** | Wt vs. *Tubb2a* *d3963/d3963* | Bin1 |  | |  | |  |
|  |  | Bin2 | 0.4613 | | 11 % increase | |  |
|  |  | Bin3 | 0.4613 | | 13.4 % decrease | |  |
|  | Wt vs. *Tubb2a d4222/d4222* | Bin1 |  | |  | |  |
|  |  | Bin2 | 0.0557 | | 42% increase | |  |
|  |  | Bin3 | 0.0557 | | 25% decrease | |  |
|  | Wt. vs*. Tubb2b d4185/4185* | Bin1 |  | |  | |  |
|  |  | Bin2 | 0.64 | | 8.9% increase | |  |
|  |  | Bin3 | 0.64 | | 6% decrease | |  |
| **TBR1 DMC** | Wt vs. *Tubb2a* *d3963/d3963* | Bin1 | 0.0007 | | 70.8% increase | |  |
|  |  | Bin2 | <0.0001 | | 33% decrease | |  |
|  |  | Bin3 | 0.0003 | | 95% increase | |  |
|  | Wt vs. *Tubb2a d4222/d4222* | Bin1 | 0.342 | | 19% increase | |  |
|  |  | Bin2 | 0.024 | | 11.6 % decrease | |  |
|  |  | Bin3 | 0.067 | | 73.4% increase | |  |
|  | Wt. vs*. Tubb2b d4185/4185* | Bin1 | 0.067 | | 32% increase | |  |
|  |  | Bin2 | 0.0040 | | 20% decrease | |  |
|  |  | Bin3 | 0.0054 | | 70% increase | |  |
| **TBR1 PC** | Wt vs. *Tubb2a* *d3963/d3963* | Bin1 | <0.0001 | | 41.7% increase | |  |
|  |  | Bin2 | <0.0001 | | 52% decrease | |  |
|  |  | Bin3 | <0.0001 | | 133% increase | |  |
|  | Wt vs. *Tubb2a d4222/d4222* | Bin1 | 0.8 | | 6% decrease | |  |
|  |  | Bin2 | 0.0002 | | 57% decrease | |  |
|  |  | Bin3 | 0.95 | | 2%decrease | |  |
|  | Wt. vs*. Tubb2b d4185/4185* | Bin1 | 0.76 | | 6% decrease | |  |
|  |  | Bin2 | 0.66 | | 4% increase | |  |
|  |  | Bin3 | 0.6 | | 15.9% decrease | |  |
