# Supplementary material for: Differential requirements of tubulin genes in mammalian forebrain development
Source: PLoS Genet. 2019 Aug 6;15(8):e1008243. doi: 10.1371/journal.pgen.1008243 (PMC6697361; doi:10.1371/journal.pgen.1008243)
Supplement: S5 Table — (DOCX) [file pgen.1008243.s018.docx]

**S5 Table.** Statistical analysis of immunoblotting for total beta-tubulin and TUBB3 in *Tubb2a* and *Tubb2b* deletion alleles.

|  | **ANOVA F statistic (p value)** |  | **Tukey’s multiple comparison adjusted P value** | **Mean difference**  increase  decrease |
| --- | --- | --- | --- | --- |
| *Tubb2a* D3963 | 1.891 (0.185) |  | N/A |  |
| *Tubb2a* D4222 | 3.800 (0.046) | wt vs. d4222/wt | 0.175 | 22.1% increase |
|  |  | wt vs. d4222/ d4222 | 0.714 | 9.3% decrease |
|  |  | d4222/wt vs. d4222/d4222 | 0.043 | 31.3% decrease |
| *Tubb2b* D4185 | 0.653 (0.530) |  | N/A |  |
