# Supplementary material for: Differential requirements of tubulin genes in mammalian forebrain development
Source: PLoS Genet. 2019 Aug 6;15(8):e1008243. doi: 10.1371/journal.pgen.1008243 (PMC6697361; doi:10.1371/journal.pgen.1008243)
Supplement: S6 Table — (DOCX) [file pgen.1008243.s019.docx]

**S6 Table.** Statistical analysis of width of ventricular zone, intermediate zone, and cortical plate, in *Tuba1a* deletion alleles.

|  | **ANOVA F statistic (p value)** |  | **Tukey’s multiple comparison adjusted P value** | **Mean difference**  increase  decrease |
| --- | --- | --- | --- | --- |
| **Ventricular Zone** | | | | |
| *Tuba1a* D4304 | 261.2 (p<0.0001) | wt vs. D4304/wt | 0.999 | 0.00% |
|  |  | wt vs. D4304/ D4304 | <0.0001 | 115.1% increase |
|  |  | D4304/wt vs. D4304/D4304 | <0.0001 | 115.1% increase |
| *Tuba1a* D4262 | 346.6 (p<0.0001) | wt vs. D4262/wt | 0.085 | 24.8% increase |
|  |  | wt vs. D4262/ D4262 | <0.0001 | 179% increase |
|  |  | D4262/wt vs. D4262/D4262 | <0.0001 | 154% increase |
| **Intermediate Zone** | | | | |
| *Tuba1a* D4304 | 135.3 (p<0.0001) | wt vs. D4304/wt | 0.815 | 0.20% decrease |
|  |  | wt vs. D4304/ D4304 | <0.0001 | 46.2% decrease |
|  |  | D4304/wt vs. D4304/D4304 | <0.0001 | 48.3% decrease |
| *Tuba1a* D4262 | 272.0 (p<0.0001) | wt vs. D4262/wt | 0.338 | 0.60% decrease |
|  |  | wt vs. D4262/ D4262 | <0.0001 | 56.9% decrease |
|  |  | D4262/wt vs. D4262/D4262 | <0.0001 | 50.9% decrease |
| **Cortical Plate** | | | | |
| *Tuba1a* D4304 | 72.81 (p<0.0001) | wt vs. D4304/wt | 0.981 | 0.58% decrease |
|  |  | wt vs. D4304/ D4304 | <0.0001 | 31.5% decrease |
|  |  | D4304/wt vs. D4304/D4304 | <0.0001 | 32.0% decrease |
| *Tuba1a* D4262 | 71.24 (p<0.0001) | wt vs. D4262/wt | 0.242 | 7.89% decrease |
|  |  | wt vs. D4262/ D4262 | <0.0001 | 37.1% decrease |
|  |  | D4262/wt vs. D4262/D4262 | <0.0001 | 29.2% decrease |
