# Supplementary material for: Differential requirements of tubulin genes in mammalian forebrain development
Source: PLoS Genet. 2019 Aug 6;15(8):e1008243. doi: 10.1371/journal.pgen.1008243 (PMC6697361; doi:10.1371/journal.pgen.1008243)
Supplement: S7 Table — (DOCX) [file pgen.1008243.s020.docx]

**S7 Table.** Neurogenesis and migration in *Tuba1a* mutants

|  | **Wild-type** | **d4304/d4304** | **p-value** | **Mean difference**  **increase**  **decrease** | **Wild-type** | **quas/quas** | **p-value** | **Mean difference**  **increase**  **decrease** |  |
| --- | --- | --- | --- | --- | --- | --- | --- | --- | --- |
| pHH3+ mitotic cells in VZ (cells/μm^2^ x 10^4^) | | | | | | | | | |
| E14.5 | 5.09 | 4.90 | 0.515 | 3.6% decrease | 3.385e-4 | 4.025e4 | 0.0211 | 18.9% increase |  |
| E16.5 | 4.63 | 5.36 | 0.017 | 15.7% increase | 4.199e-4 | 3.895e4 | 0.3325 | 7.2% decrease |  |
| E13.5-E14.5 EdU pulse chase (cells/μm^2^ x 10^4^) | | | | | | | | | |
| EdU+ Ki67- | 17.18 | 14.50 | <0.0001 | 15.6% decrease | 0.001578 | 0.001614 | 0.4806 | 2.3% increase |  |
| EdU+ Ki67+ | 0.31 | 0.055 | <0.0001 | 84.3% increase | 6.097e-5 | 7.069e-5 | 0.1588 | 15.9% increase |  |
| EdU- Ki67+ | 3.71 | 5.82 | 0.0003 | 56.7% increase | 6.54e-4 | 7.605e-4 | 0.1013 | 16.3% increase |  |
| Quit Fraction (%) | 98.2 | 96.2 | <0.0001 | 2% decrease | 0.9631 | 0.9577 | 0.1816 | 0.56% decrease |  |
| E13.5-E14.5 EdU pulse chase: Radial migration (% cell in bin/ total cells) | | | | | | | | | |
| Bin 3 (dorsal) | 23.45 | 24.15 | 0.383 | 3% increase | 0.2573 | 0.2221 | 0.0003 | 13.7% decrease |  |
| Bin 2 | 35.96 | 35.51 | 0.506 | 1.3% decrease | 0.3578 | 0.3933 | <0.0001 | 9.9% increase |  |
| Bin 1 (ventral) | 40.59 | 40.34 | 0.751 | 1% decrease | 0.3849 | 0.3847 | 0.9804 | 0.1% decrease |  |
| E13.5-E16.5 EdU pulse chase: Radial migration (% cell in bin/ total cells) | | | | | | | | | |
| Bin 3 (dorsal) | 40.86 | 26.98 | <0.0001 | 34% decrease | 0.3574 | 0.2308 | <0.0001 | 35.4% decrease |  |
| Bin 2 | 26.60 | 37.35 | <0.0001 | 40.4% increase | 0.2442 | 0.3769 | <0.0001 | 54.3% increase |  |
| Bin 1 (ventral) | 32.54 | 35.67 | 0.001 | 9.6% increase | 0.3984 | 0.3923 | 0.4927 | 1.5% decrease |  |
| TBR2+ intermediate progenitor (cells/um^2^ x 10^4^) | | | | | | | | | |
| E14.5 Tbr2 | 94.43 | 94.02 | 0.9713 | 0.4% decrease | 0.009933 | 0.009378 | 0.3880 | 5.6% decrease |  |
| E16.5 Tbr2 | 23.07 | 26.18 | 0.0743 | 13.5% increase | 0.003775 | 0.004585 | 0.0043 | 21.5% increase |  |
| Apoptotic cells (cells/um^2^ x 10^4^) | | | | | | | | | |
| E14.5 | 0.054 | 0.095 | 0.0192 | 75.9% increase | 0.037 | 0.011 | 0.0005 | 185% increase |  |
| E16.5 | 0.032 | 0.313 | <0.0001 | 866% increase | 0.024 | 0.435 | <0.0001 | 1721% increase |  |
