# Supplementary material for: Differential requirements of tubulin genes in mammalian forebrain development
Source: PLoS Genet. 2019 Aug 6;15(8):e1008243. doi: 10.1371/journal.pgen.1008243 (PMC6697361; doi:10.1371/journal.pgen.1008243)
Supplement: S8 Table — (DOCX) [file pgen.1008243.s021.docx]

**S8 Table.** Statistical analysis of immunoblotting for total α-tubulin and β-tubulin in *Tuba1a* mutants.

|  | **ANOVA p value** |  | **Tukey’s multiple comparison adjusted P value** | **Effect size** |
| --- | --- | --- | --- | --- |
| **pan α-tubulin/GAPDH** | | | | |
| *d4353* | 0.159 |  |  |  |
| *quas* | <0.0001 | Wt vs. *quas*/wt | 0.0003 | 41% decrease |
|  |  | Wt vs. *quas*/*quas* | <0.0001 | 66.5% decrease |
|  |  | *quas*/wt vs. *quas*/*quas* | 0.0145 | 43% decrease |
| **Pan α-tubulin/Revert** | | | | |
| *d4353* | <0.0001 | wt vs. *d4353*/wt | 0.271 | 13 % increase |
|  |  | wt vs. *d4353/ d4353* | 0.006 | 26 % decease |
|  |  | *d4353*/wt vs. *d4353/d4353* | <0.0001 | 34.4 % decrease |
| *quas* | 0.001 | wt vs. *quas*/wt | 0.1096 | 32 % decrease |
|  |  | wt vs. *quas/quas* | 0.0009 | 69 % decrease |
|  |  | *quas*/wt vs*. quas/quas* | 0.045 | 54.4 % decrease |
| **Pan β tubulin/GAPDH** | | | | |
| *d4353* | 0.0002 | wt vs. *d4353*/wt | 0.41 | 16 % increase |
|  |  | wt vs. *d4353/ d4353* | 0.0021 | 52 % decrease |
|  |  | *d4353*/wt vs. *d4353/d4353* | 0.0002 | 58.7 % decrease |
| *quas* | 0.0002 | wt vs. *quas*/wt | 0.33 | 19.8 % decrease |
|  |  | wt vs. *quas/quas* | 0.0002 | 64 % decrease |
|  |  | *quas*/wt vs*. quas/quas* | 0.112 | 55 %decrease |
| **Pan β tubulin/ Revert^TM^** | | | | |
| *Tubba1a* | 0.016 | wt vs. *d4353*/wt | 0.93 | 4.1% increase |
|  |  | wt vs. *d4353/ d4353* | 0.031 | 38% decrease |
|  |  | *d4353*/wt vs. *d4353/d4353* | 0.02 | 40% decrease |
| *quas* | 0.0007 | wt vs. *quas*/wt | 0.042 | 26 % decrease |
|  |  | wt vs. *quas/quas* | 0.0006 | 46% decrease |
|  |  | *quas*/wt vs*. quas/quas* | 0.07 | 27.6% decrease |
